# Supplementary material for: Hederagenin’s uric acid-lowering effects in hyperuricemic mice: Mechanistic insights from molecular docking and in vivo analysis
Source: PLoS One. 2025 Jun 24;20(6):e0326317. doi: 10.1371/journal.pone.0326317 (PMC12186911; doi:10.1371/journal.pone.0326317)
Supplement: S1 File — (DOCX) [file pone.0326317.s001.docx]

Table 1 PCR primer sequences and protocols

| Gene | Forward primer (5'-3') | Reverse primer (5'-3') | Size (bp) | Temperature (℃) | Thermal cycling |
| --- | --- | --- | --- | --- | --- |
| OAT1 | CACCTGCTAATGCCAACCTC | CCATTGTGCGGGAAAGGAAA | 20 | 56 | 35 |
| OAT3 | CTGCCTTCTTCATCTTCTCCTTG | CTTCCTCCTTCTTGCCGTTG | 135 | 56 | 35 |
| ABCG2 | CAGTTCTCAGCAGCTCTTCGAC | TCCTCCAGAGATGCCACGGATA | 147 | 59 | 35 |
| URAT1 | GGAGGAACCAAGCAGGGACAAA | CCGTAGAAGGTGAAGCCAAAGG | 123 | 59 | 35 |
| GLUT9 | GCCATCATTGCCTCGTTCTGCA | TACGGCGAAGTTTGAGAGCCAG | 135 | 60 | 35 |
| GAPDH | CATCACTGCCACCCAGAAGACTG | ATGCCAGTGAGCTTCCCGTTCAG | 153 | 60 | 35 |
