# Supplementary figures and images for: Hederagenin’s uric acid-lowering effects in hyperuricemic mice: Mechanistic insights from molecular docking and in vivo analysis
Source: PLoS One. 2025 Jun 24;20(6):e0326317. doi: 10.1371/journal.pone.0326317 (PMC12186911; doi:10.1371/journal.pone.0326317)

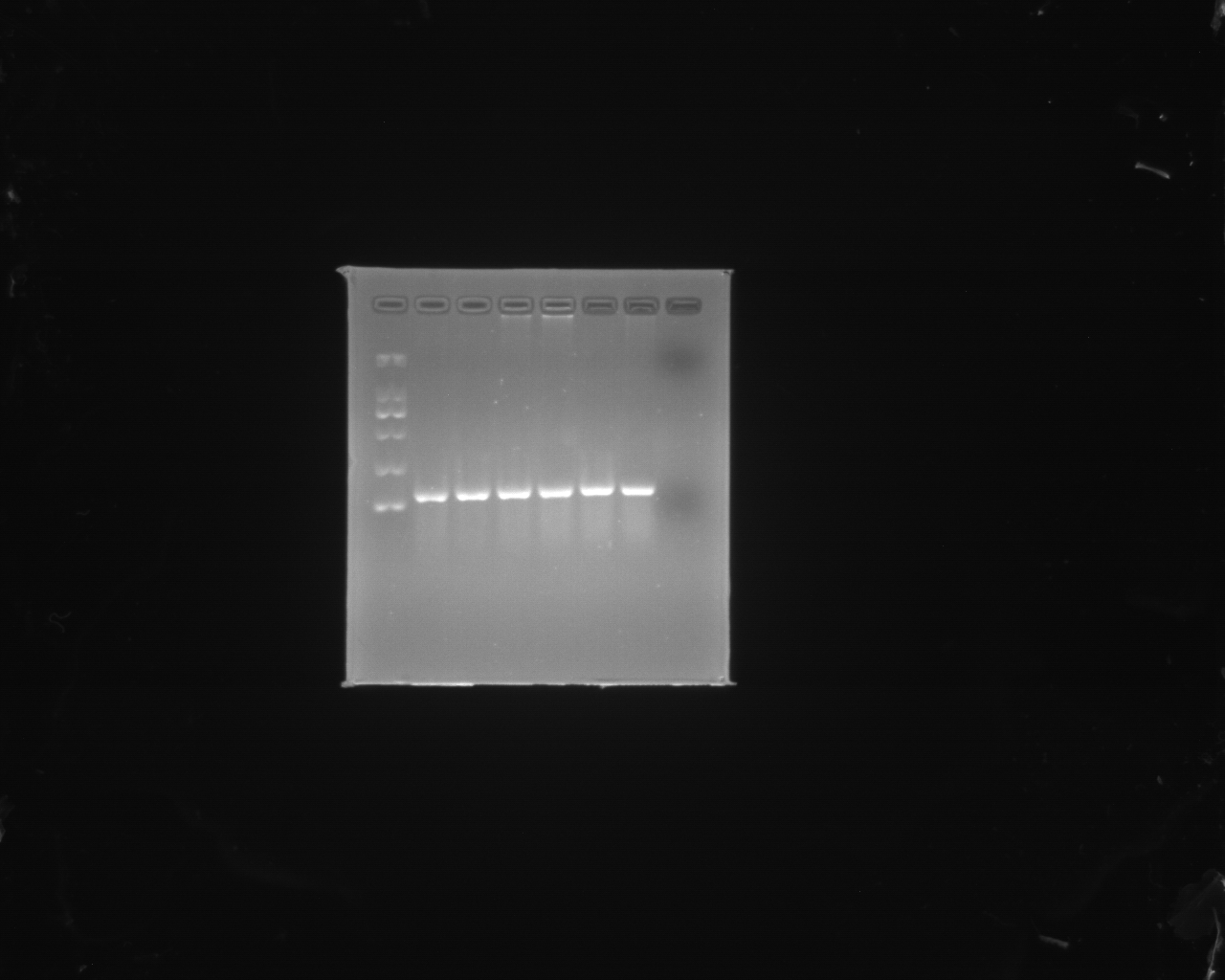

Supplement: S2 File — (ZIP) [file pone.0326317.s002.zip › Supporting Information PCR data/ABCG2/2022-10-28 ABCG2最新 1.tif]

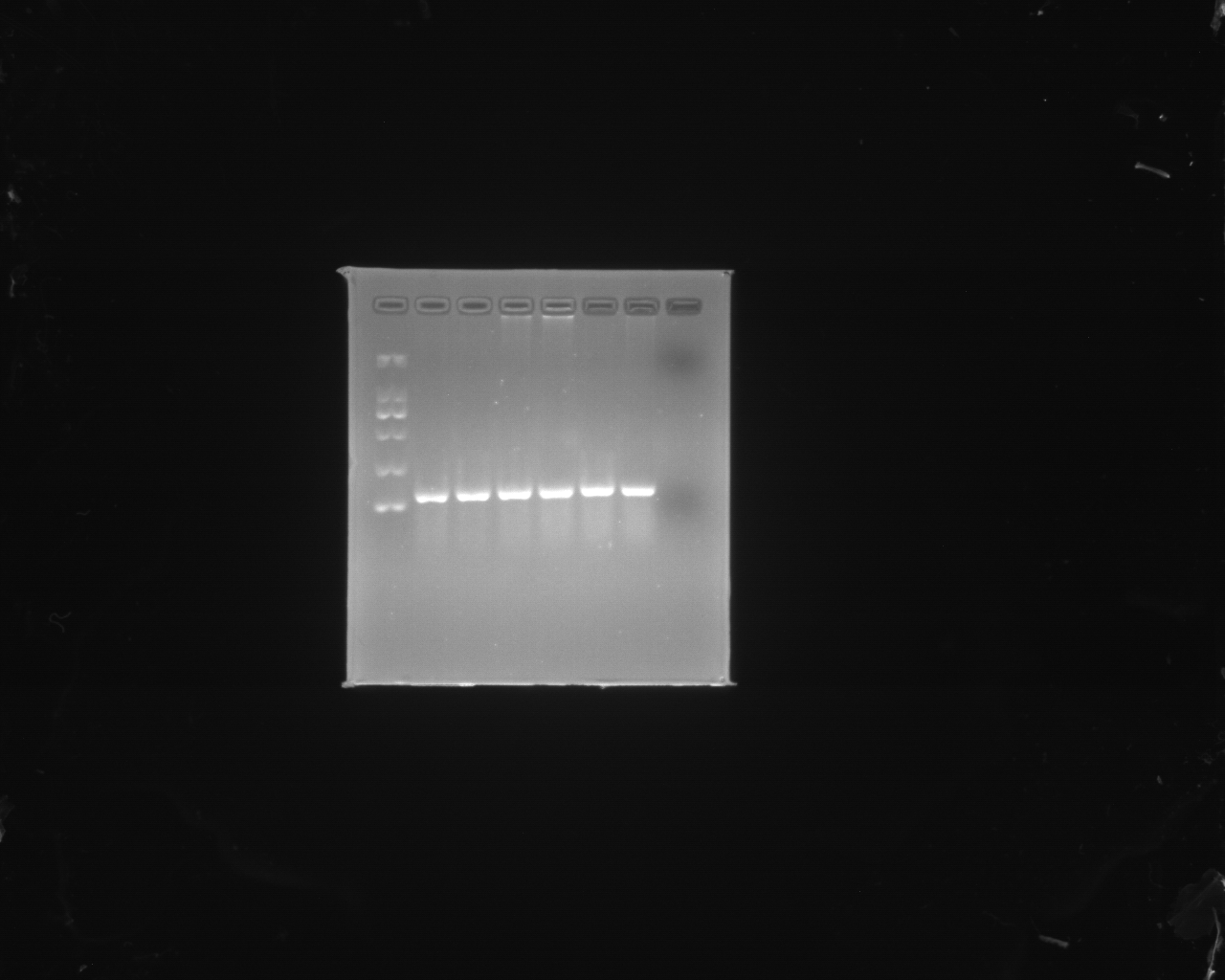

Supplement: S2 File — (ZIP) [file pone.0326317.s002.zip › Supporting Information PCR data/ABCG2/2022-10-28 ABCG2最新 2.tif]

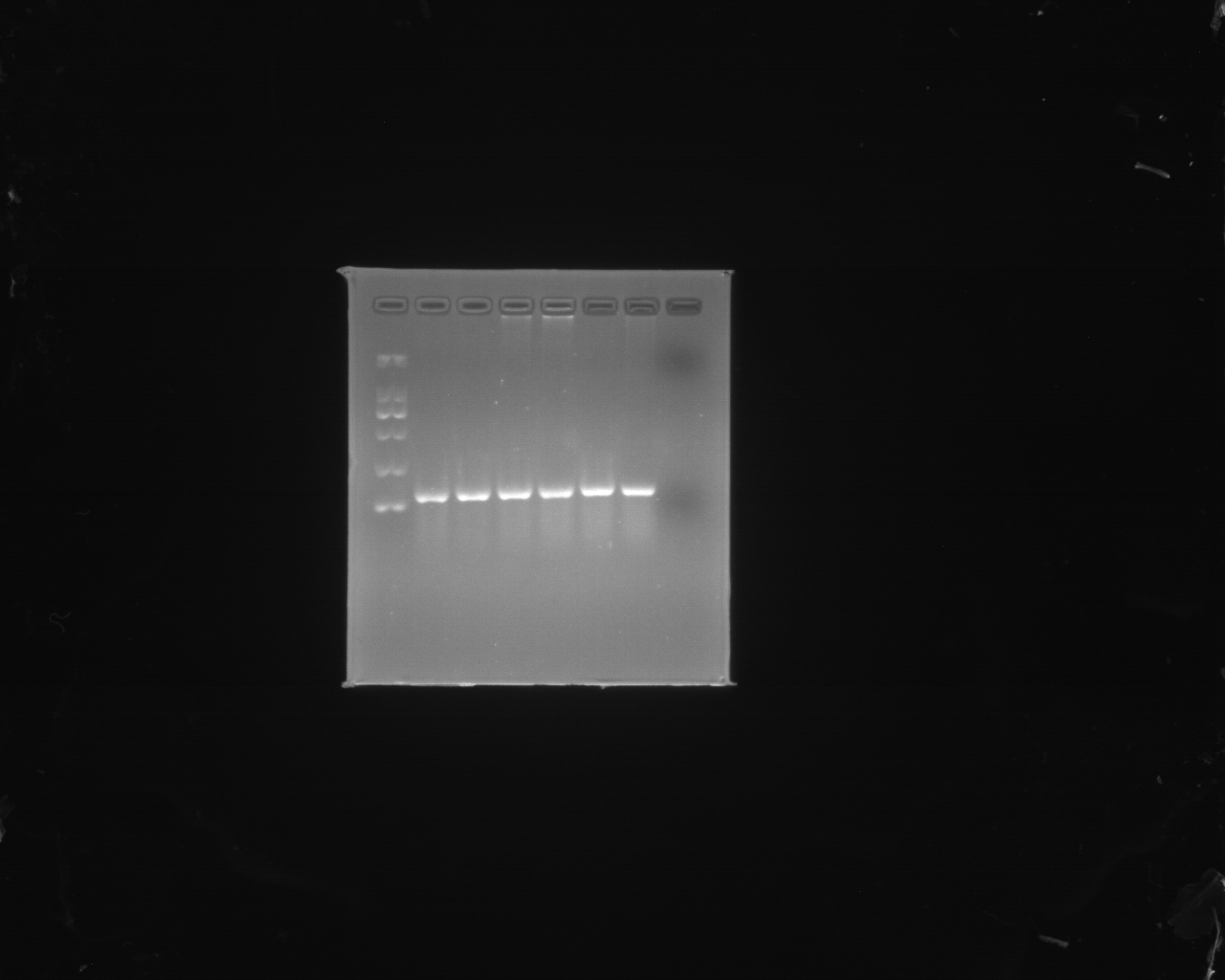

Supplement: S2 File — (ZIP) [file pone.0326317.s002.zip › Supporting Information PCR data/ABCG2/2022-10-28 ABCG2最新 3.tif]

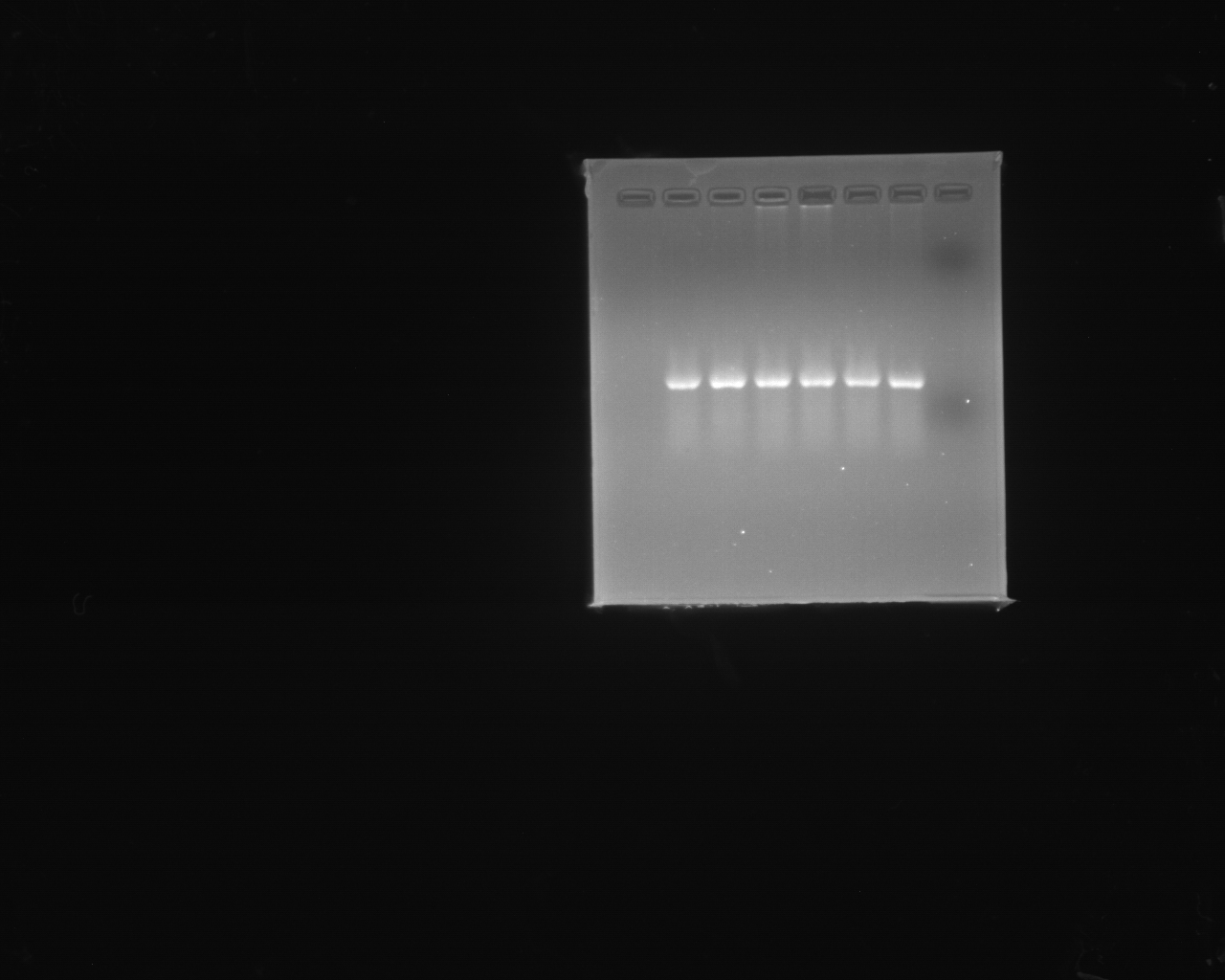

Supplement: S2 File — (ZIP) [file pone.0326317.s002.zip › Supporting Information PCR data/ABCG2/2022-10-28 GAPDH1(5) 2.tif]

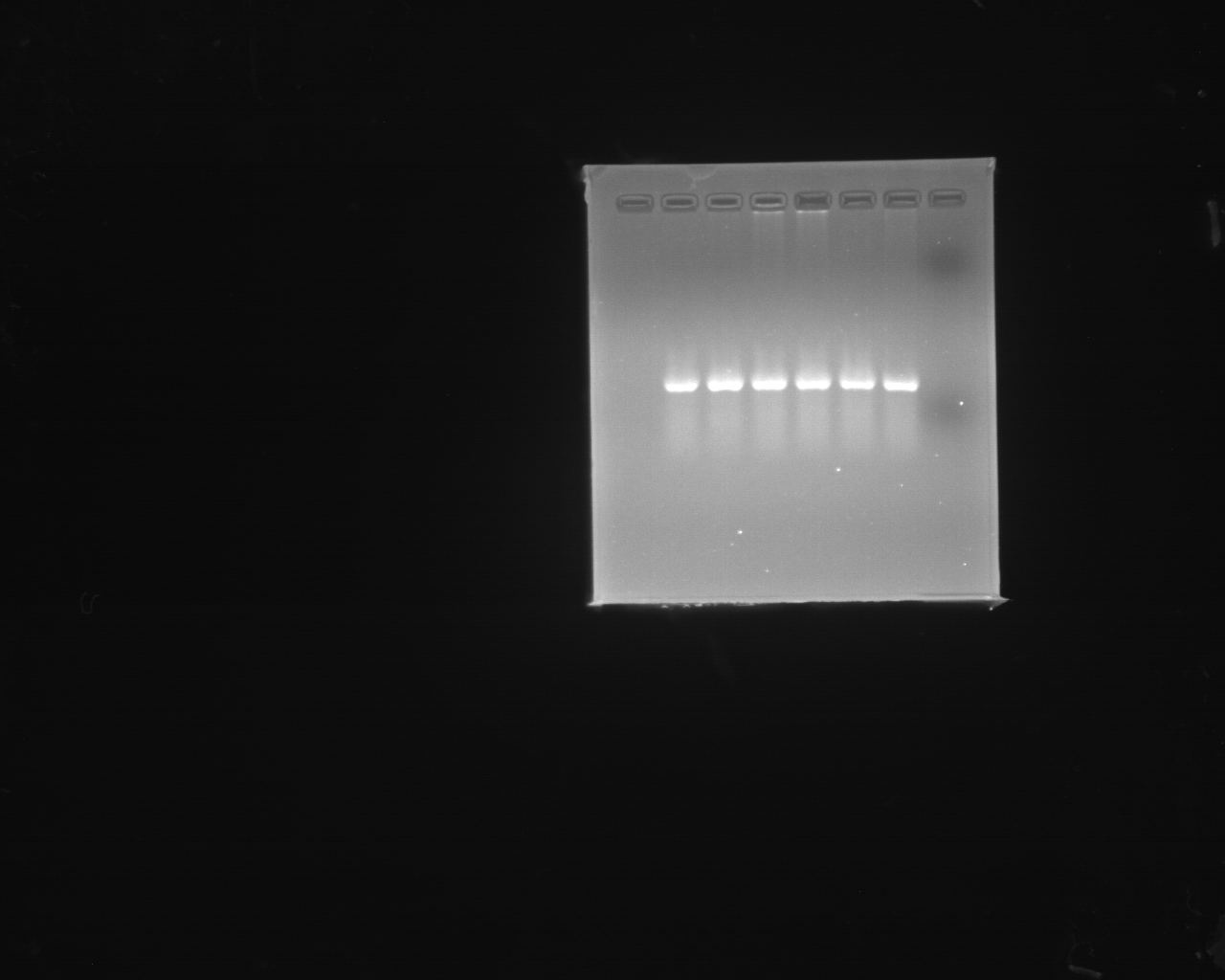

Supplement: S2 File — (ZIP) [file pone.0326317.s002.zip › Supporting Information PCR data/ABCG2/2022-10-28 GAPDH1(5) 4.tif]

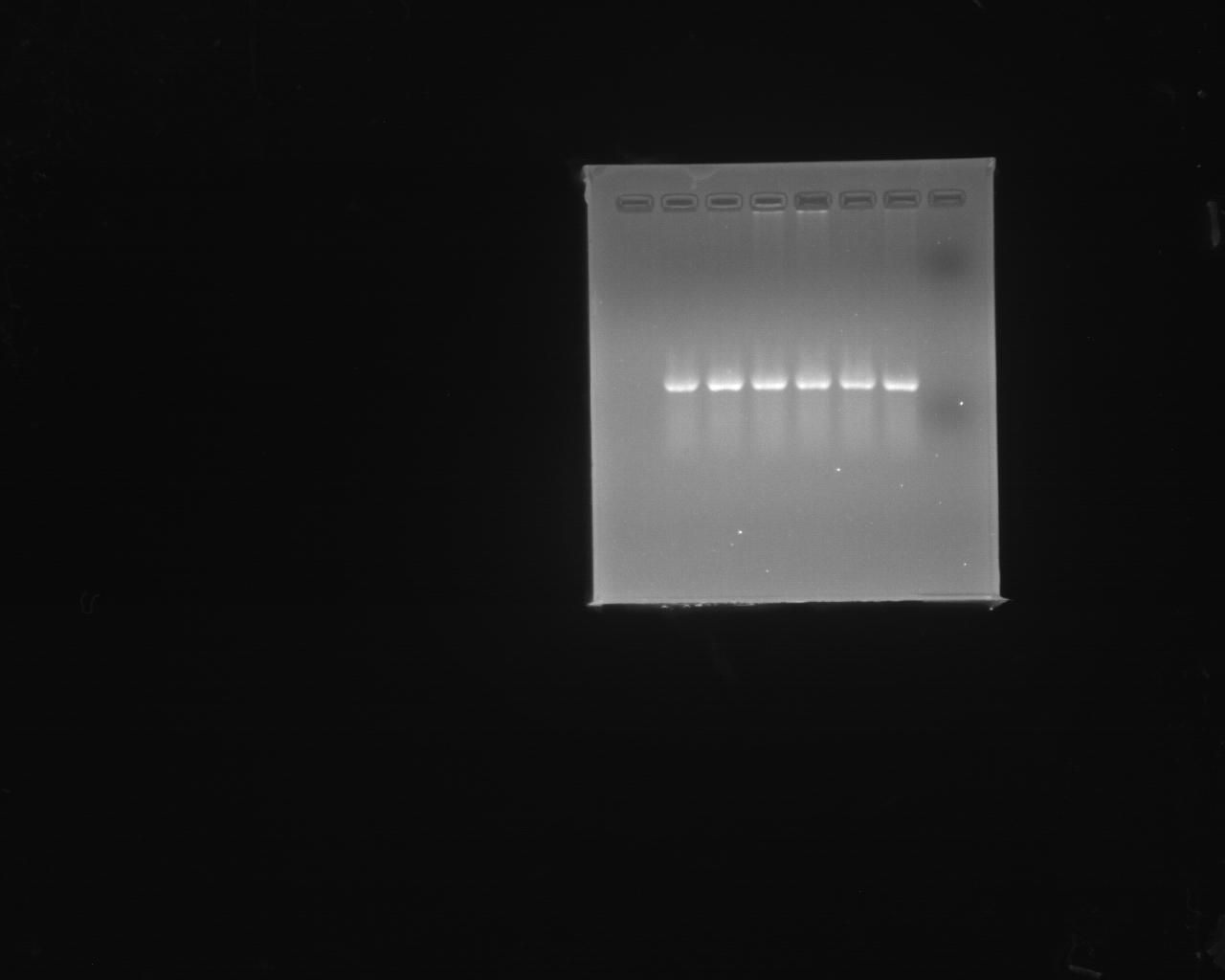

Supplement: S2 File — (ZIP) [file pone.0326317.s002.zip › Supporting Information PCR data/ABCG2/2022-10-28 GAPDH1(5) 5.tif]

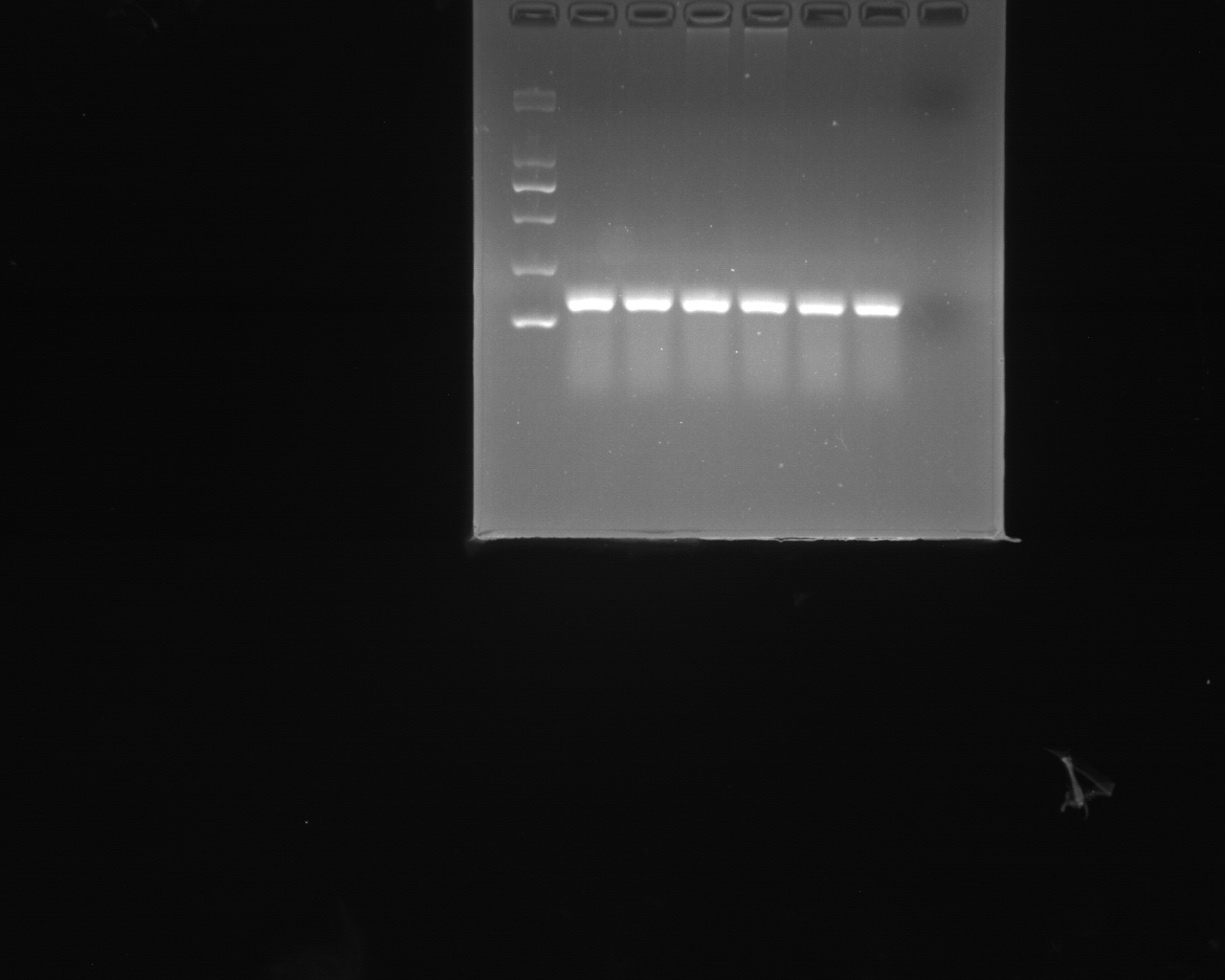

Supplement: S2 File — (ZIP) [file pone.0326317.s002.zip › Supporting Information PCR data/ABCG2/2022-11-12 GAPDH 1.tif]

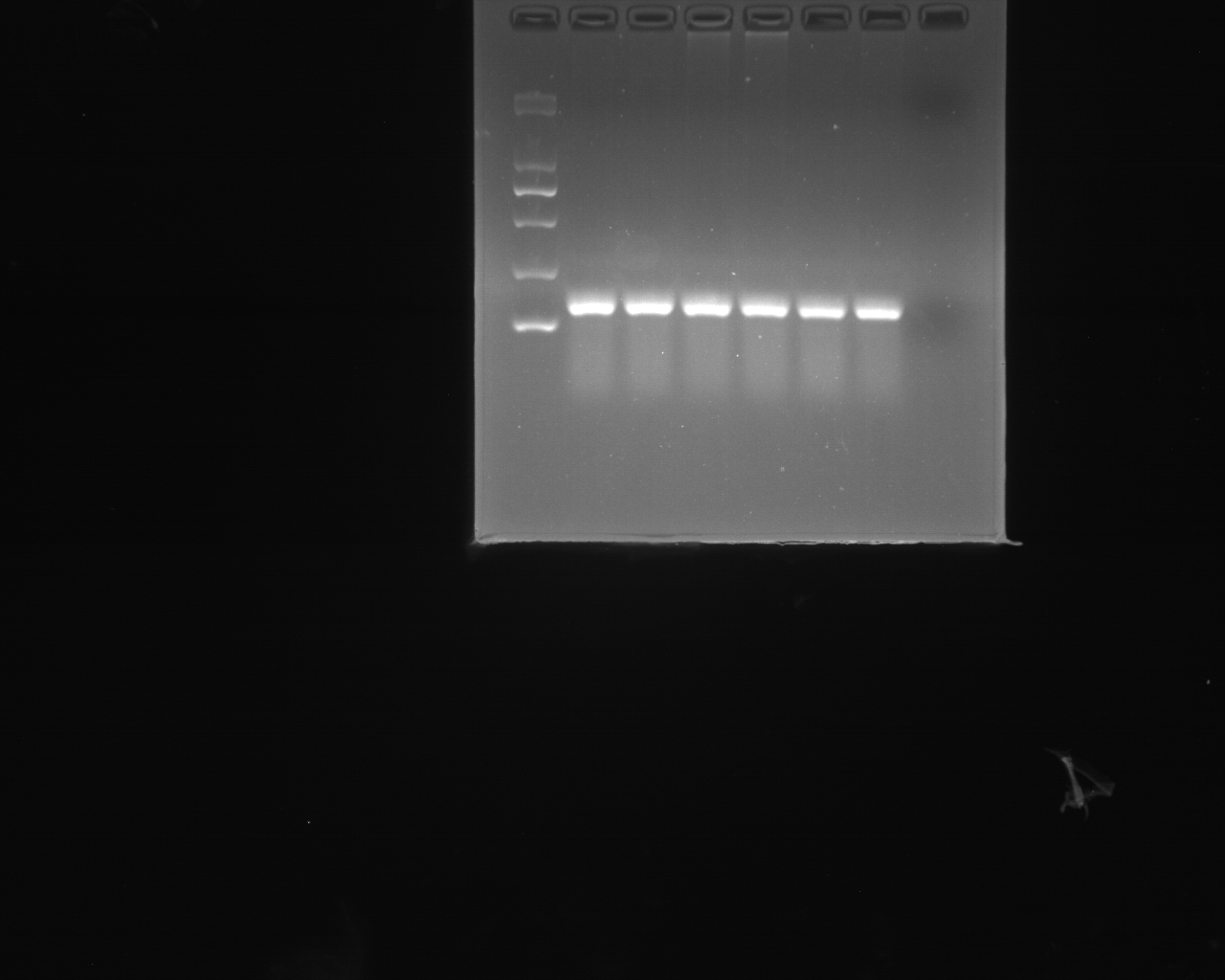

Supplement: S2 File — (ZIP) [file pone.0326317.s002.zip › Supporting Information PCR data/ABCG2/2022-11-12 GAPDH 2.tif]

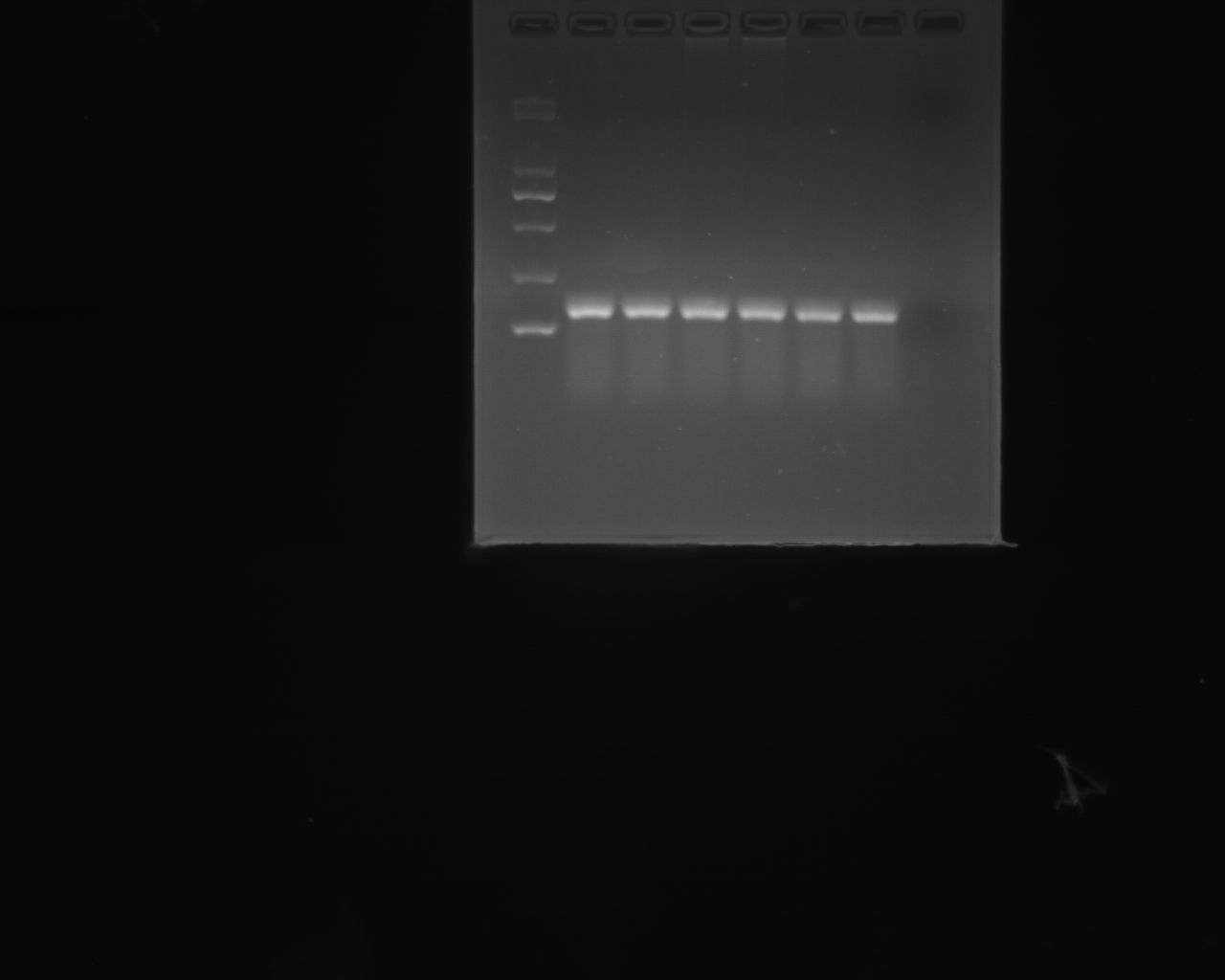

Supplement: S2 File — (ZIP) [file pone.0326317.s002.zip › Supporting Information PCR data/ABCG2/2022-11-12 GAPDH 3.tif]

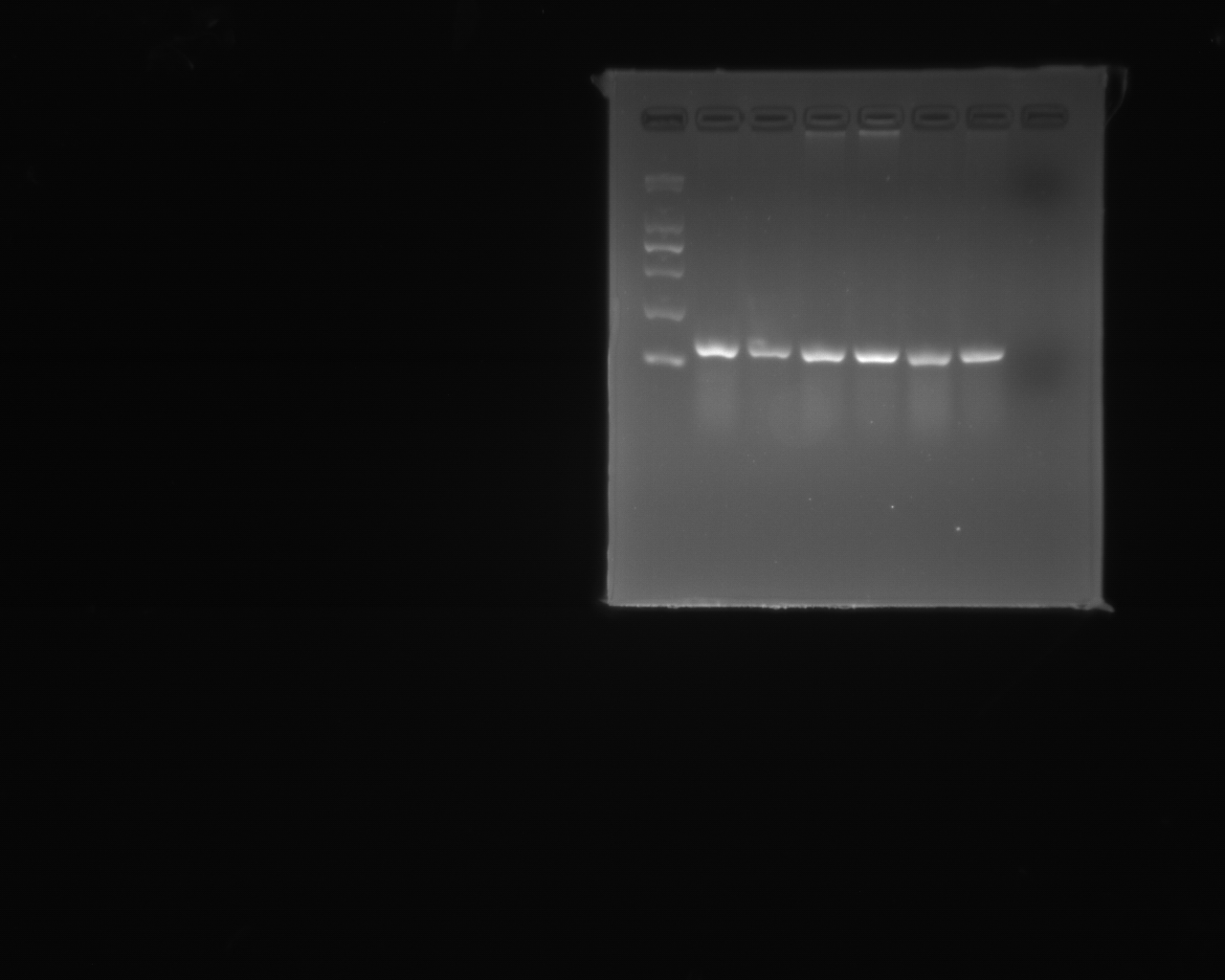

Supplement: S2 File — (ZIP) [file pone.0326317.s002.zip › Supporting Information PCR data/ABCG2/2022-11-13 ABCG2 t2成.tif]

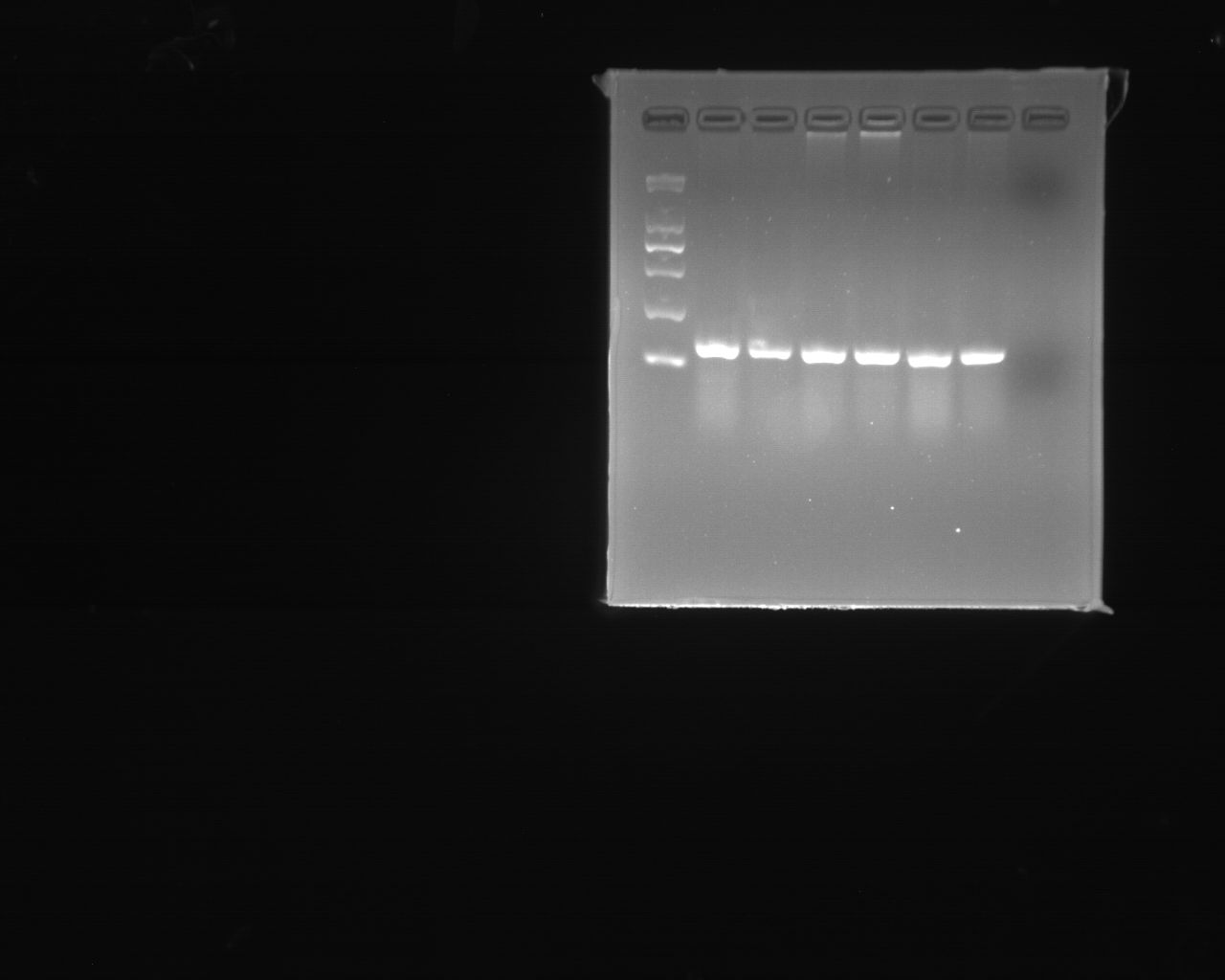

Supplement: S2 File — (ZIP) [file pone.0326317.s002.zip › Supporting Information PCR data/ABCG2/2022-11-13 ABCG2 t3成.tif]

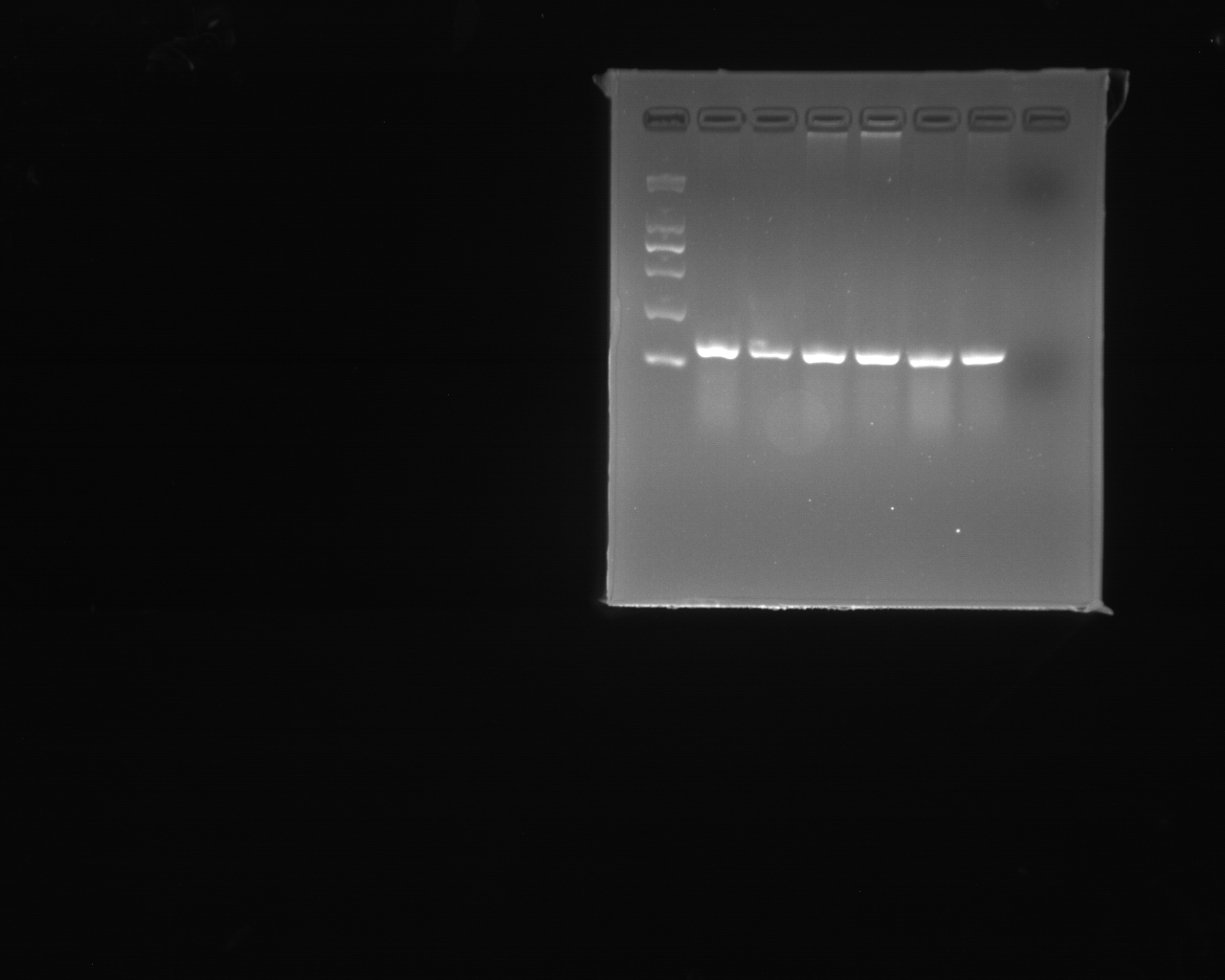

Supplement: S2 File — (ZIP) [file pone.0326317.s002.zip › Supporting Information PCR data/ABCG2/2022-11-13 ABCG2 t4成.tif]

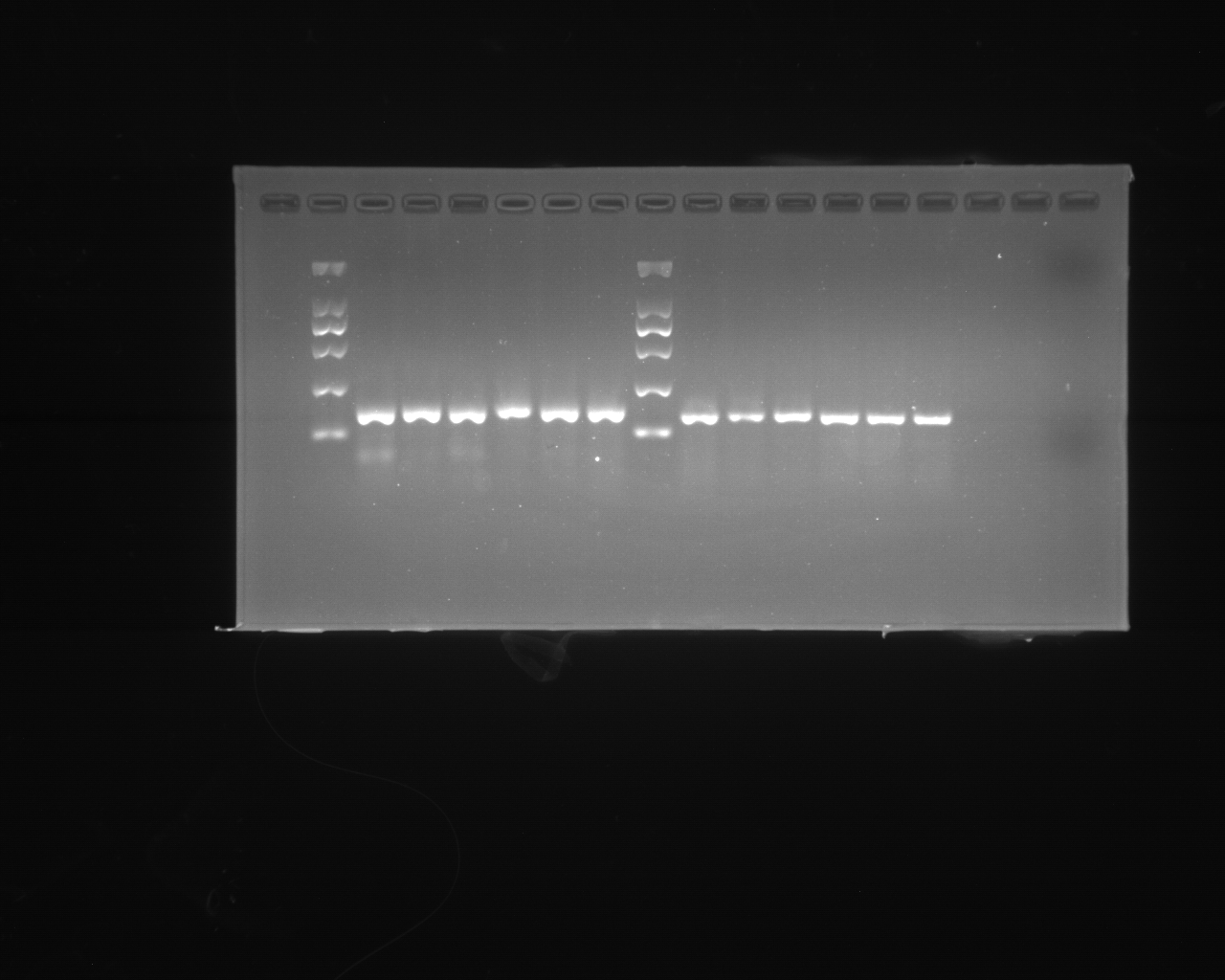

Supplement: S2 File — (ZIP) [file pone.0326317.s002.zip › Supporting Information PCR data/ABCG2/2022-9-15 GAPDH+ABcg2CG2 4.tif]

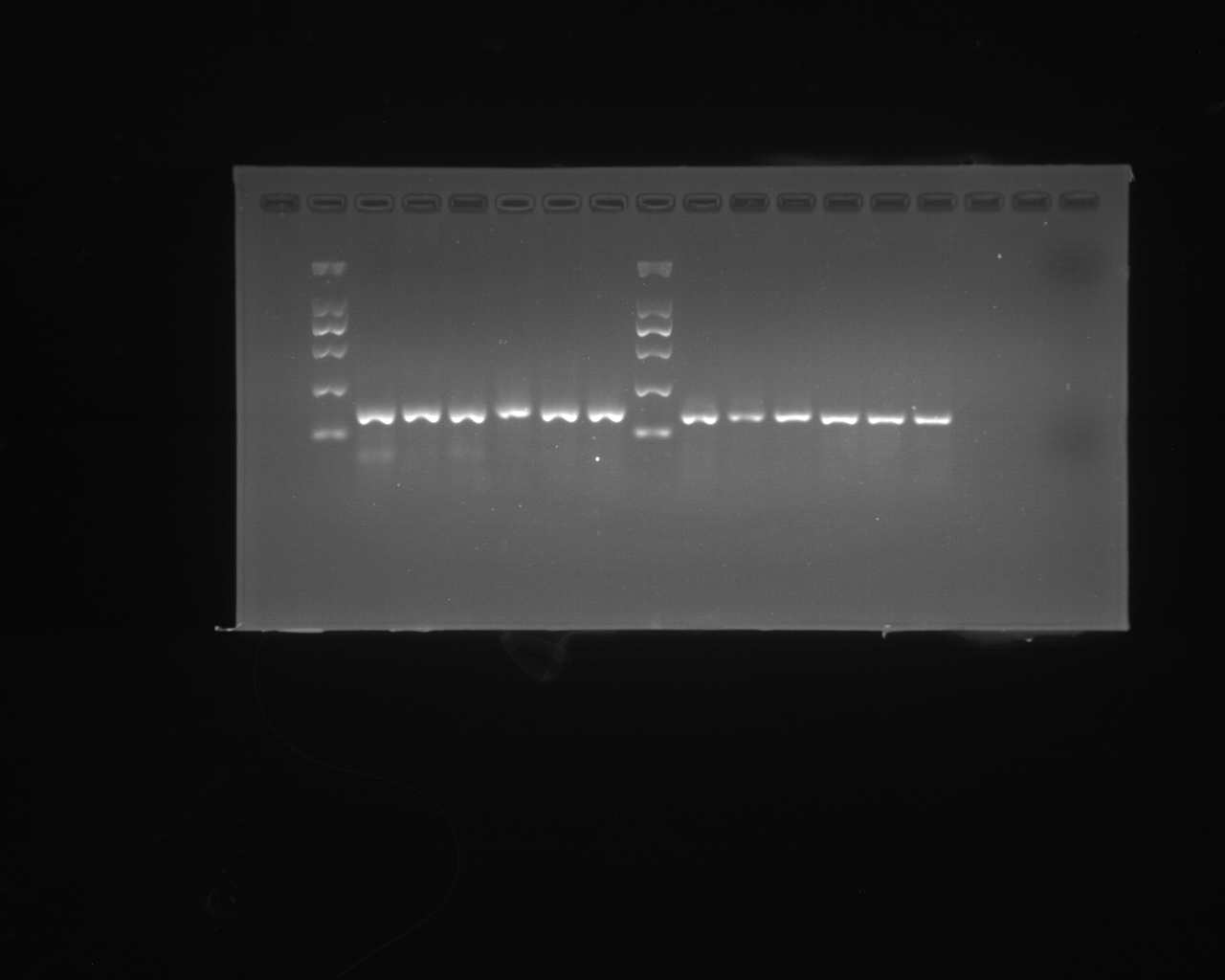

Supplement: S2 File — (ZIP) [file pone.0326317.s002.zip › Supporting Information PCR data/ABCG2/2022-9-15 GAPDH+ABcg2CG2 5.tif]

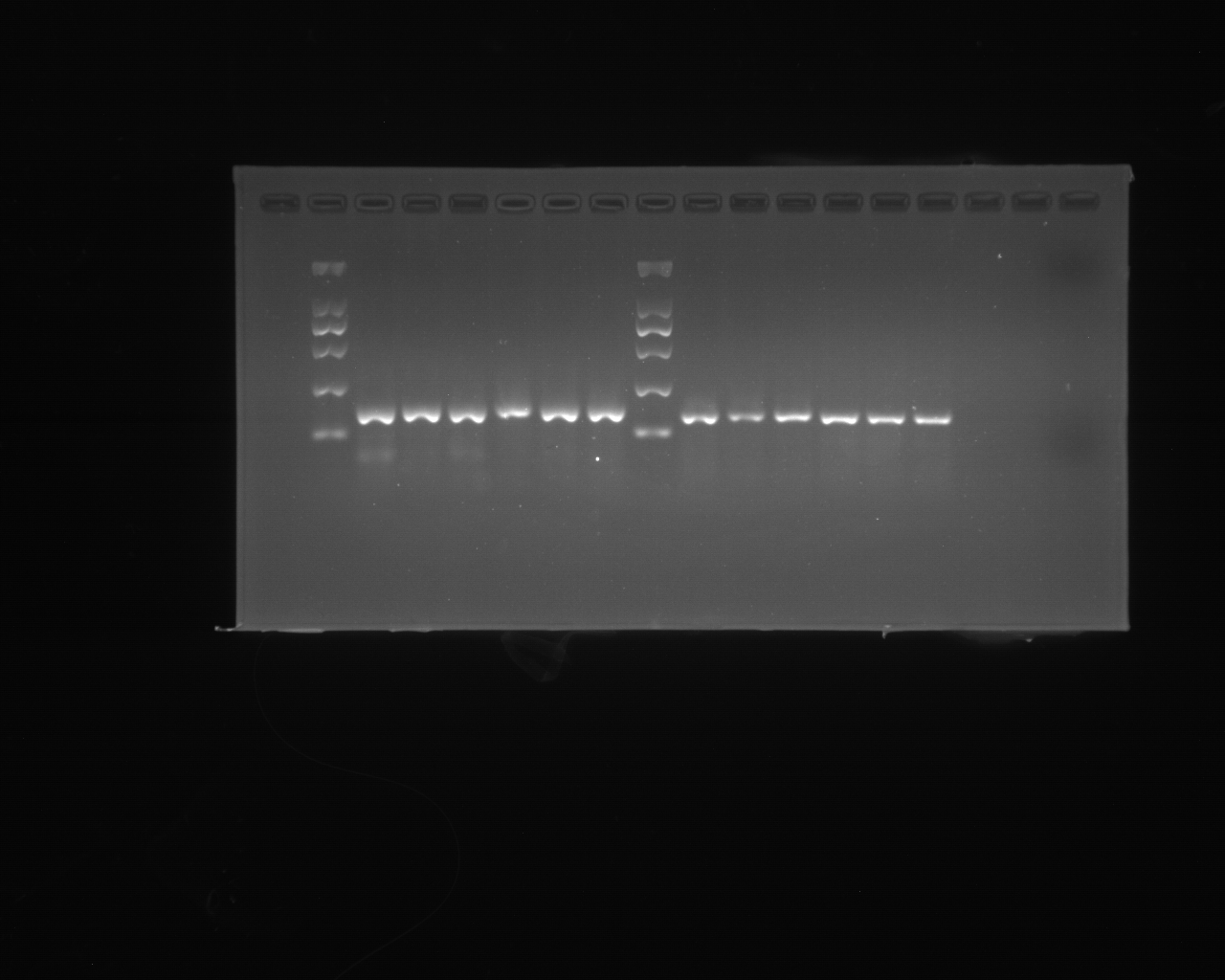

Supplement: S2 File — (ZIP) [file pone.0326317.s002.zip › Supporting Information PCR data/ABCG2/2022-9-15 GAPDH+ABcg2CG2 6.tif]

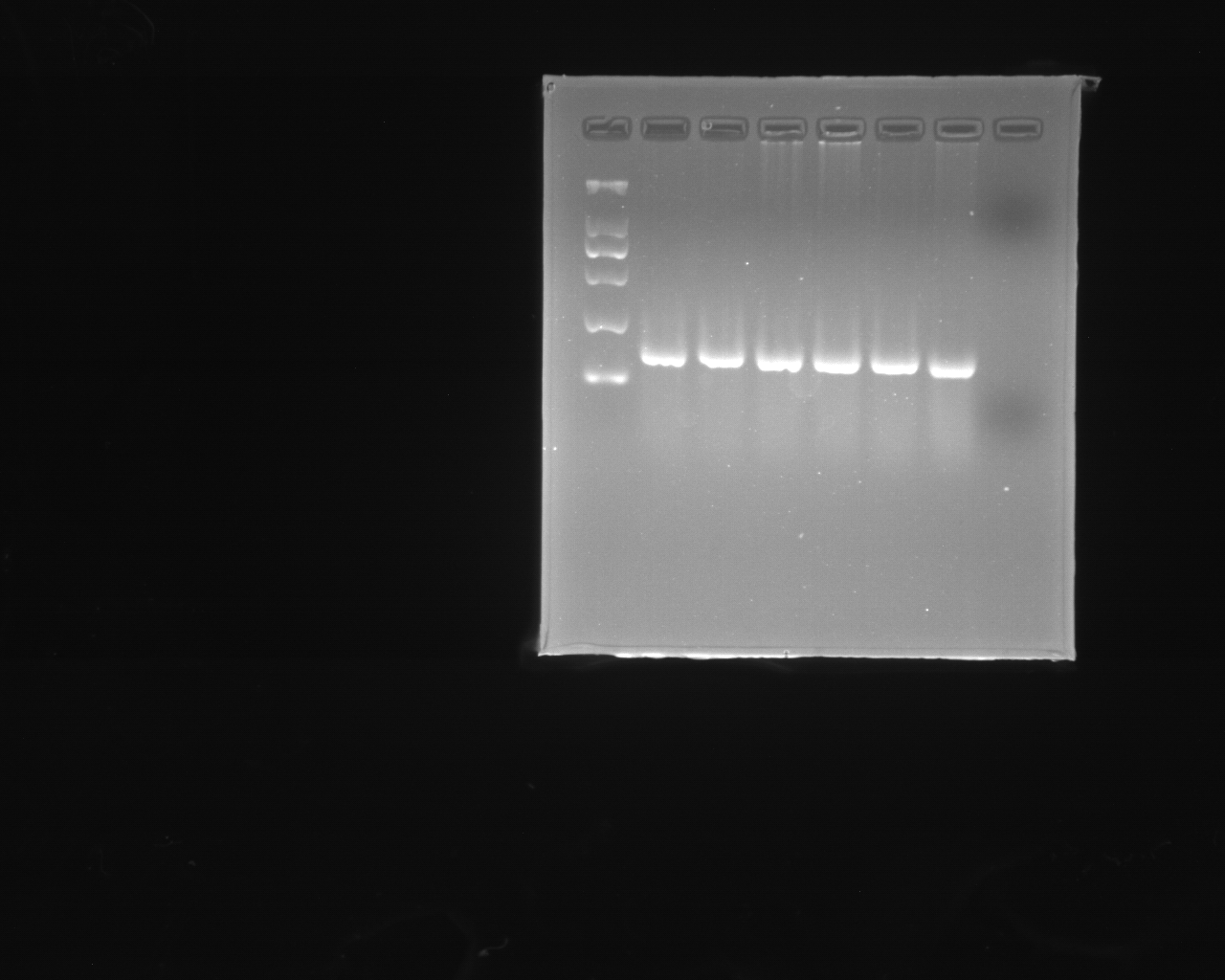

Supplement: S2 File — (ZIP) [file pone.0326317.s002.zip › Supporting Information PCR data/GLUT9/2022-11-4 GAPDH2 调1.tif]

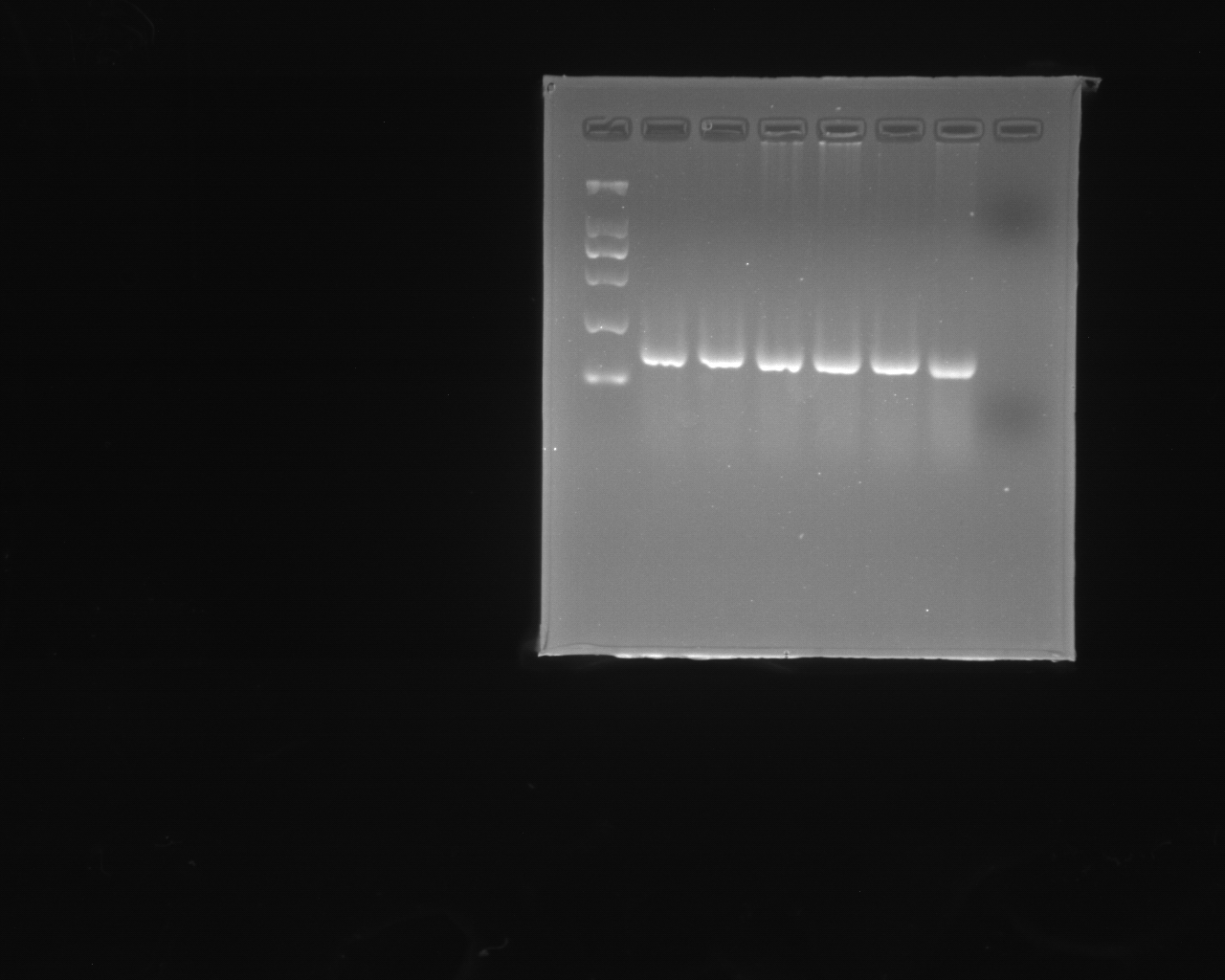

Supplement: S2 File — (ZIP) [file pone.0326317.s002.zip › Supporting Information PCR data/GLUT9/2022-11-4 GAPDH2 调2.tif]

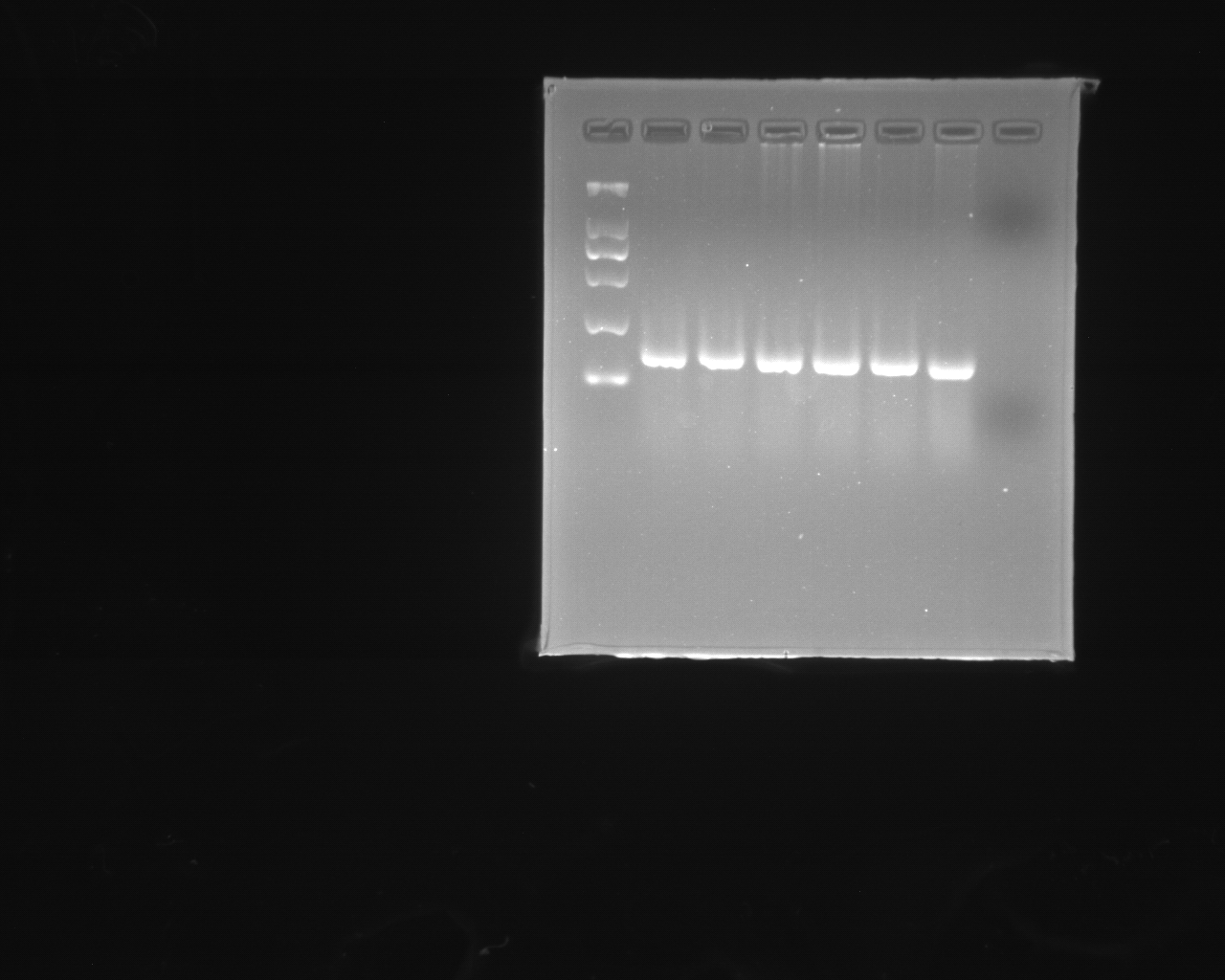

Supplement: S2 File — (ZIP) [file pone.0326317.s002.zip › Supporting Information PCR data/GLUT9/2022-11-4 GAPDH2 调3.tif]

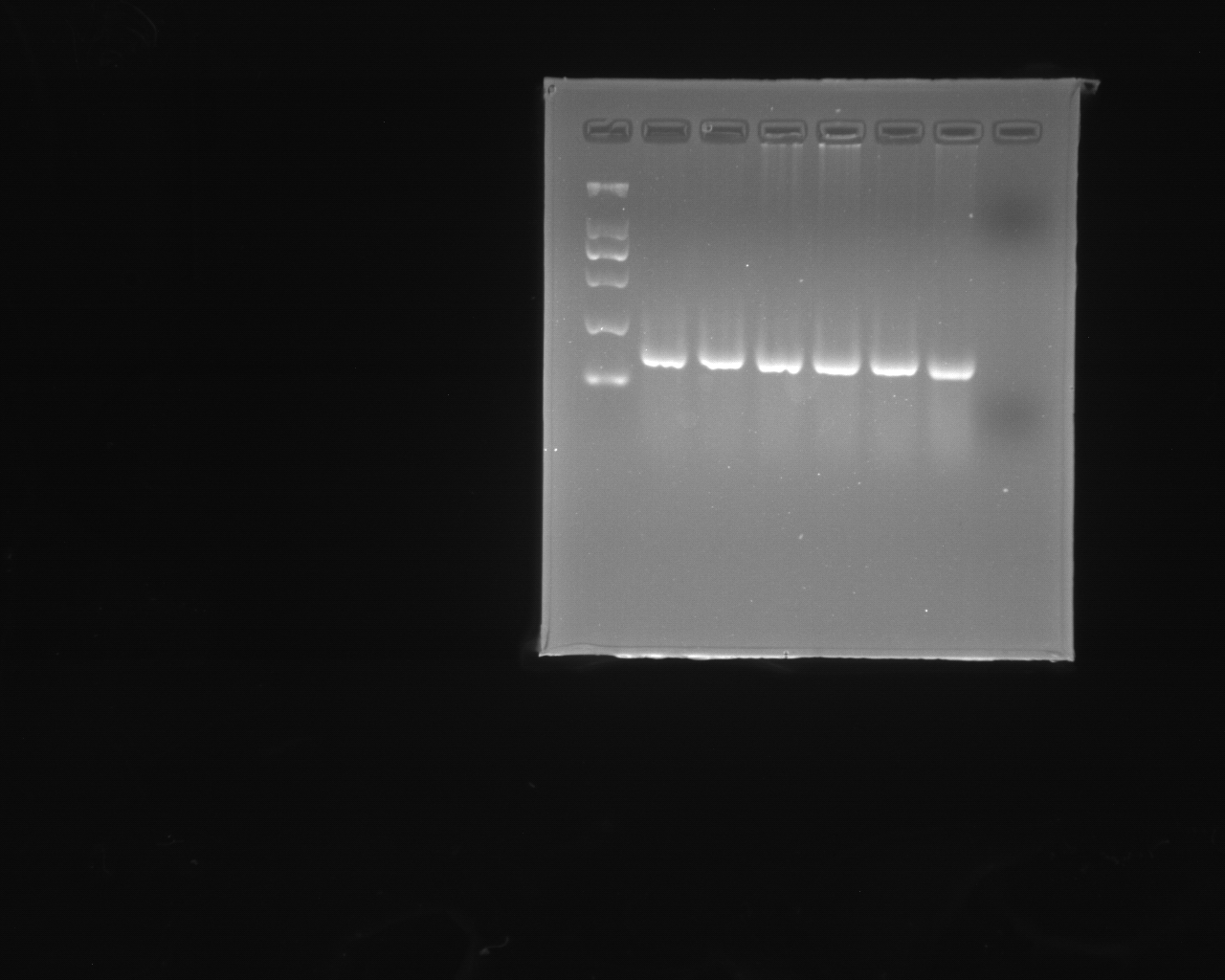

Supplement: S2 File — (ZIP) [file pone.0326317.s002.zip › Supporting Information PCR data/GLUT9/2022-11-4 GAPDH2 调4.tif]

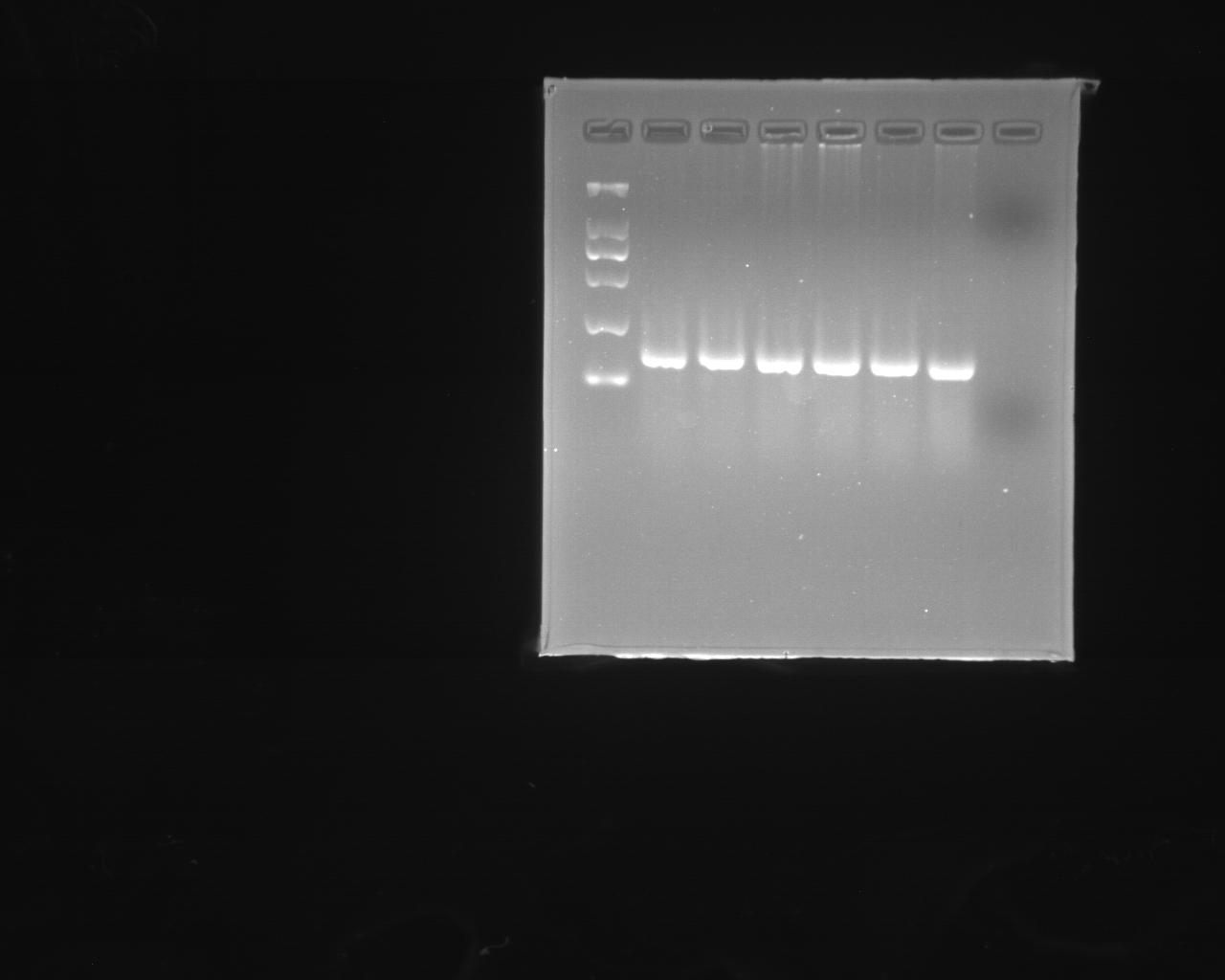

Supplement: S2 File — (ZIP) [file pone.0326317.s002.zip › Supporting Information PCR data/GLUT9/2022-11-4 GAPDH2 调5.tif]

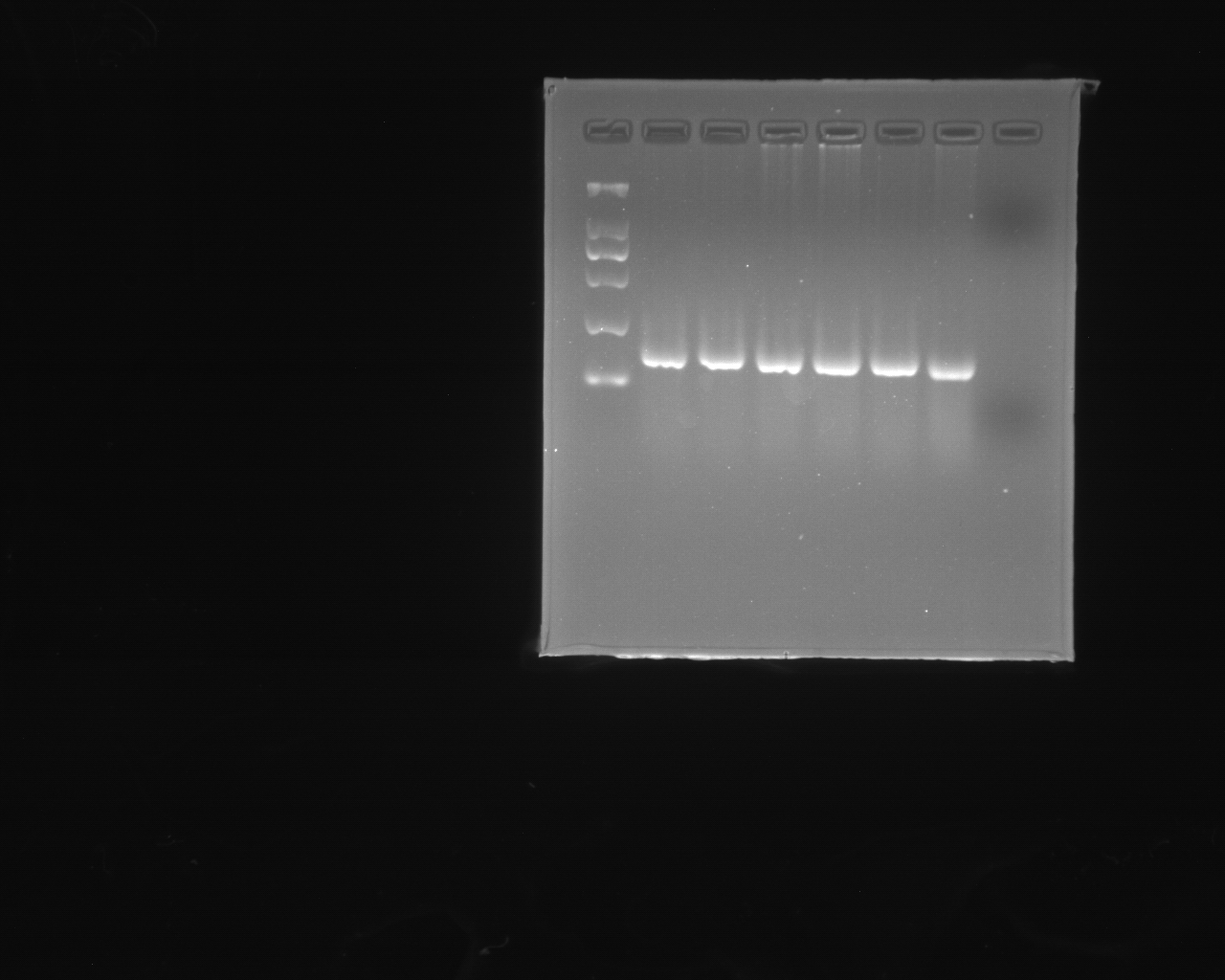

Supplement: S2 File — (ZIP) [file pone.0326317.s002.zip › Supporting Information PCR data/GLUT9/2022-11-4 GAPDH2 调6.tif]

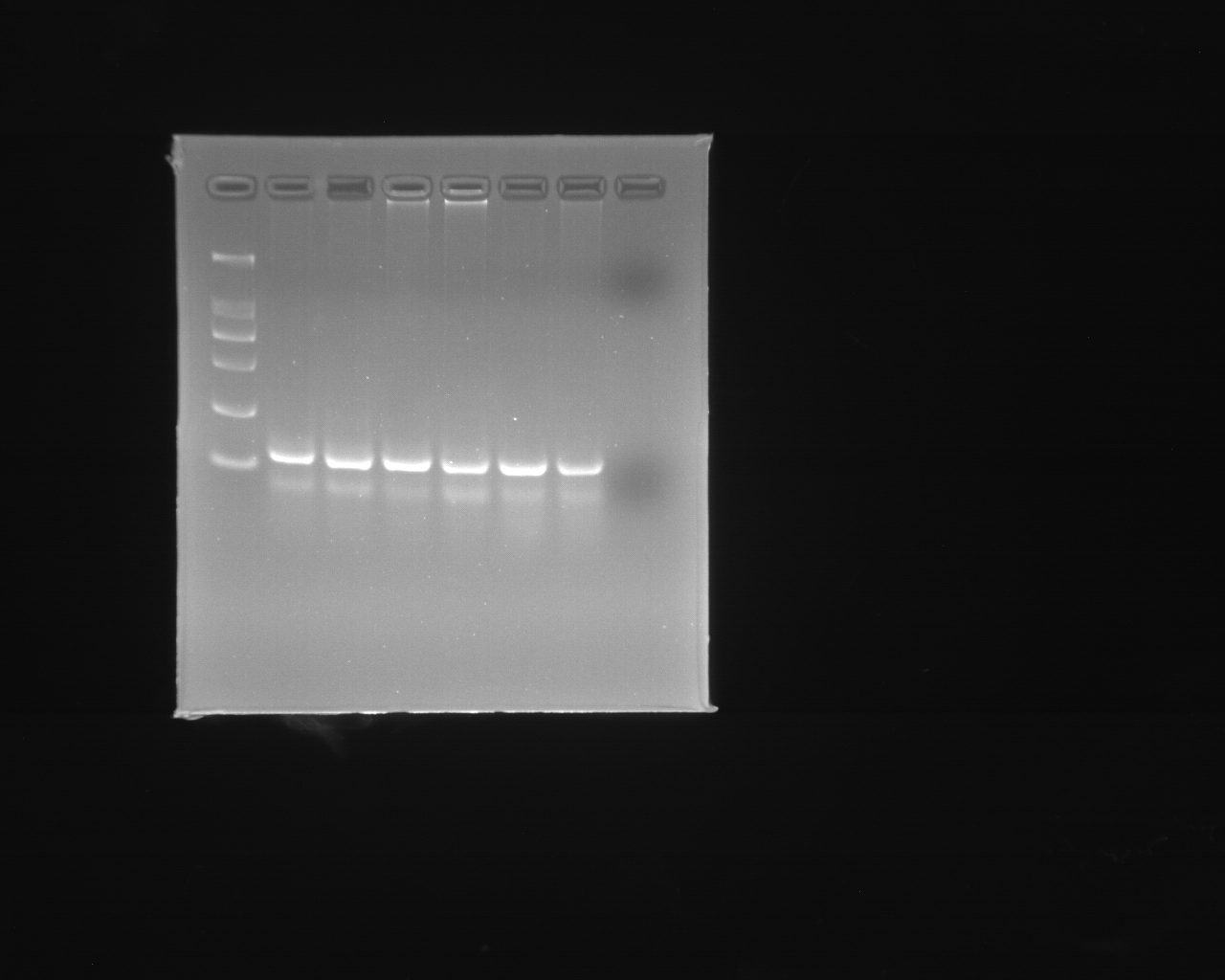

Supplement: S2 File — (ZIP) [file pone.0326317.s002.zip › Supporting Information PCR data/GLUT9/2022-11-4 GLUT9 1.tif]

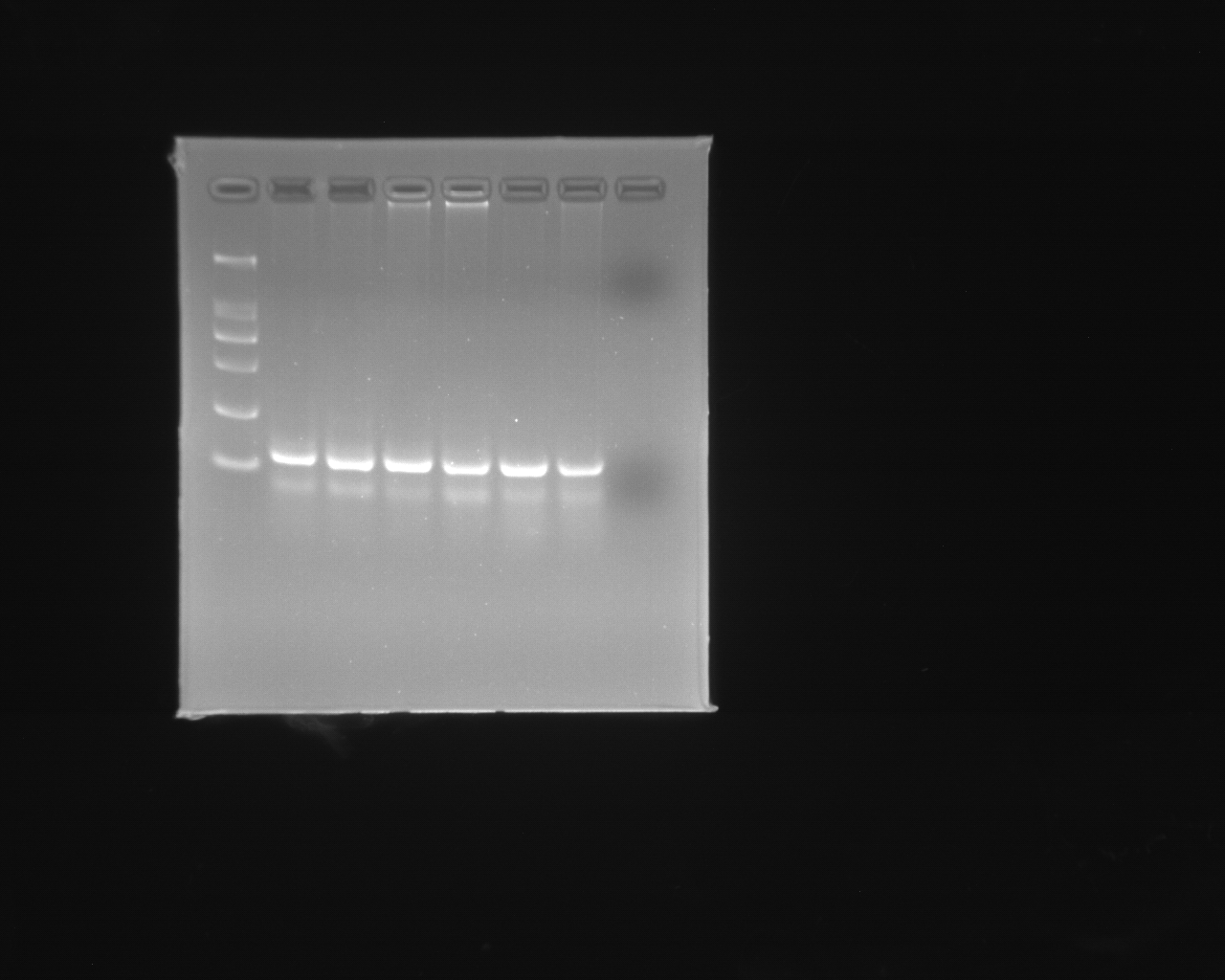

Supplement: S2 File — (ZIP) [file pone.0326317.s002.zip › Supporting Information PCR data/GLUT9/2022-11-4 GLUT9 5.tif]

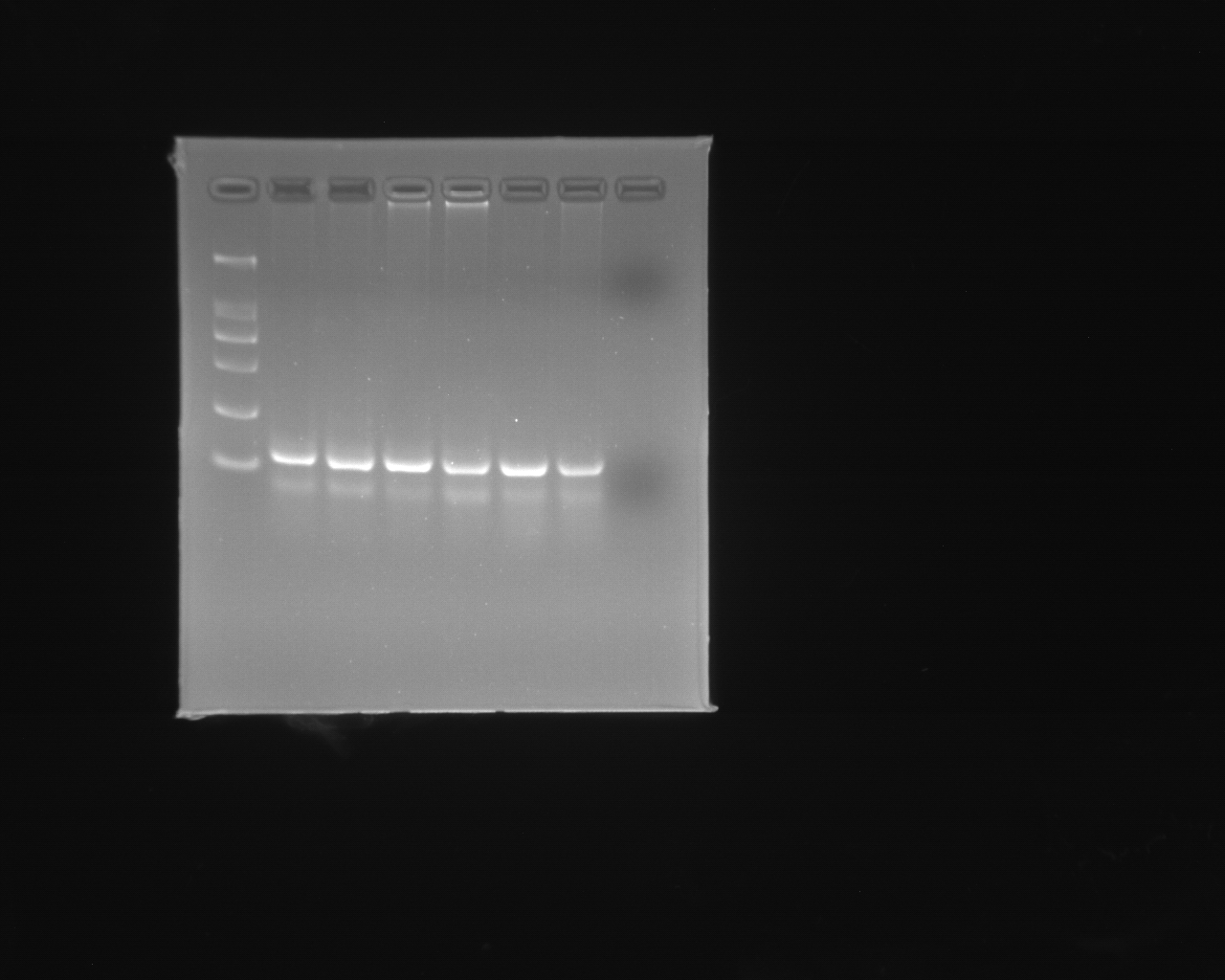

Supplement: S2 File — (ZIP) [file pone.0326317.s002.zip › Supporting Information PCR data/GLUT9/2022-11-4 GLUT9 6.tif]

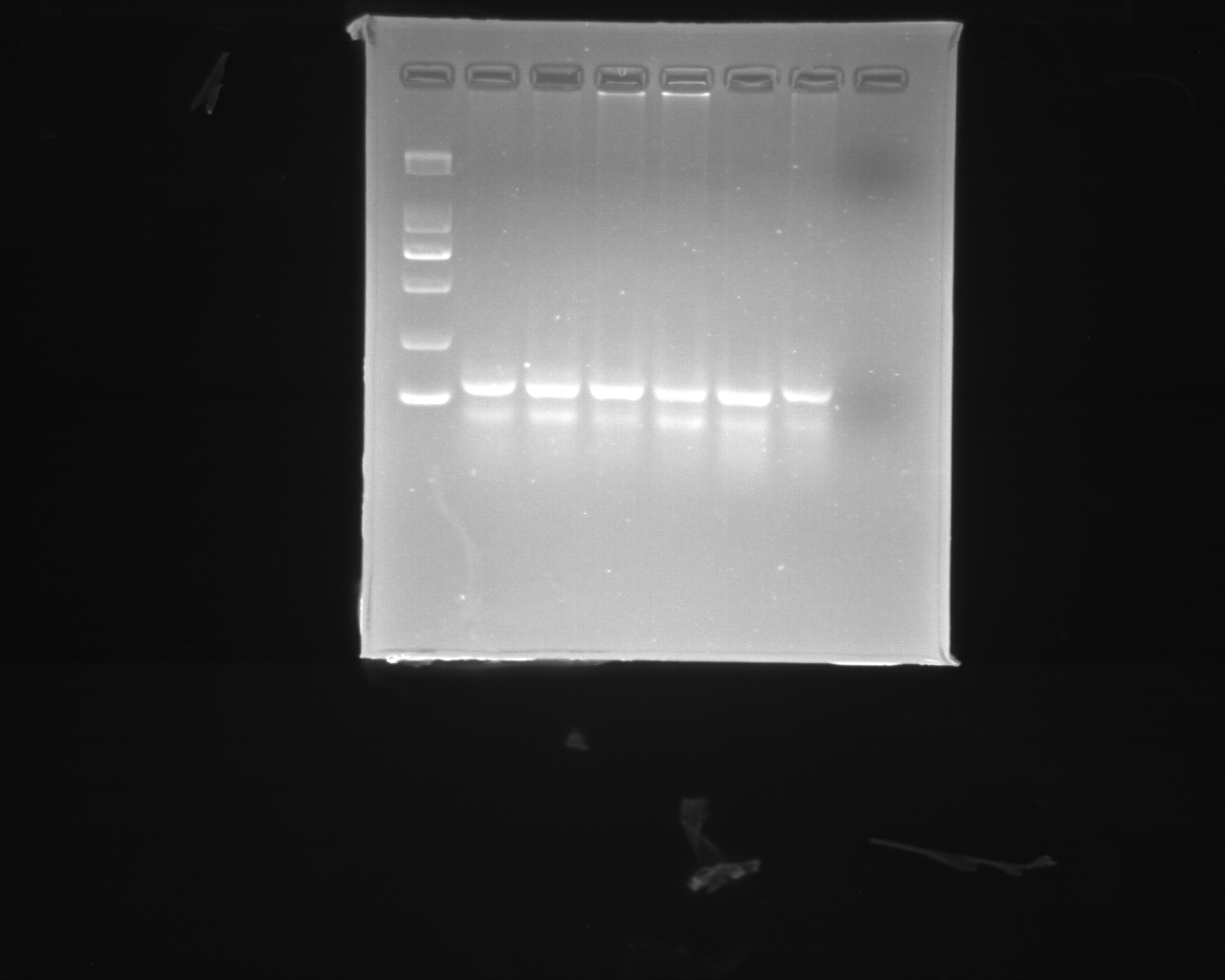

Supplement: S2 File — (ZIP) [file pone.0326317.s002.zip › Supporting Information PCR data/GLUT9/2022-11-6 GLUT9 1.tif]

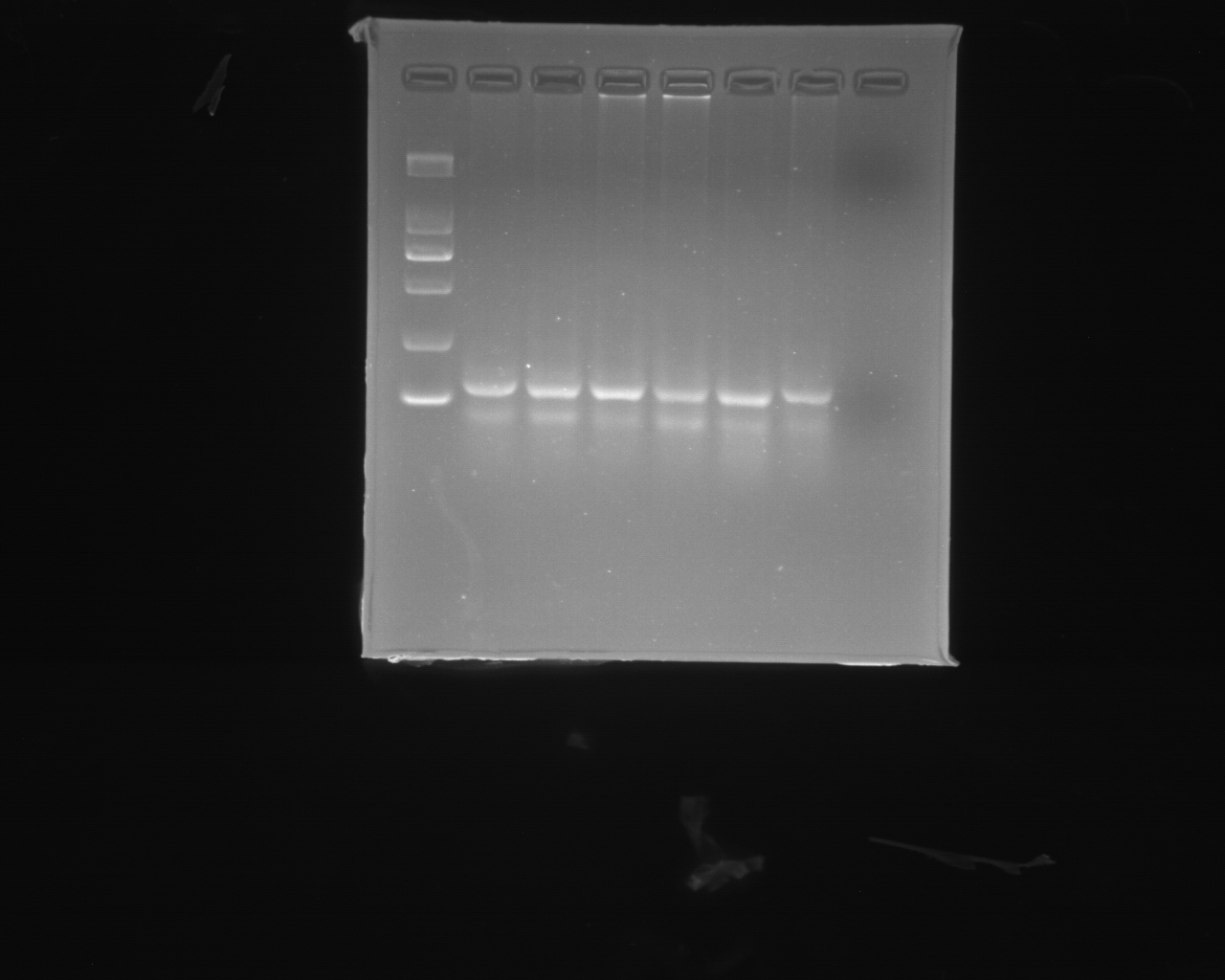

Supplement: S2 File — (ZIP) [file pone.0326317.s002.zip › Supporting Information PCR data/GLUT9/2022-11-6 GLUT9 4.tif]

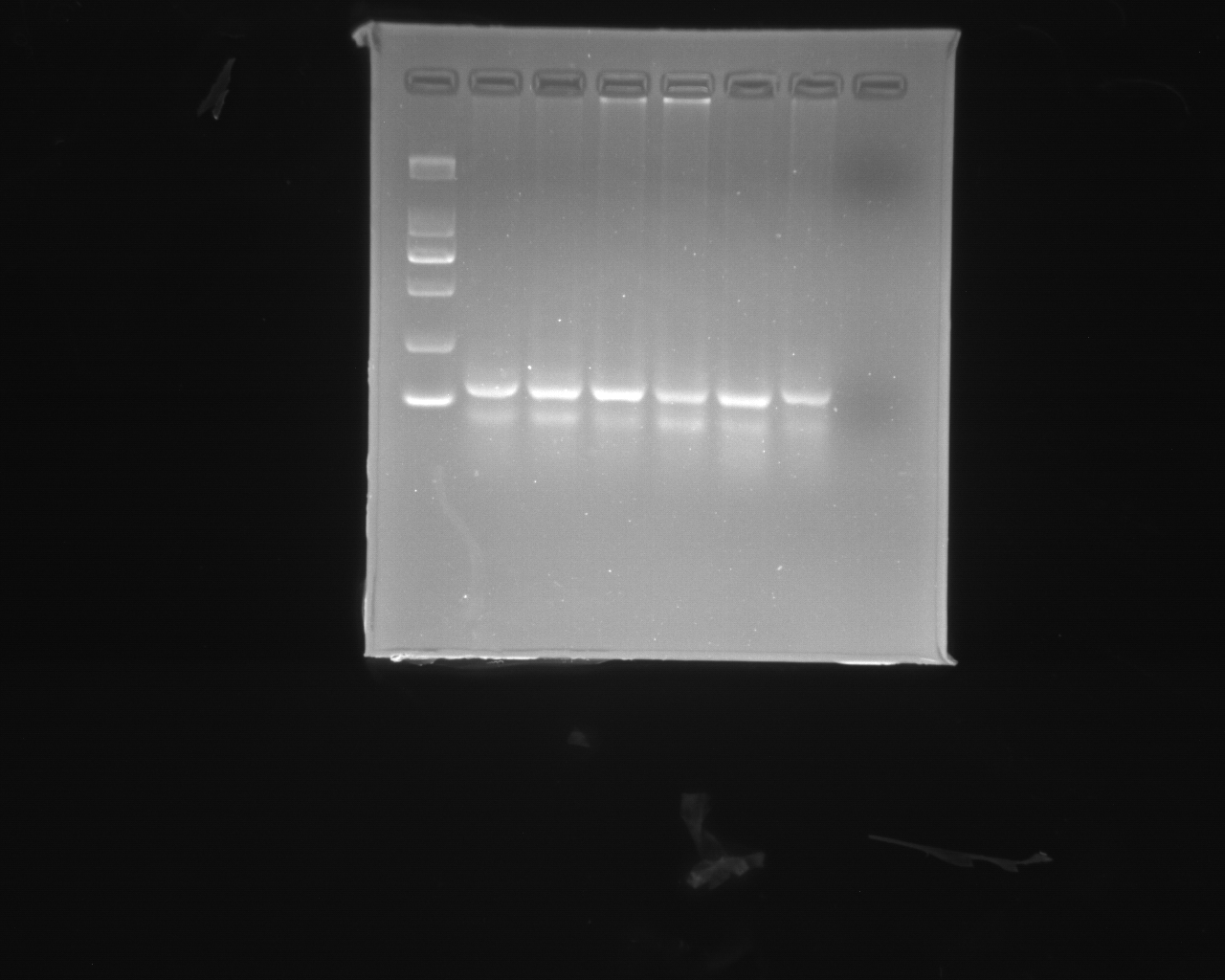

Supplement: S2 File — (ZIP) [file pone.0326317.s002.zip › Supporting Information PCR data/GLUT9/2022-11-6 GLUT9 6.tif]

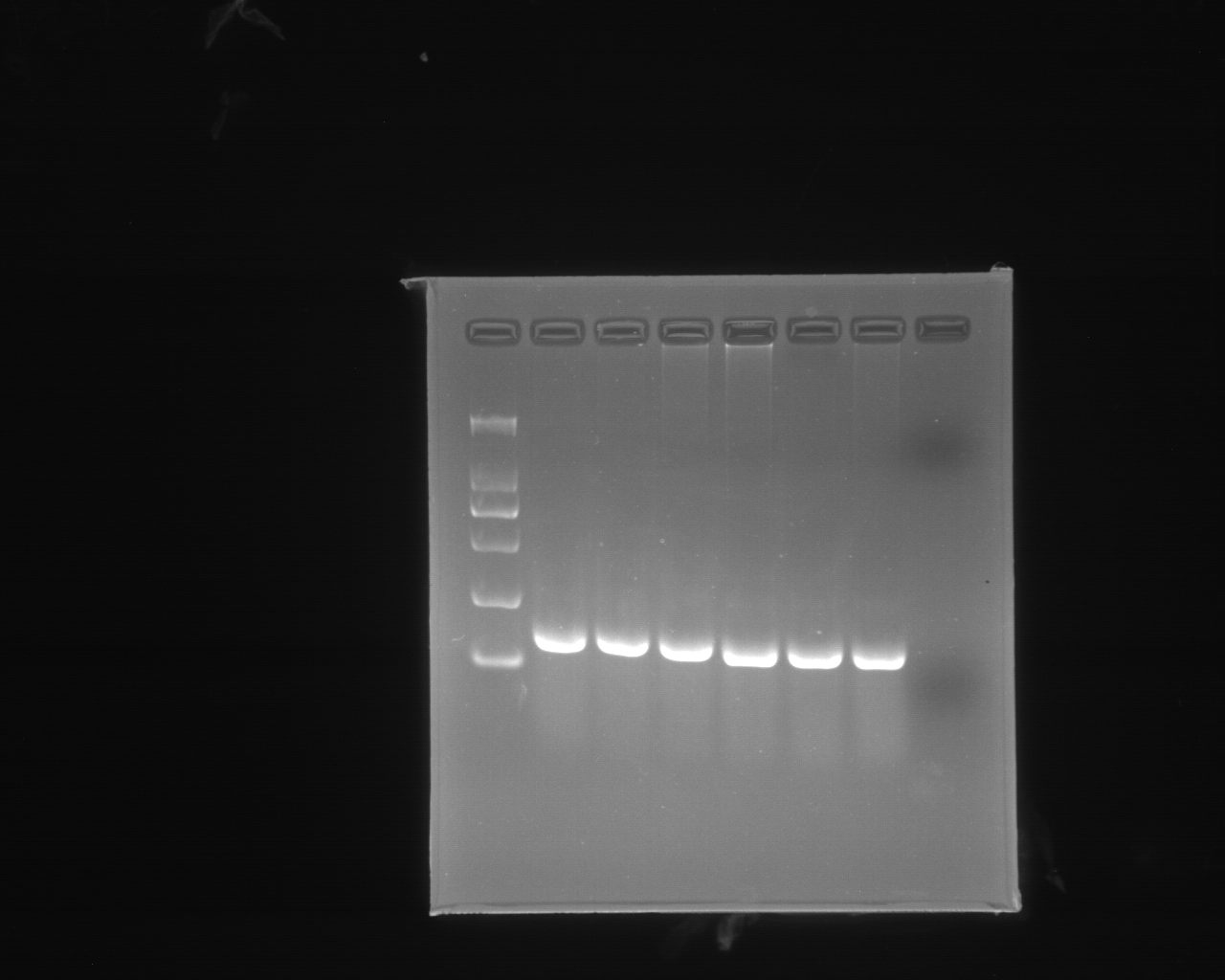

Supplement: S2 File — (ZIP) [file pone.0326317.s002.zip › Supporting Information PCR data/GLUT9/2022-11-7 GAPDH 成功1.tif]

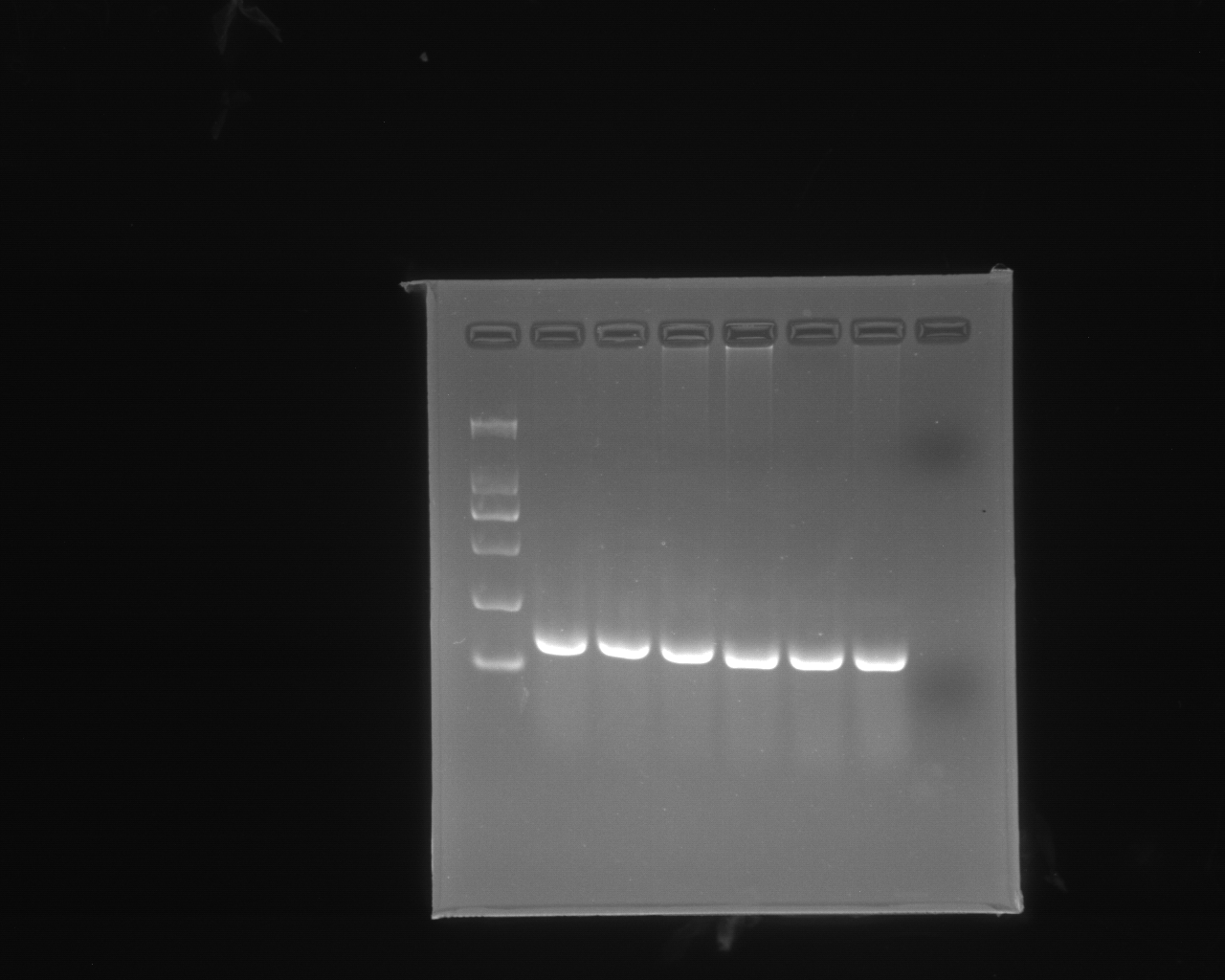

Supplement: S2 File — (ZIP) [file pone.0326317.s002.zip › Supporting Information PCR data/GLUT9/2022-11-7 GAPDH 成功2.tif]

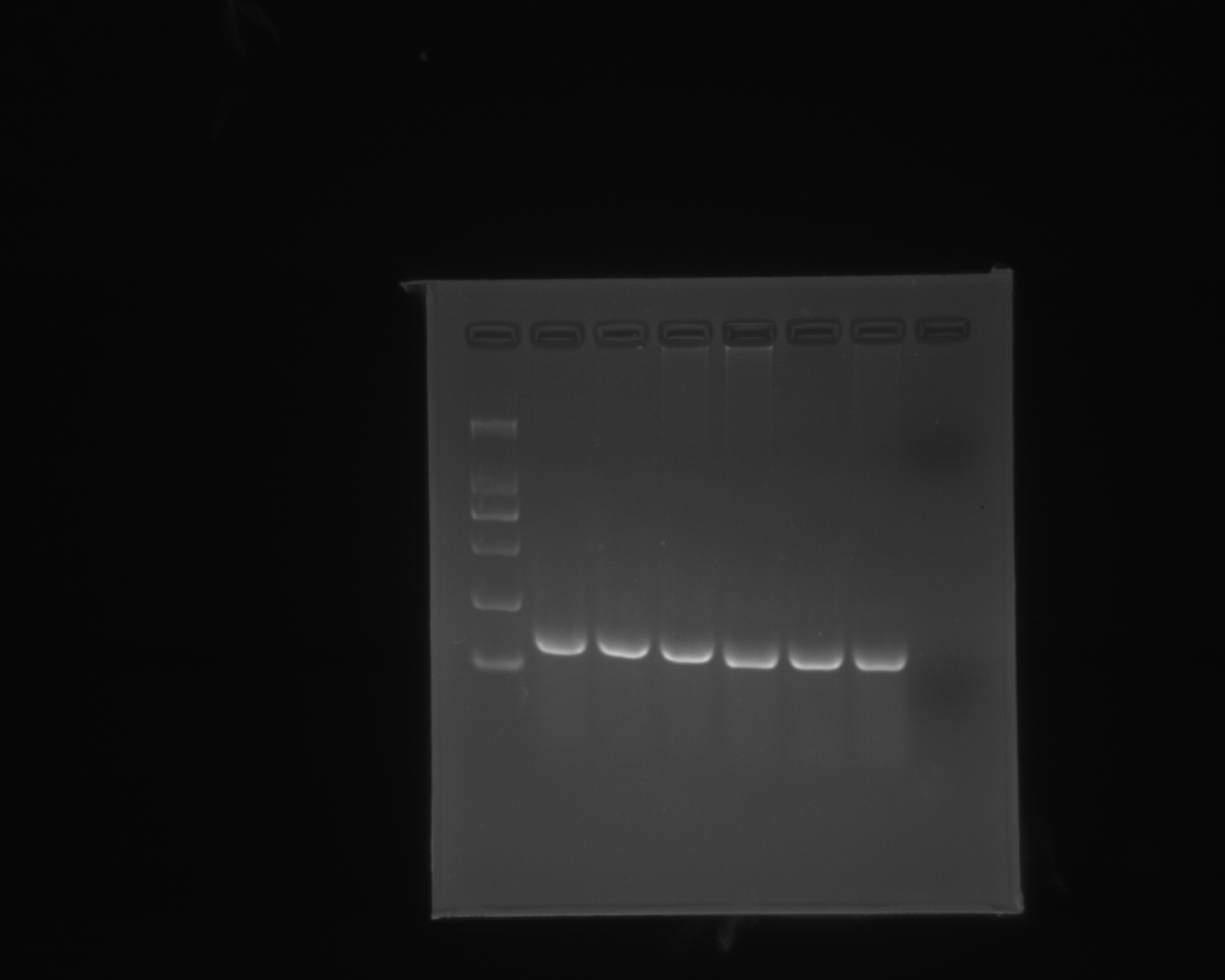

Supplement: S2 File — (ZIP) [file pone.0326317.s002.zip › Supporting Information PCR data/GLUT9/2022-11-7 GAPDH 成功3.tif]

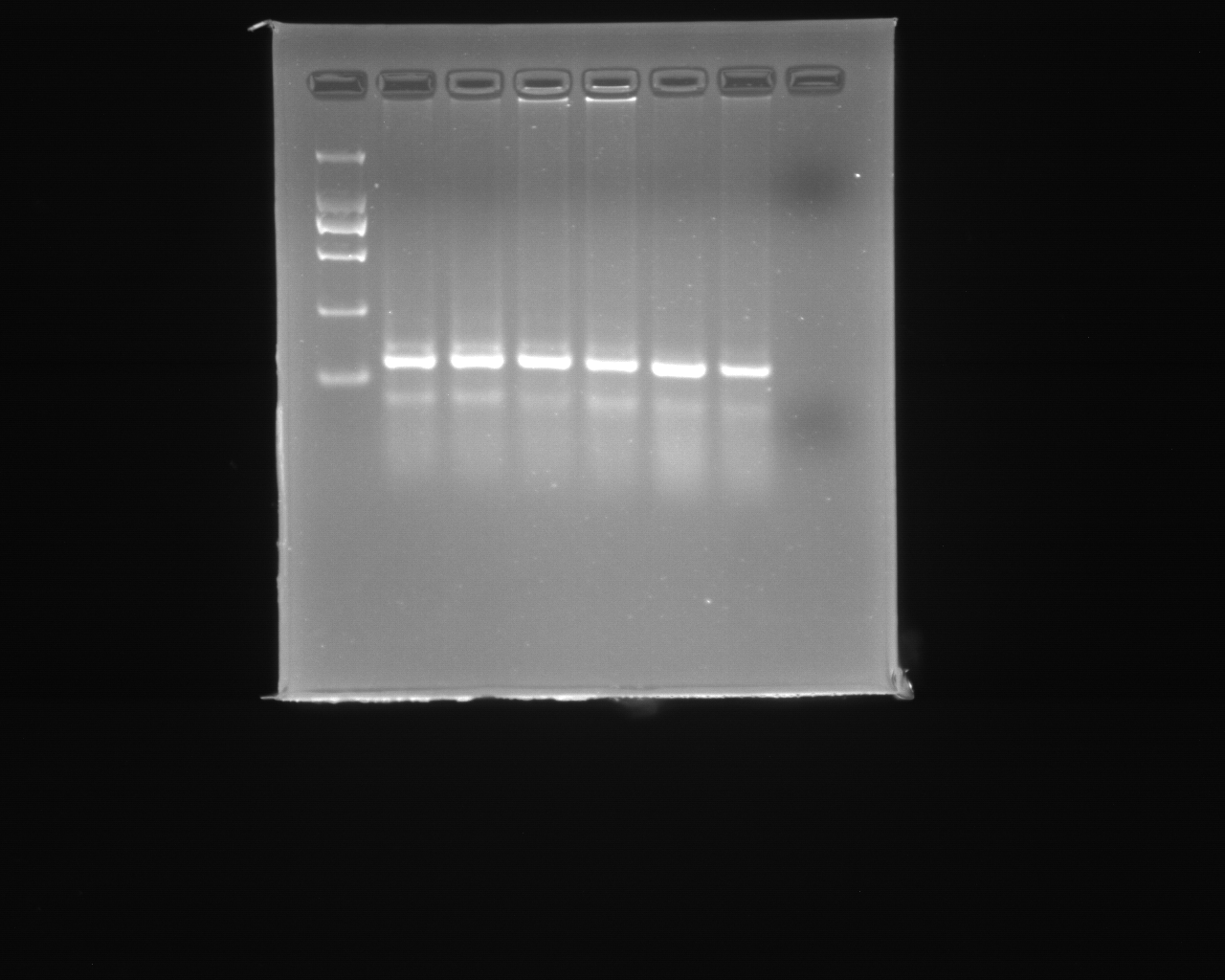

Supplement: S2 File — (ZIP) [file pone.0326317.s002.zip › Supporting Information PCR data/GLUT9/2022-11-7 GLUT9 1.tif]

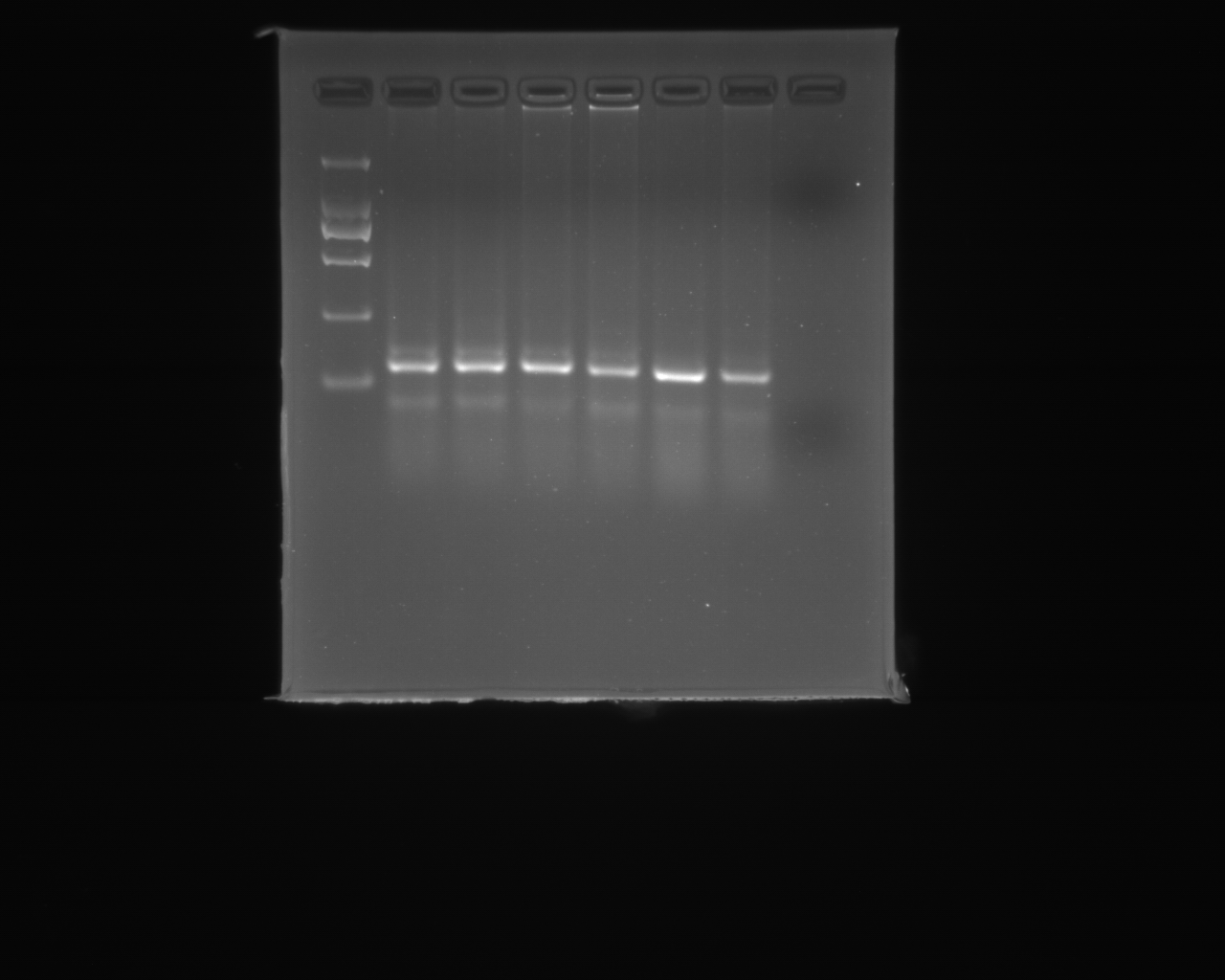

Supplement: S2 File — (ZIP) [file pone.0326317.s002.zip › Supporting Information PCR data/GLUT9/2022-11-7 GLUT9 2.tif]

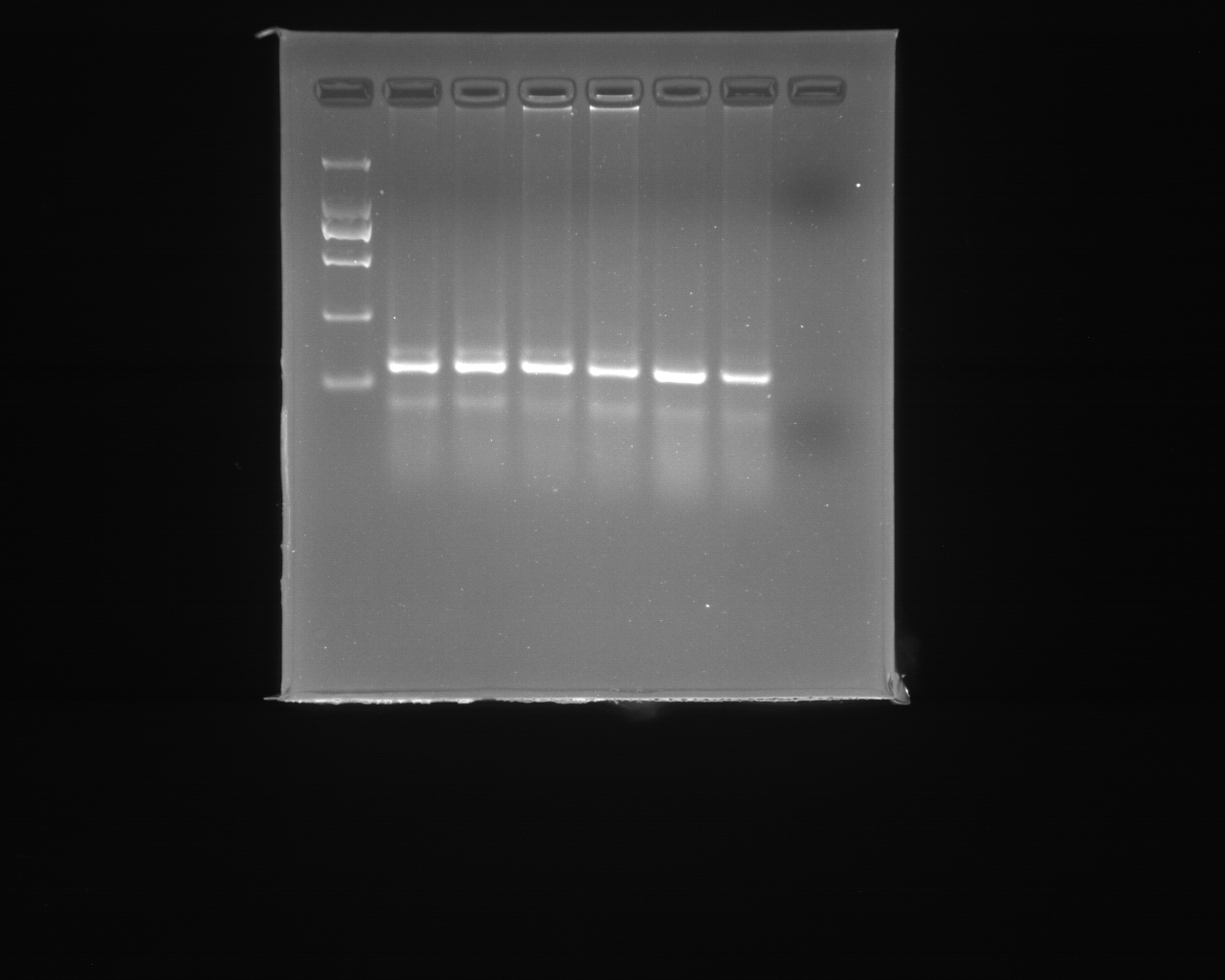

Supplement: S2 File — (ZIP) [file pone.0326317.s002.zip › Supporting Information PCR data/GLUT9/2022-11-7 GLUT9 3.tif]

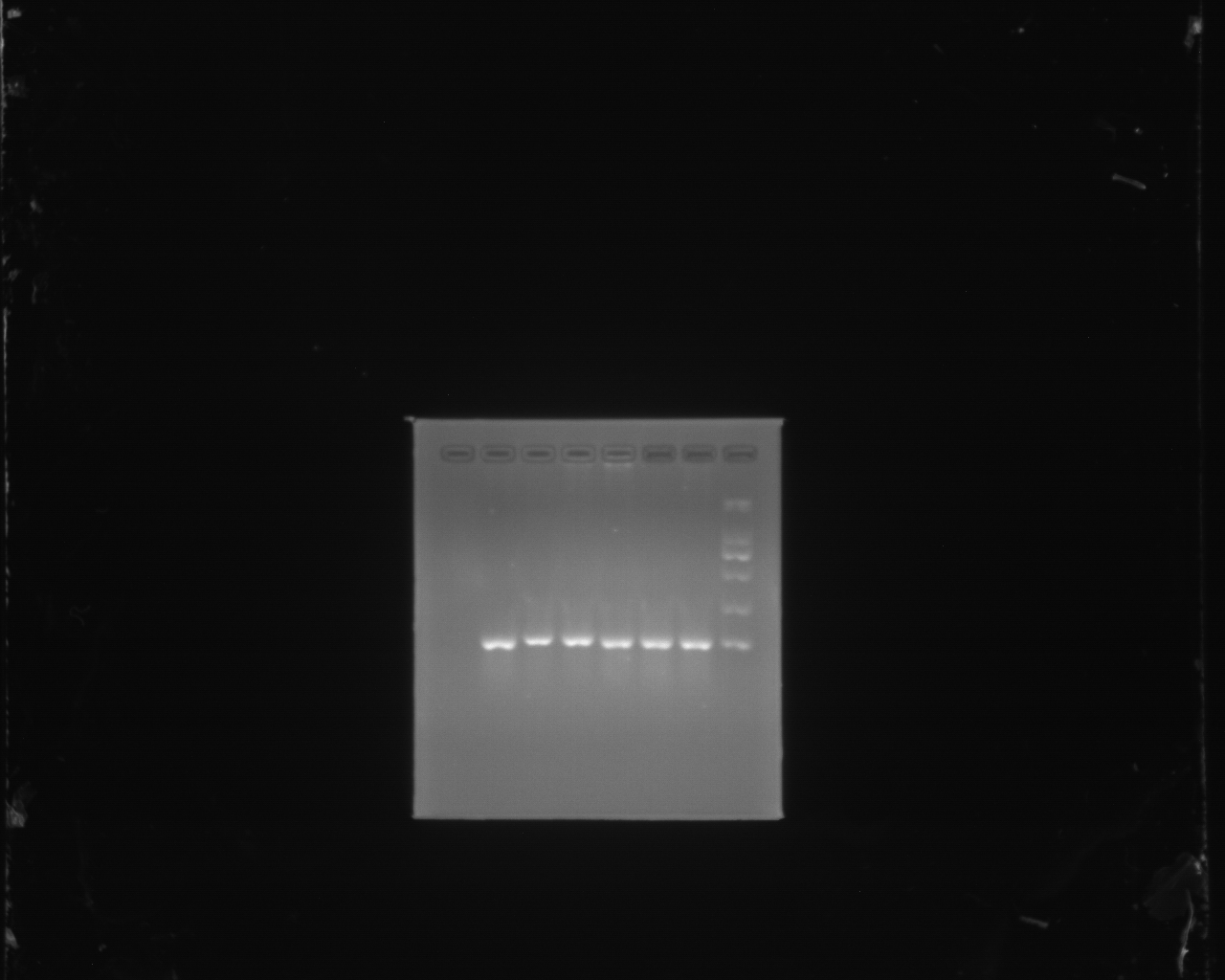

Supplement: S2 File — (ZIP) [file pone.0326317.s002.zip › Supporting Information PCR data/OAT1/2022-10-28 OAT1最新 1.tif]

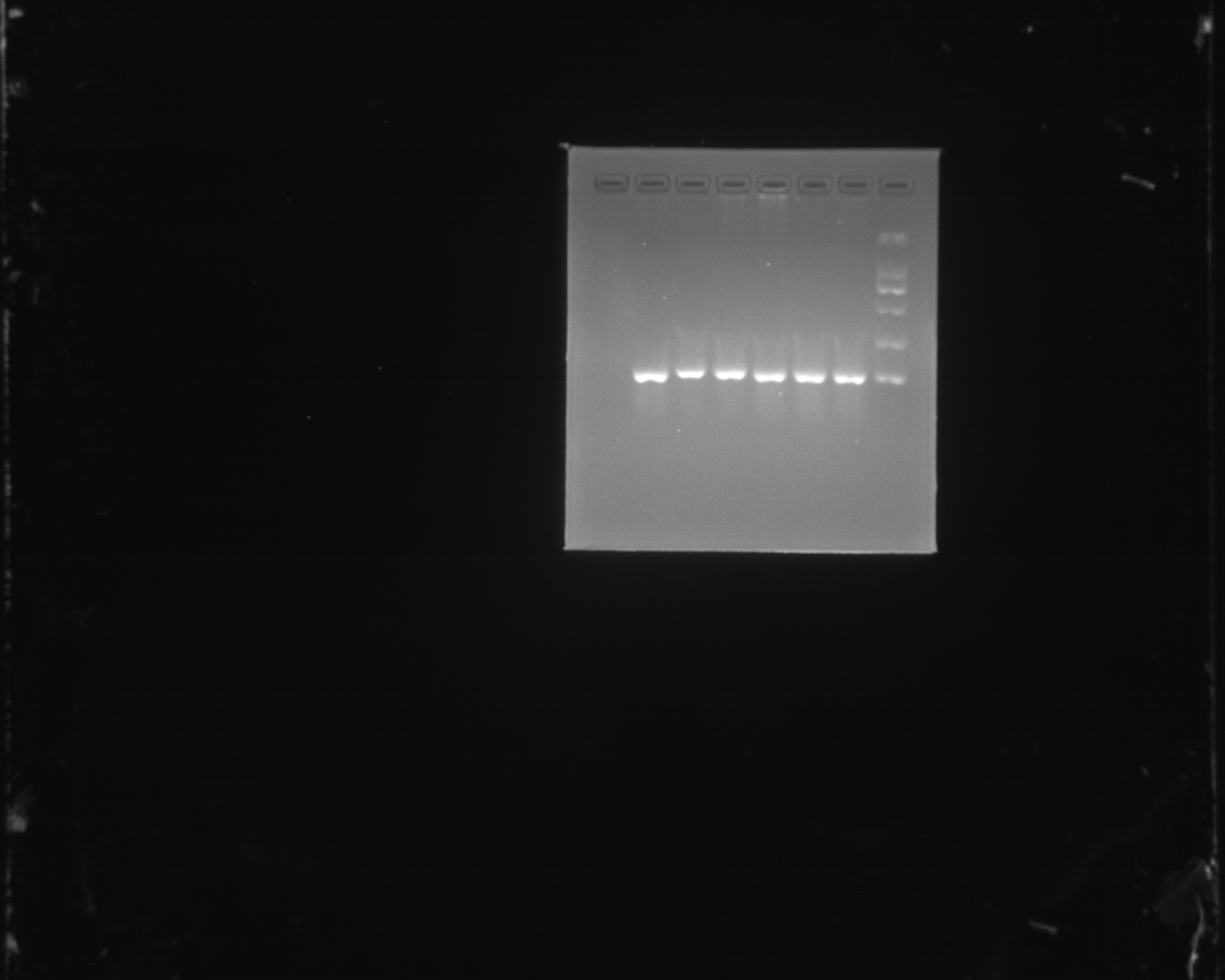

Supplement: S2 File — (ZIP) [file pone.0326317.s002.zip › Supporting Information PCR data/OAT1/2022-10-28 OAT1最新 6.tif]

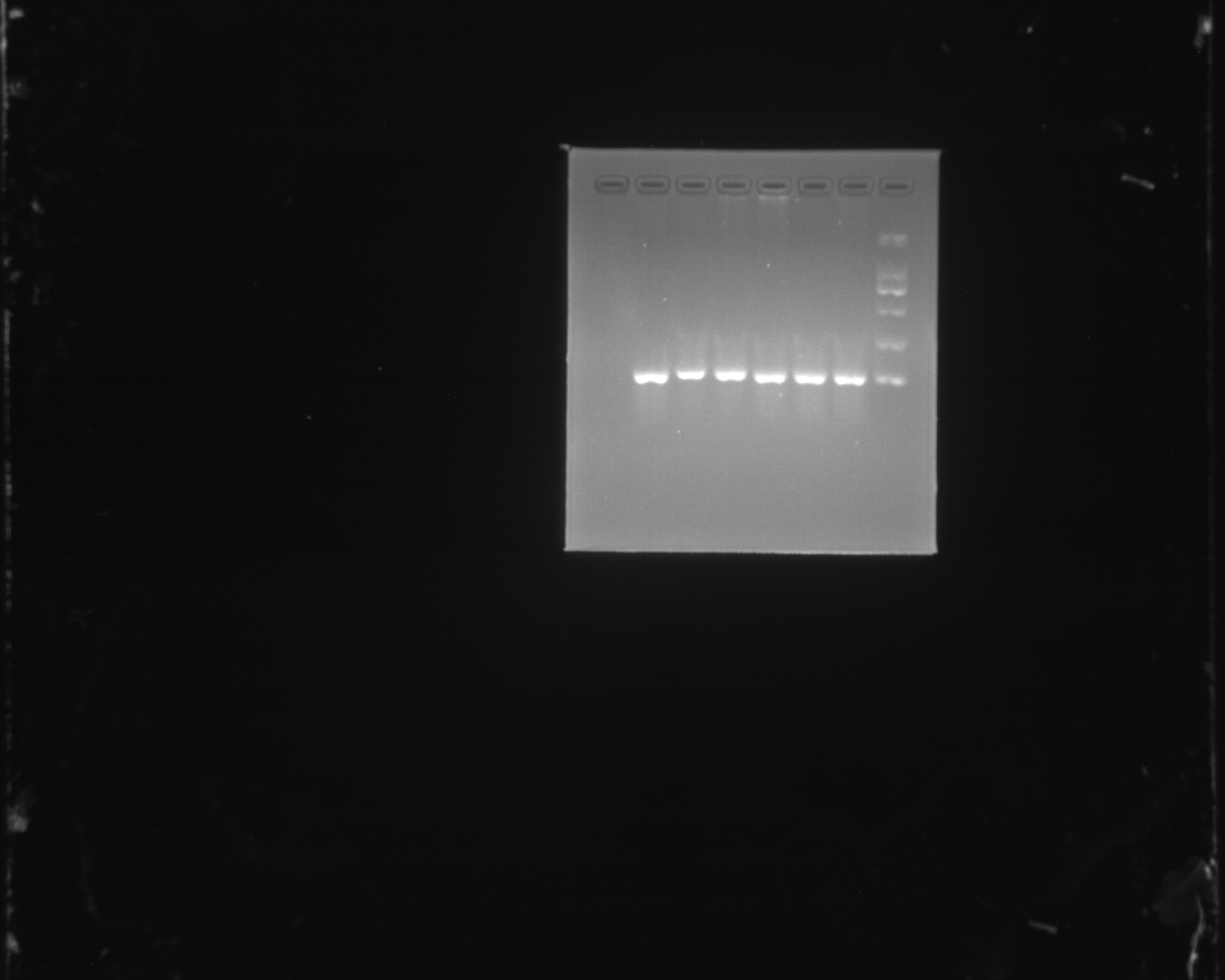

Supplement: S2 File — (ZIP) [file pone.0326317.s002.zip › Supporting Information PCR data/OAT1/2022-10-28 OAT1最新 7.tif]

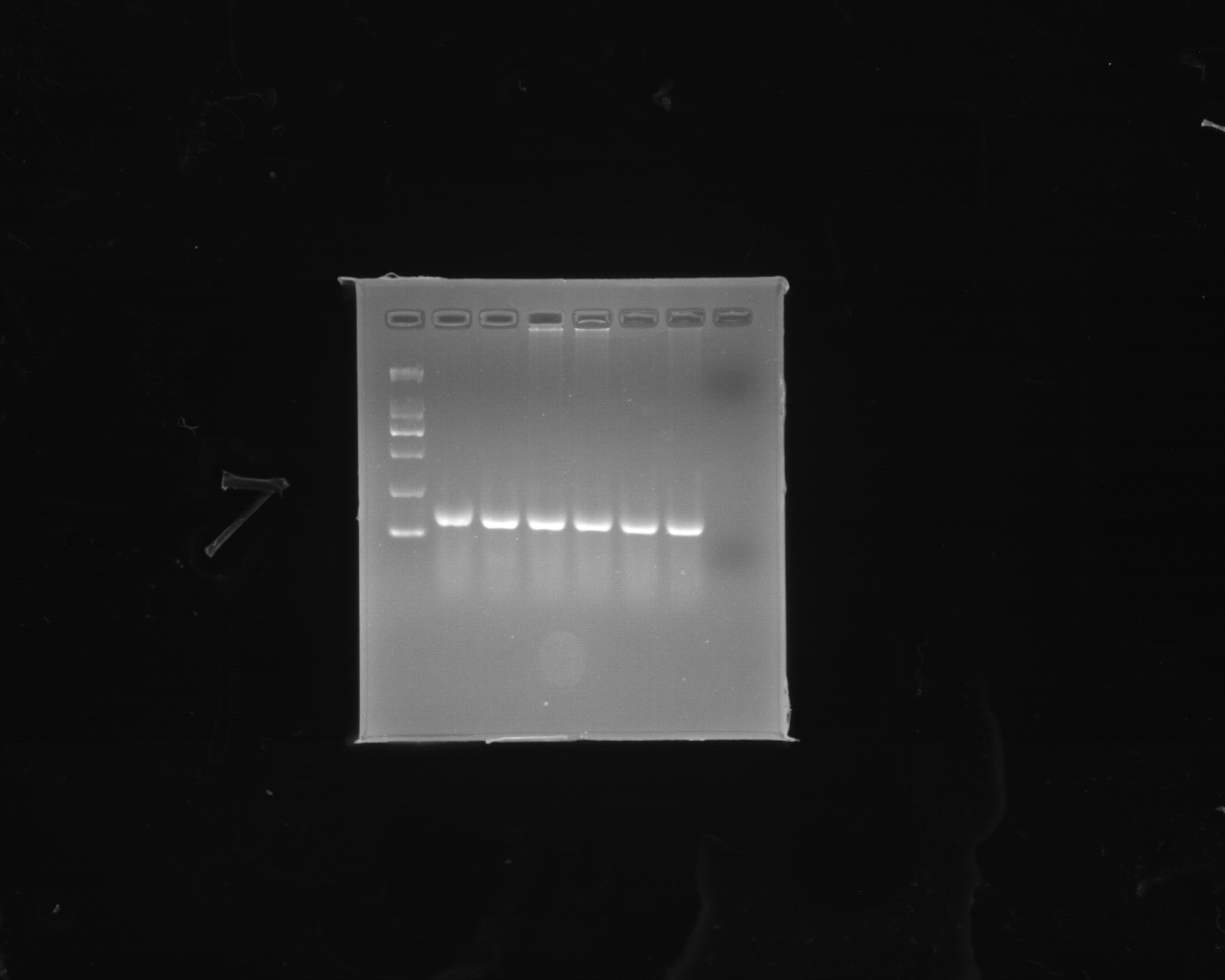

Supplement: S2 File — (ZIP) [file pone.0326317.s002.zip › Supporting Information PCR data/OAT1/2022-11-1 GAPDH调 2.tif]

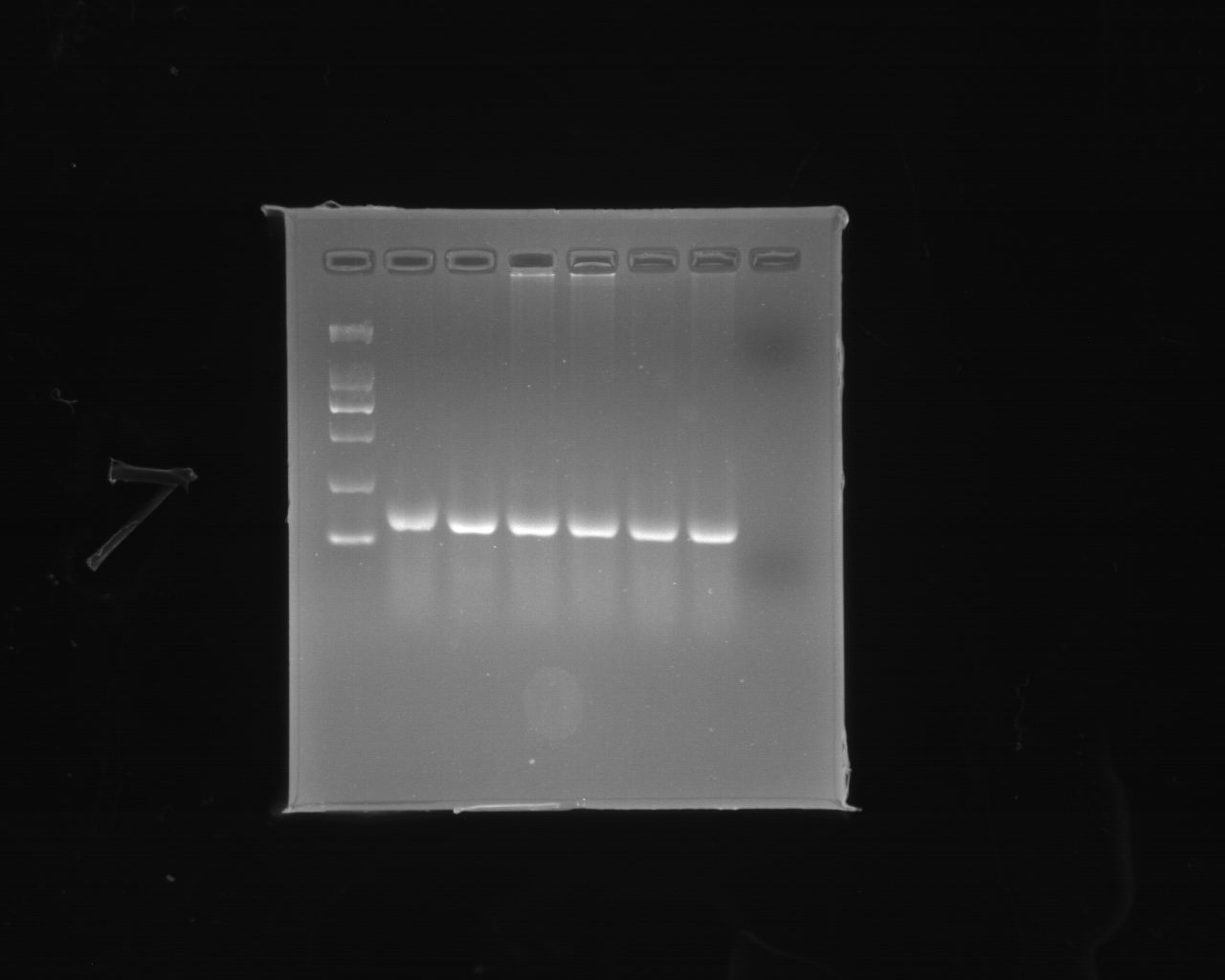

Supplement: S2 File — (ZIP) [file pone.0326317.s002.zip › Supporting Information PCR data/OAT1/2022-11-1 GAPDH调 5.tif]

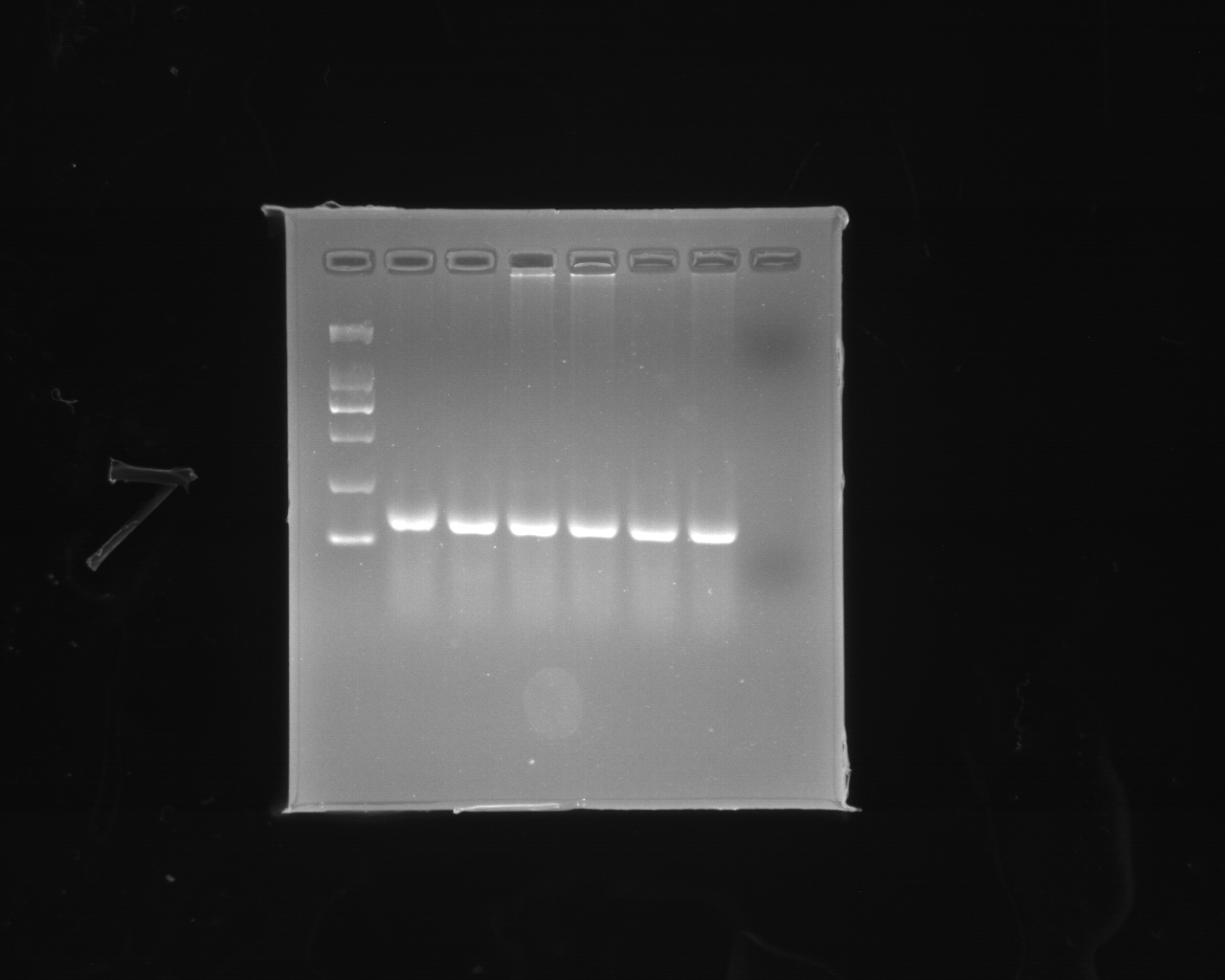

Supplement: S2 File — (ZIP) [file pone.0326317.s002.zip › Supporting Information PCR data/OAT1/2022-11-1 GAPDH调 7.tif]

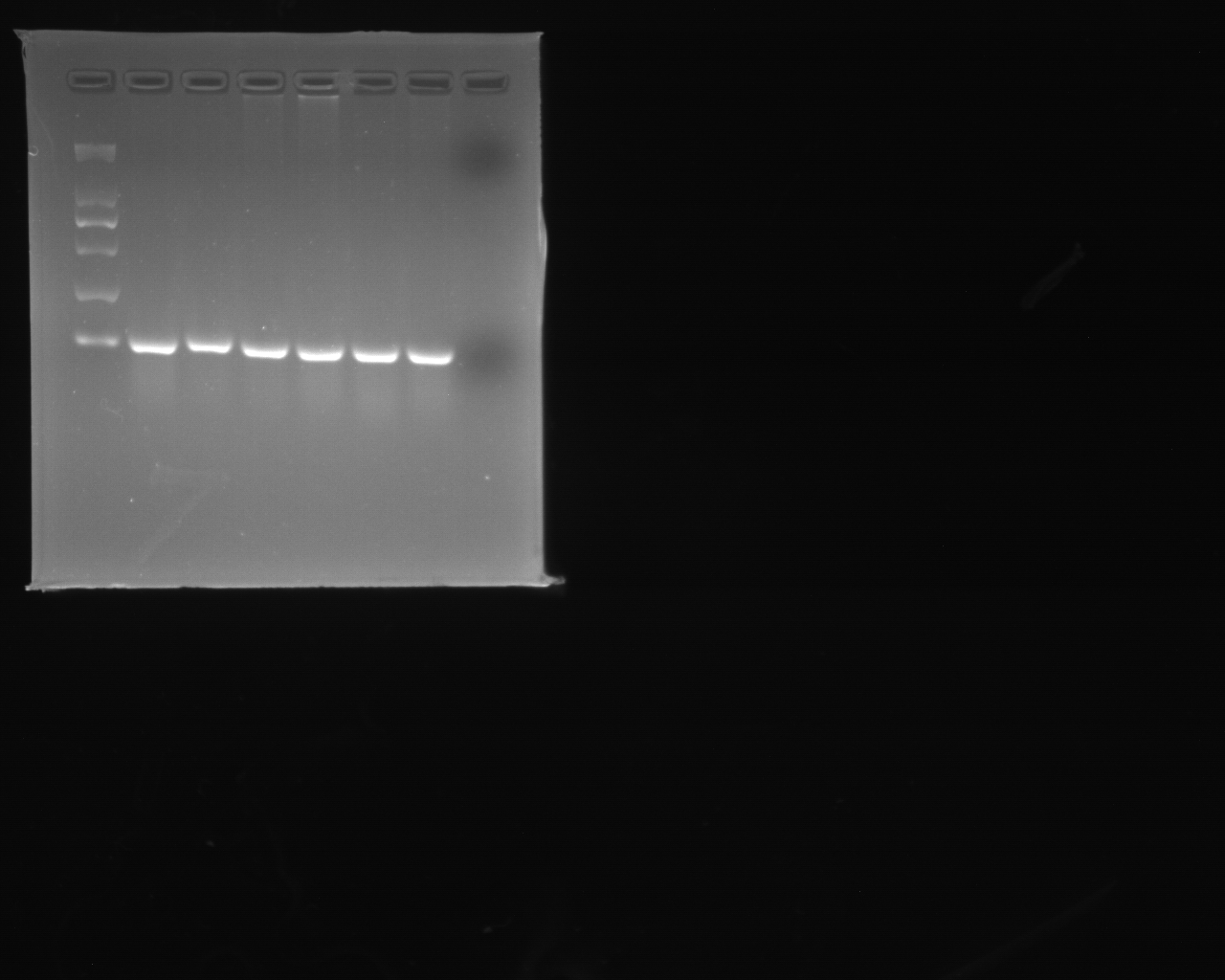

Supplement: S2 File — (ZIP) [file pone.0326317.s002.zip › Supporting Information PCR data/OAT1/2022-11-1 OAT1最新2.tif]

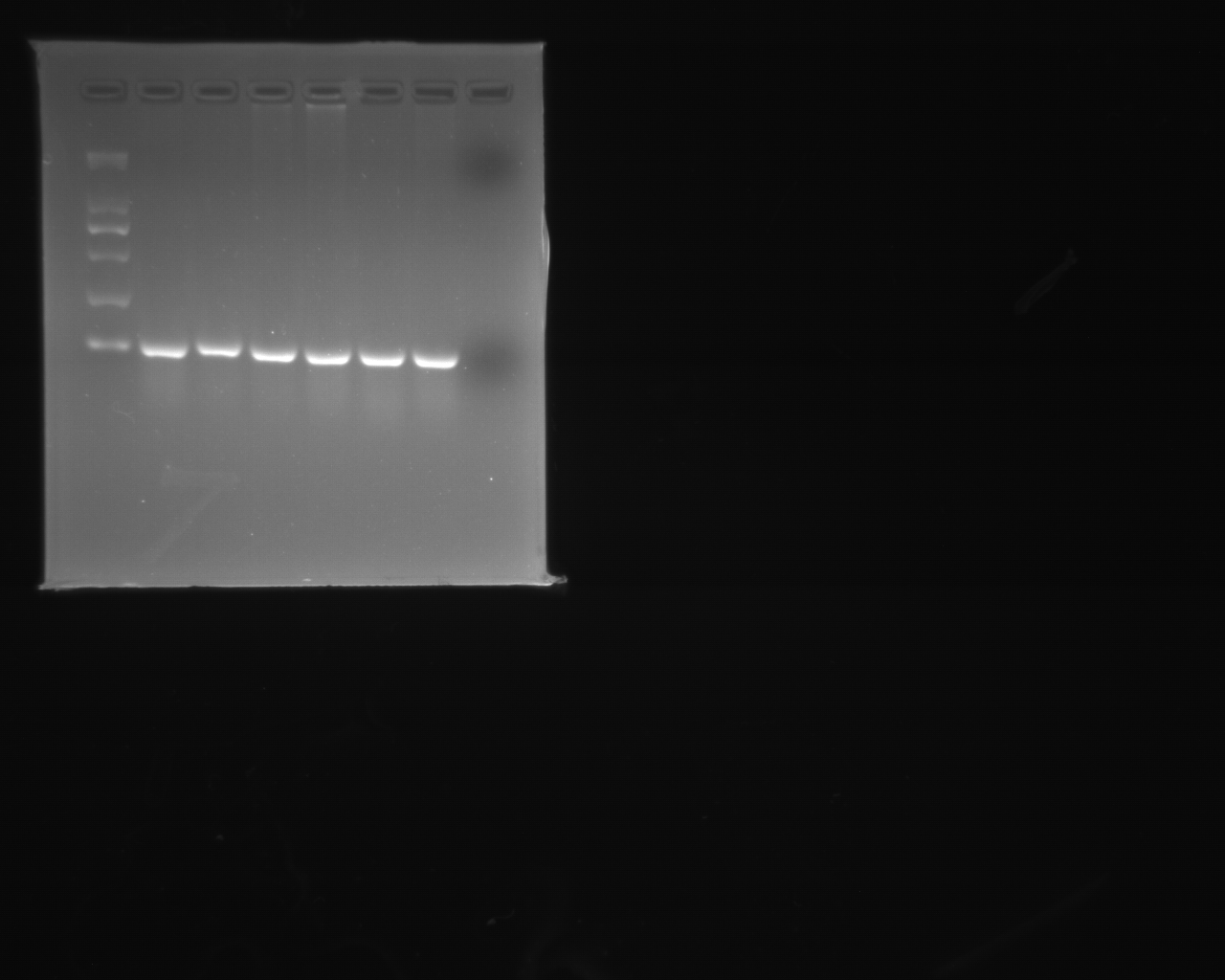

Supplement: S2 File — (ZIP) [file pone.0326317.s002.zip › Supporting Information PCR data/OAT1/2022-11-1 OAT1最新5.tif]

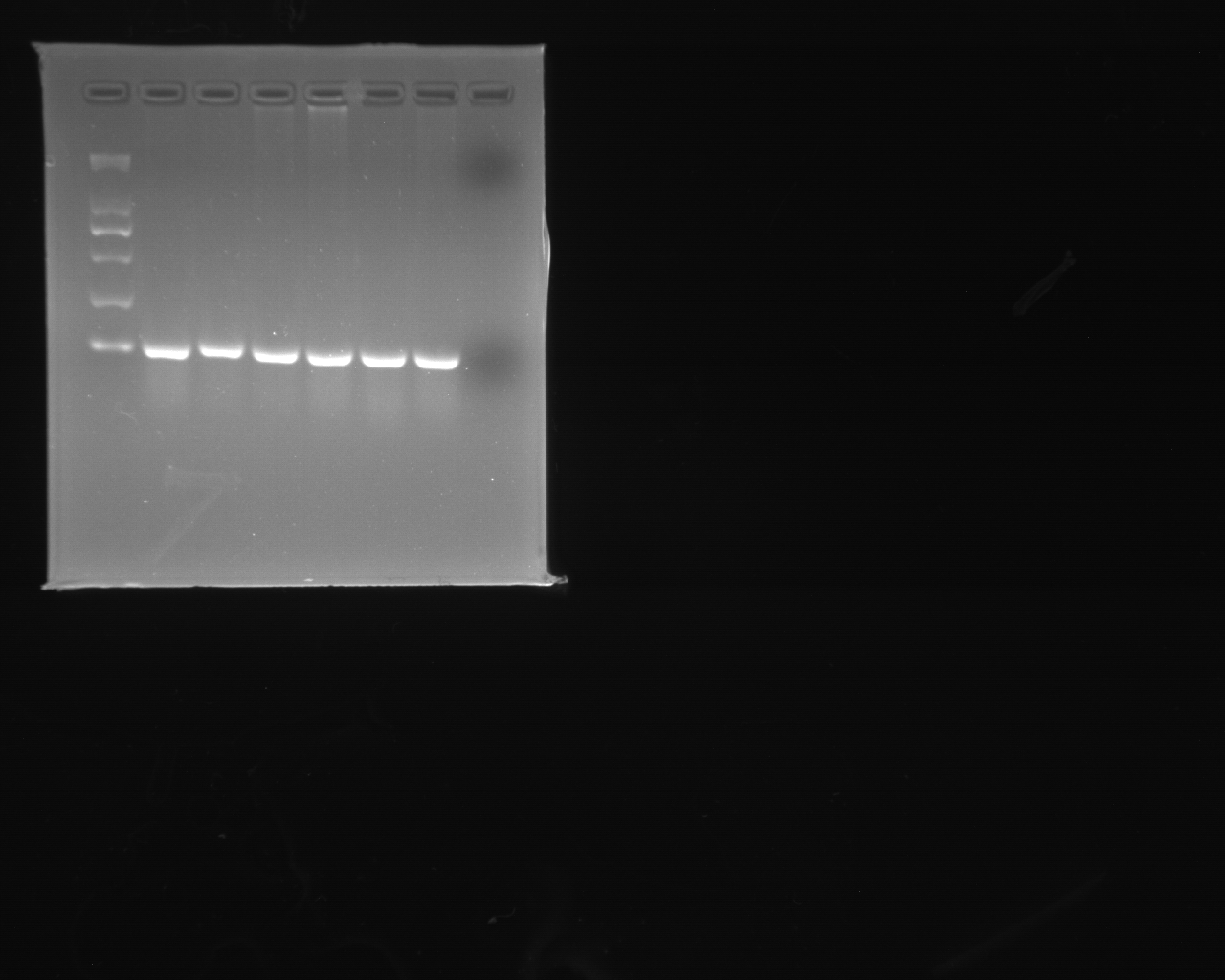

Supplement: S2 File — (ZIP) [file pone.0326317.s002.zip › Supporting Information PCR data/OAT1/2022-11-1 OAT1最新7.tif]

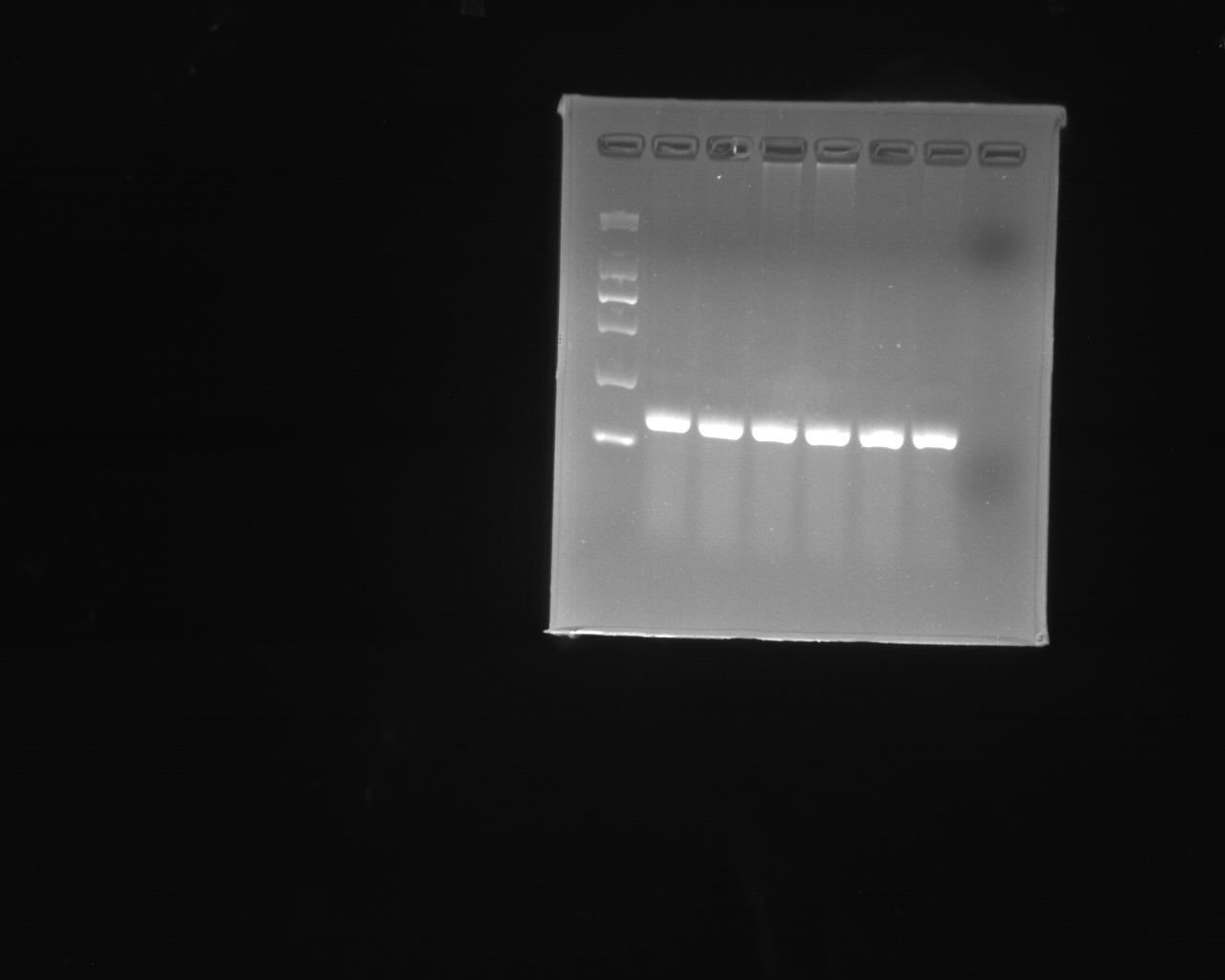

Supplement: S2 File — (ZIP) [file pone.0326317.s002.zip › Supporting Information PCR data/OAT1/2022-11-14 GAPDH 1.tif]

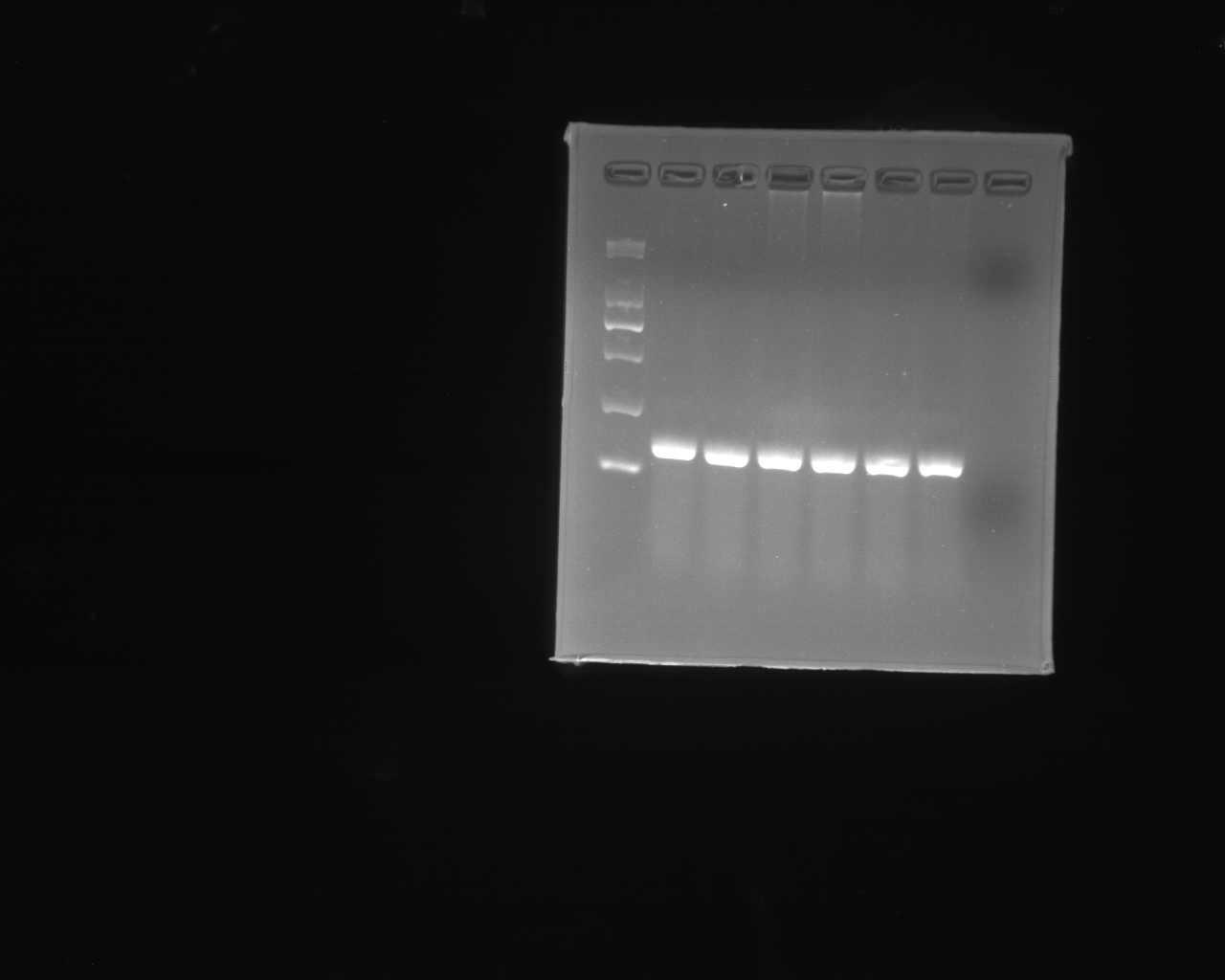

Supplement: S2 File — (ZIP) [file pone.0326317.s002.zip › Supporting Information PCR data/OAT1/2022-11-14 GAPDH 2.tif]

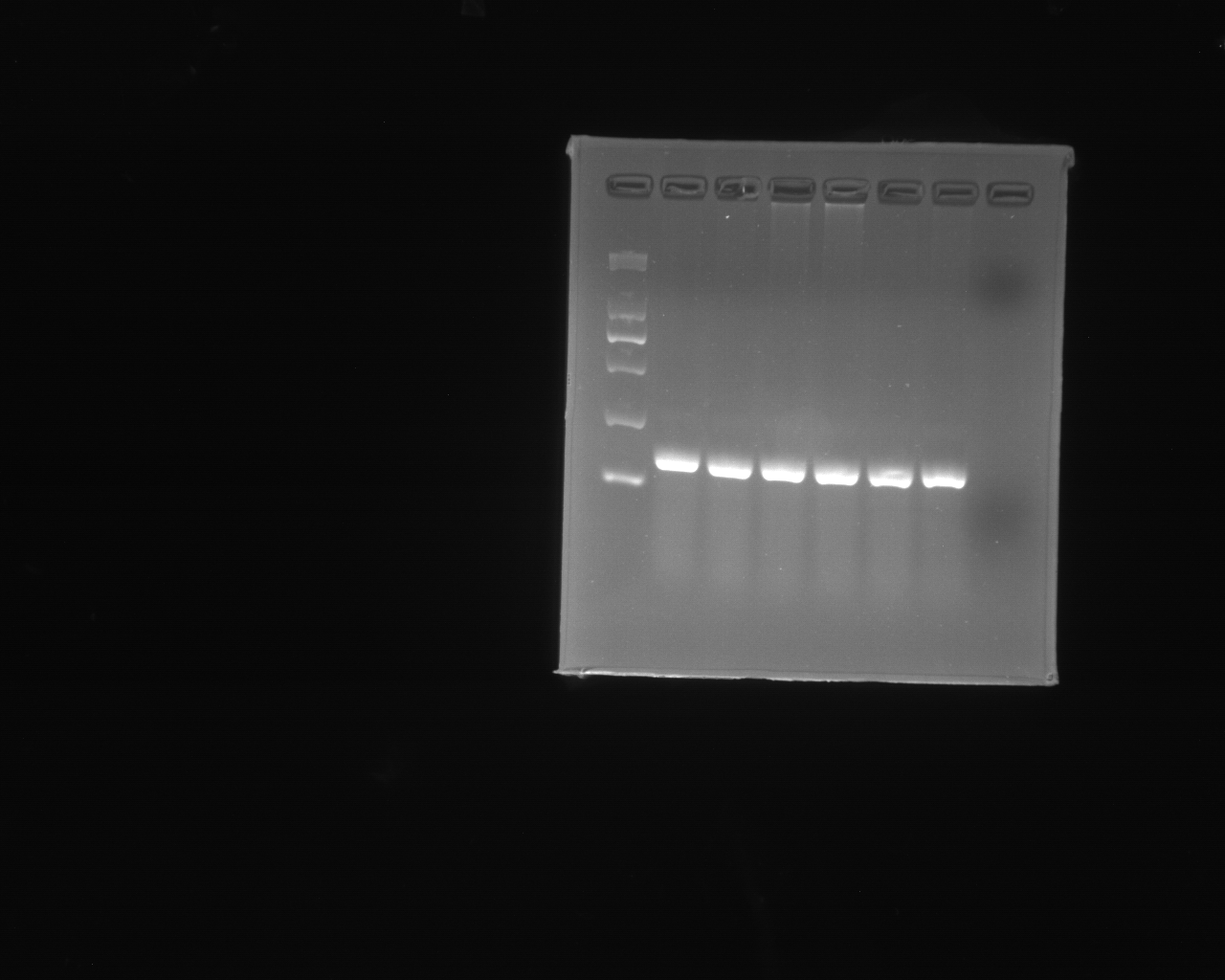

Supplement: S2 File — (ZIP) [file pone.0326317.s002.zip › Supporting Information PCR data/OAT1/2022-11-14 GAPDH 3.tif]

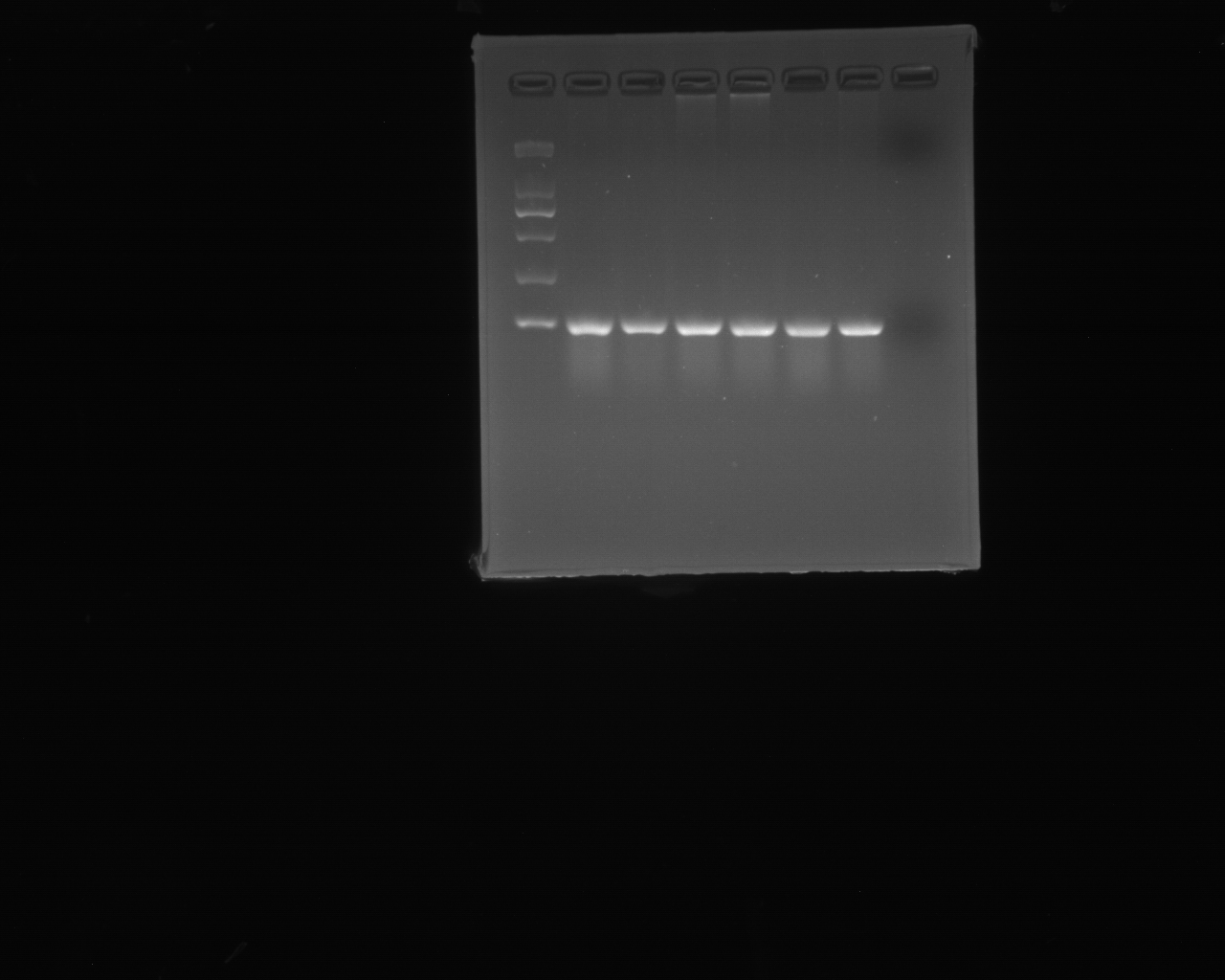

Supplement: S2 File — (ZIP) [file pone.0326317.s002.zip › Supporting Information PCR data/OAT1/2022-11-14 OAT1 2-1 cheng.tif]

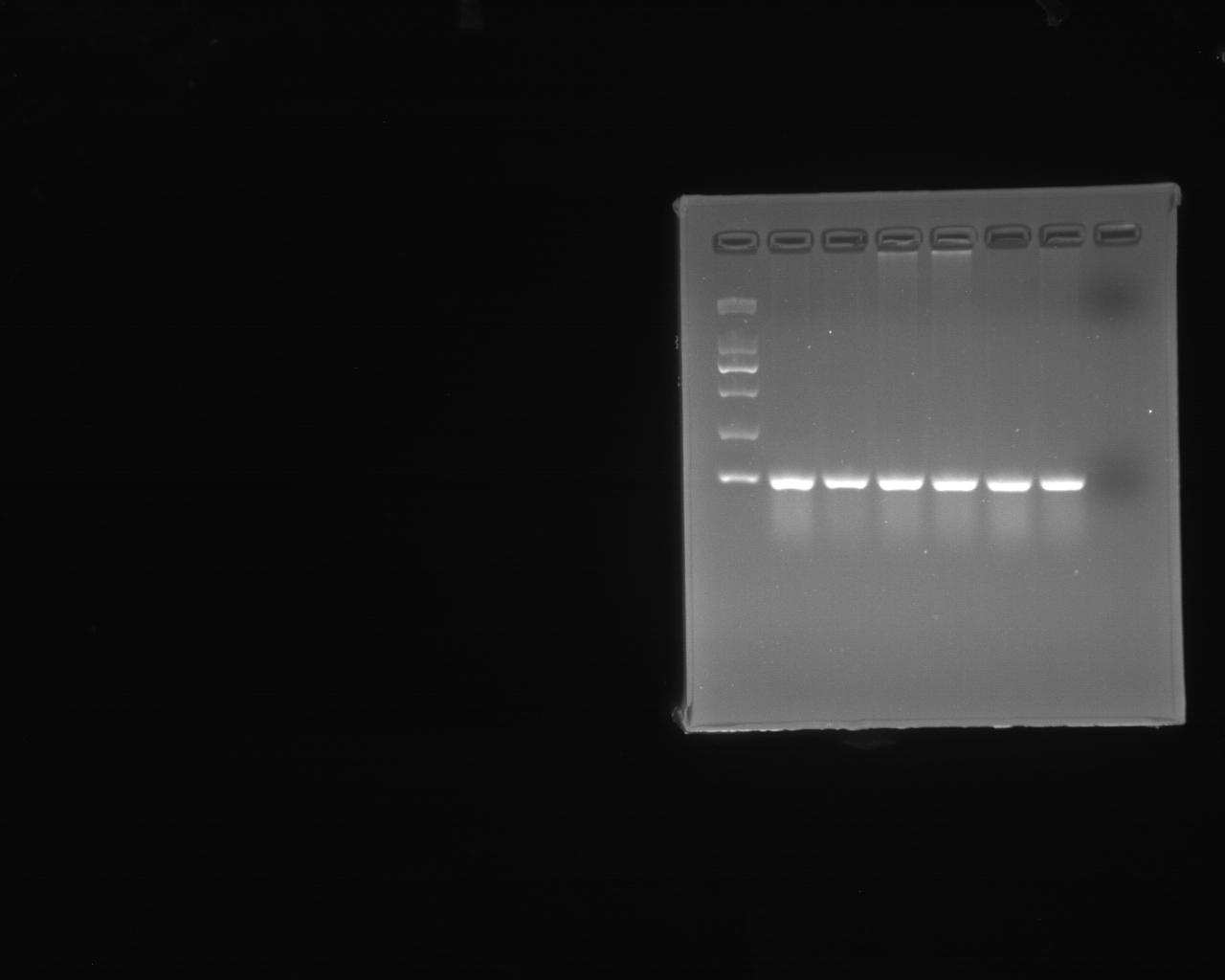

Supplement: S2 File — (ZIP) [file pone.0326317.s002.zip › Supporting Information PCR data/OAT1/2022-11-14 OAT1 2-3 cheng.tif]

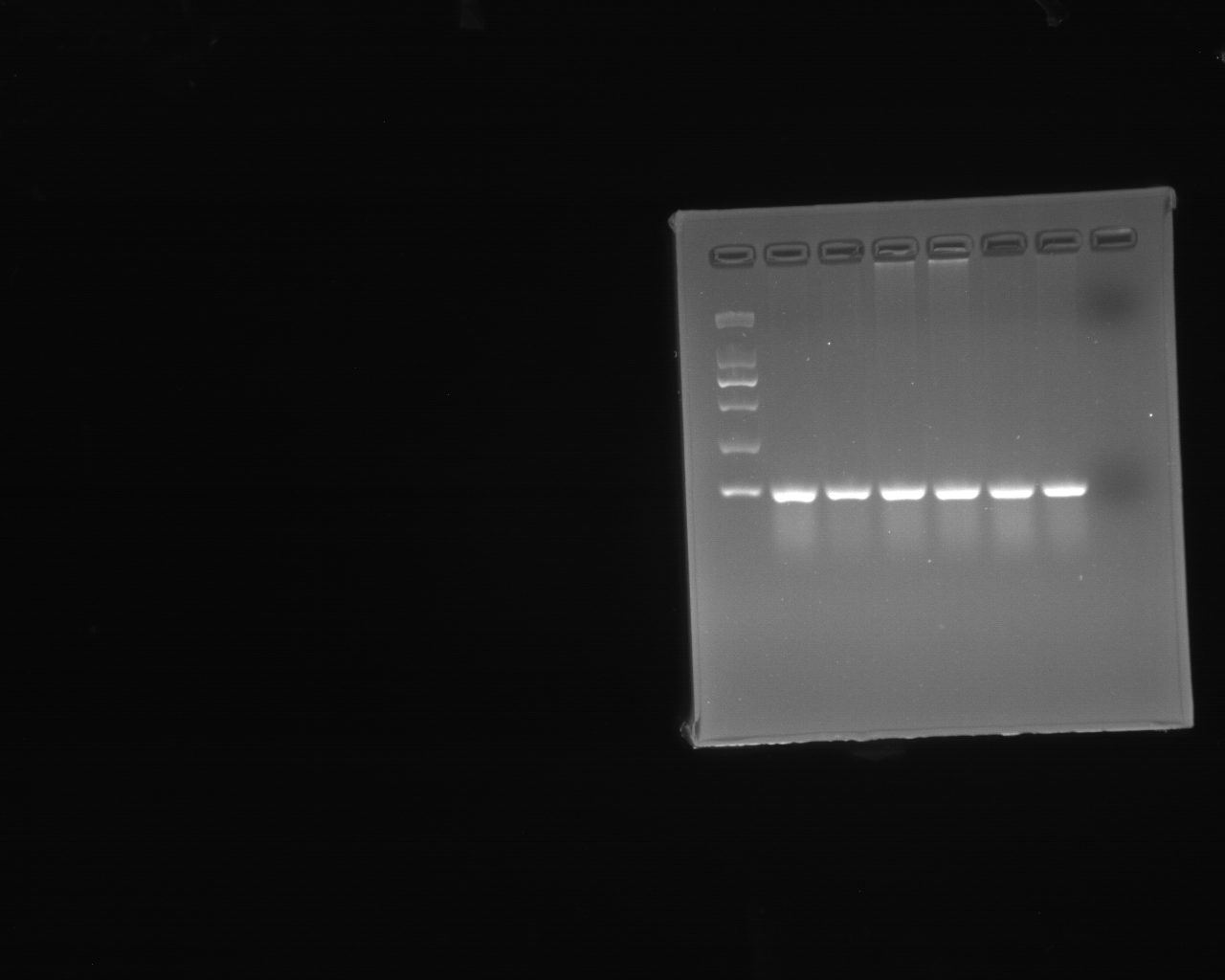

Supplement: S2 File — (ZIP) [file pone.0326317.s002.zip › Supporting Information PCR data/OAT1/2022-11-14 OAT1 2-5 cheng.tif]

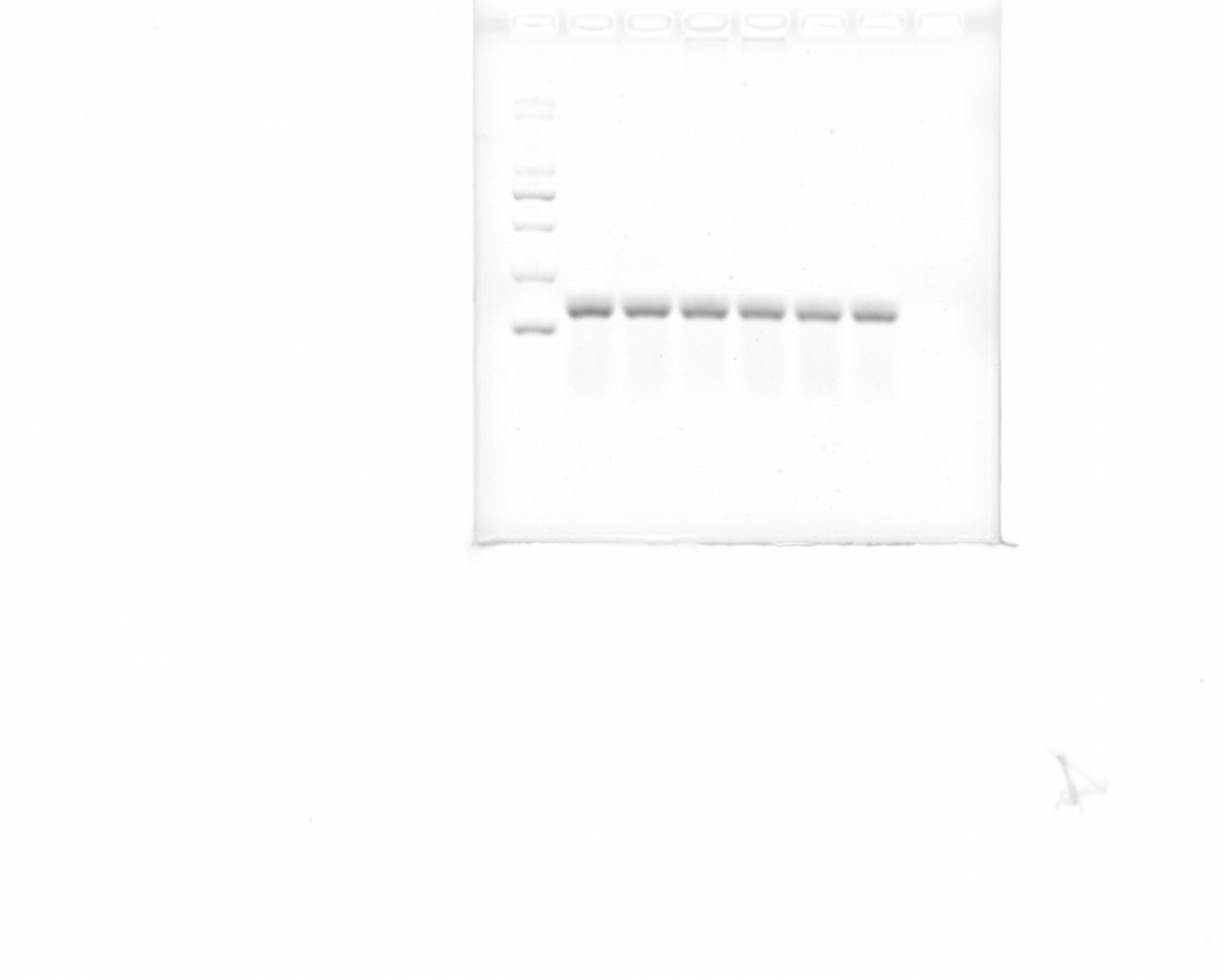

Supplement: S2 File — (ZIP) [file pone.0326317.s002.zip › Supporting Information PCR data/OAT3/2022-11-12 GAPDH 3.tif]

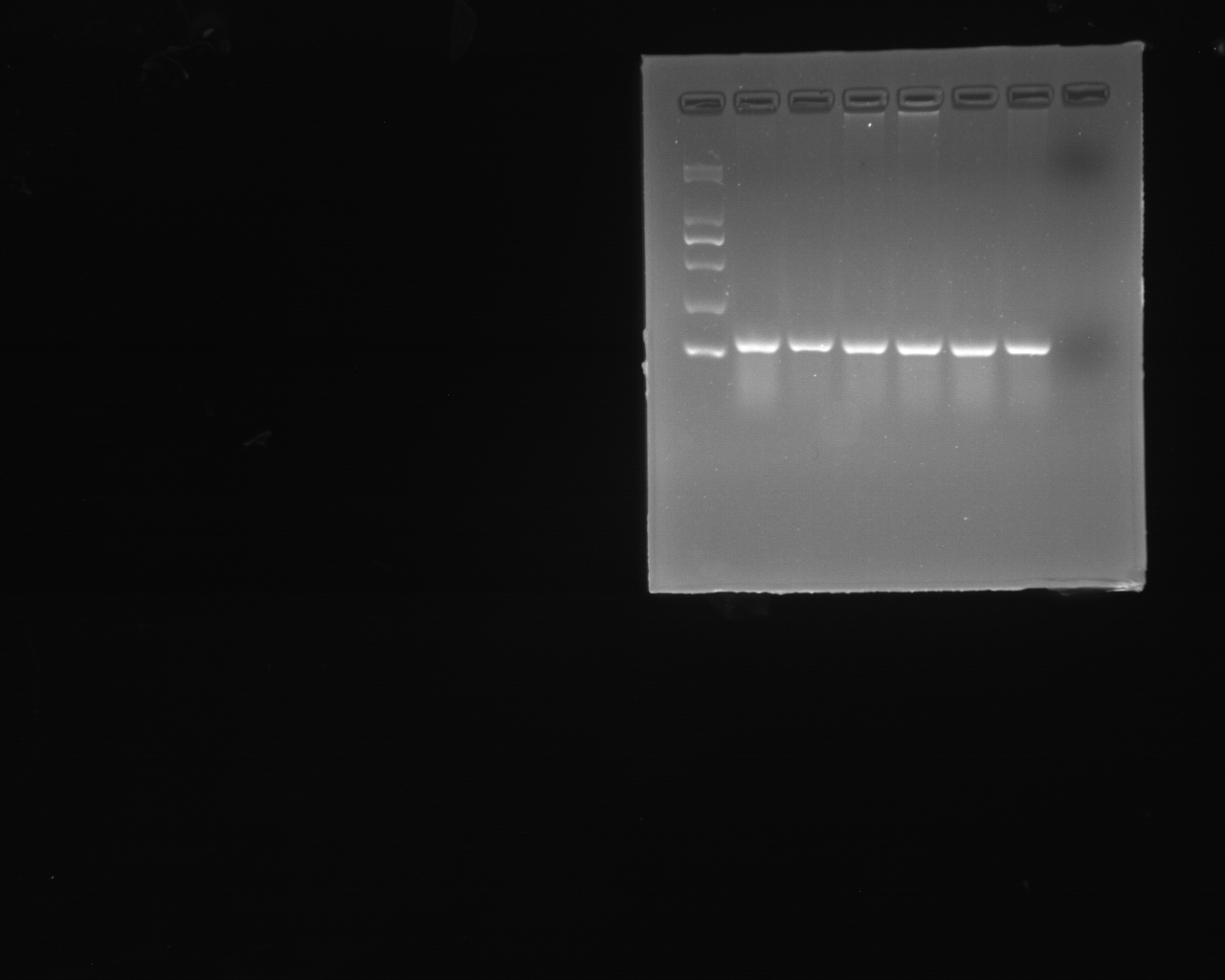

Supplement: S2 File — (ZIP) [file pone.0326317.s002.zip › Supporting Information PCR data/OAT3/2022-11-13 OAT3 1 cheng.tif]

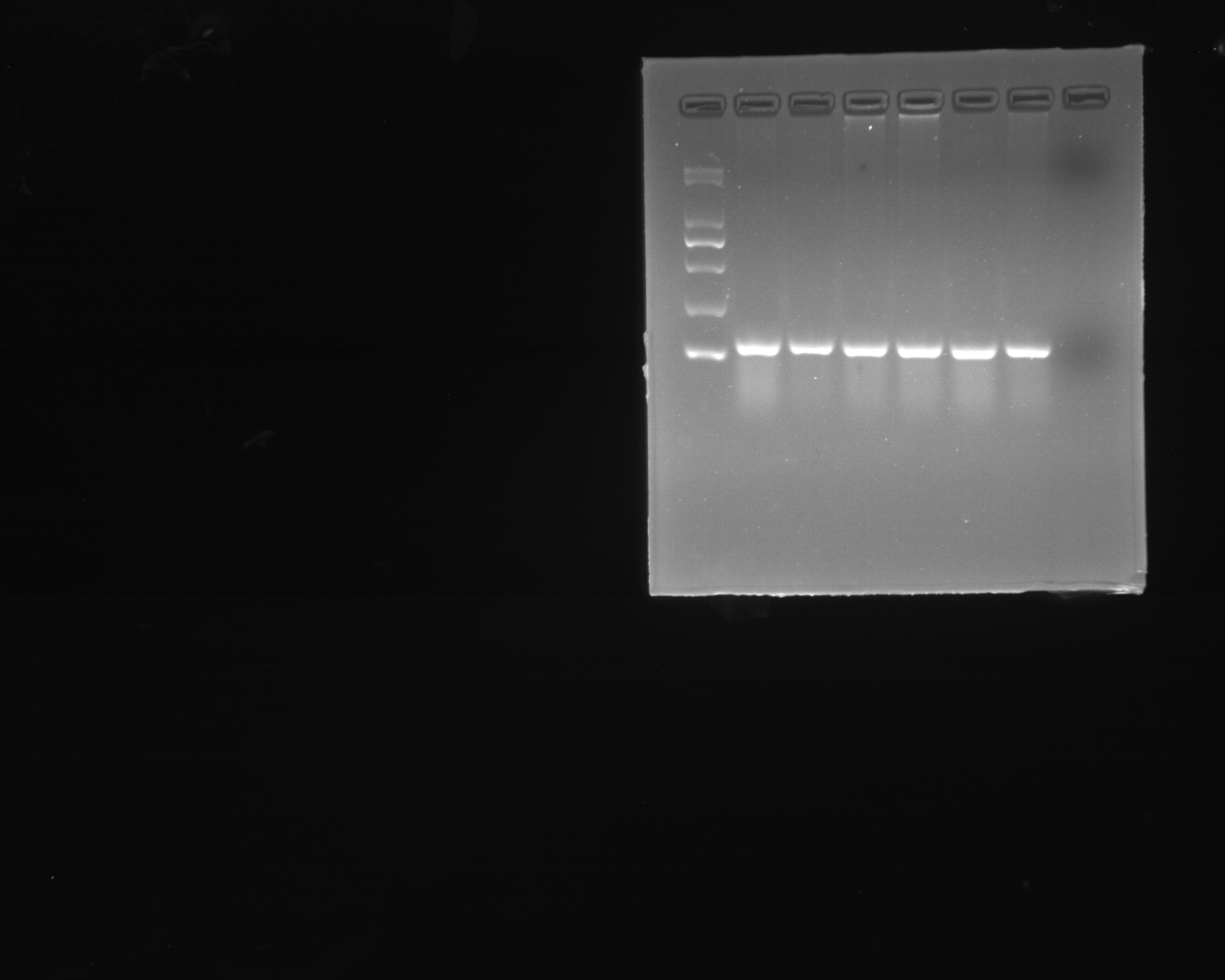

Supplement: S2 File — (ZIP) [file pone.0326317.s002.zip › Supporting Information PCR data/OAT3/2022-11-13 OAT3 2 cheng.tif]

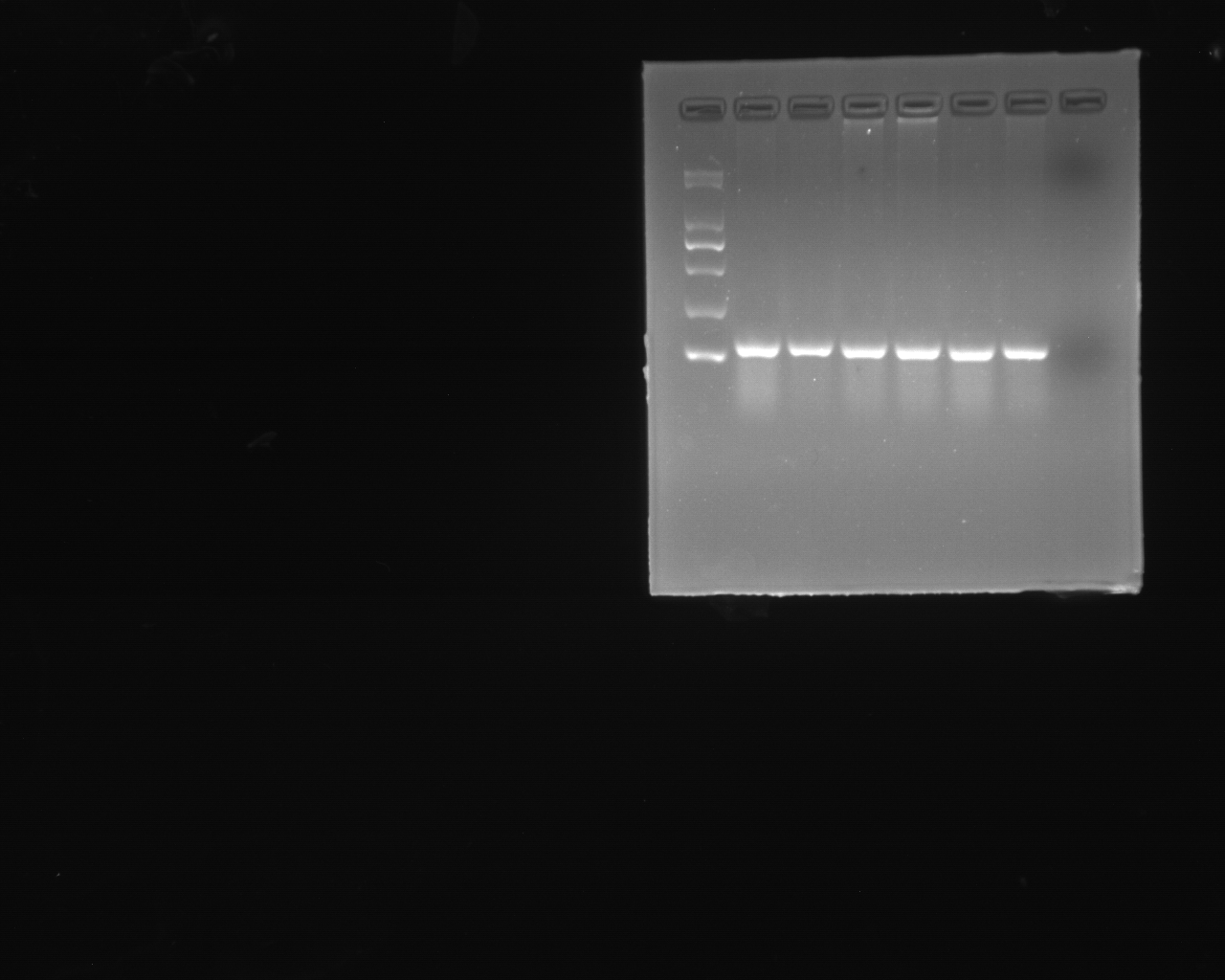

Supplement: S2 File — (ZIP) [file pone.0326317.s002.zip › Supporting Information PCR data/OAT3/2022-11-13 OAT3 3 cheng.tif]

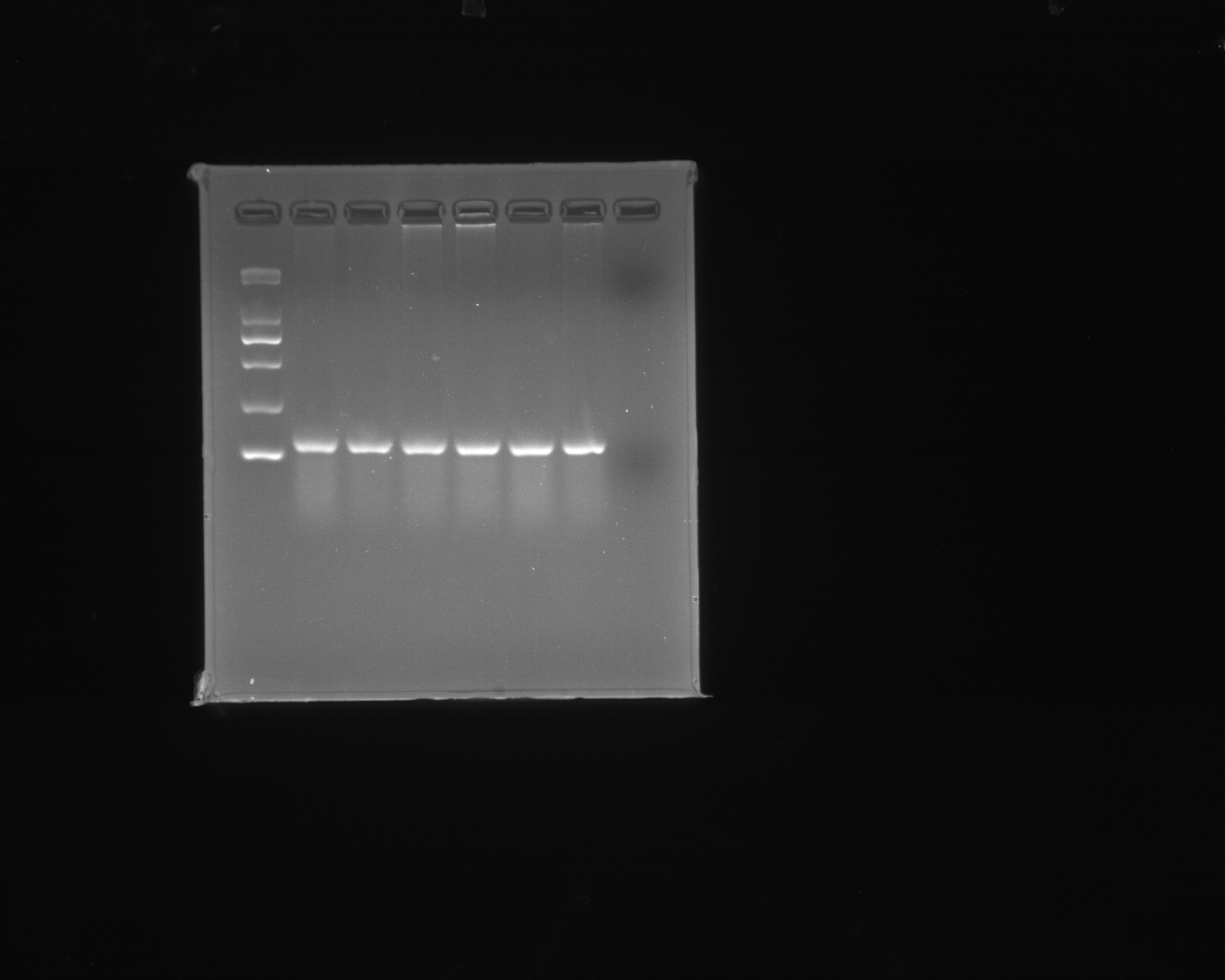

Supplement: S2 File — (ZIP) [file pone.0326317.s002.zip › Supporting Information PCR data/OAT3/2022-11-14 OAT3 2成.tif]

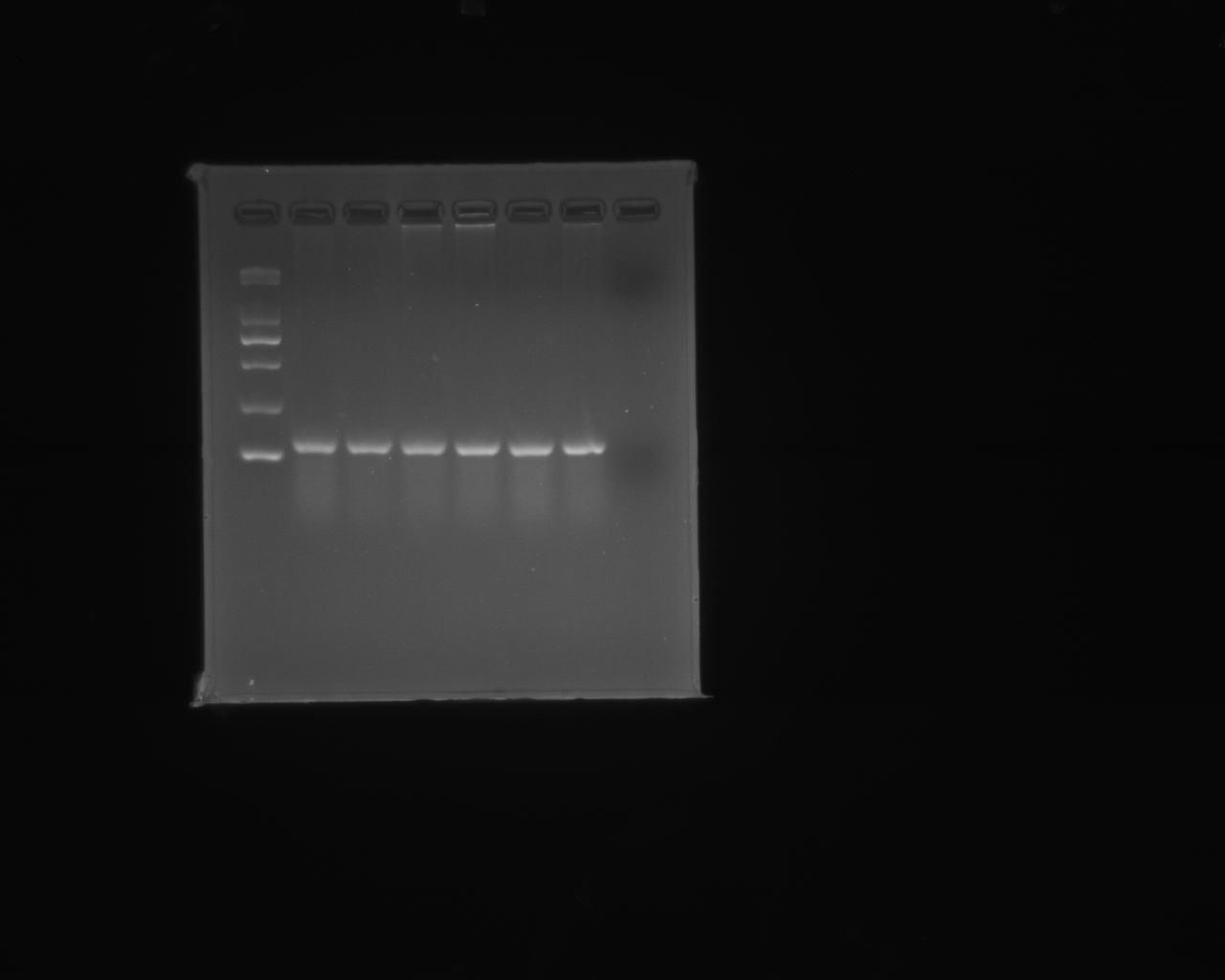

Supplement: S2 File — (ZIP) [file pone.0326317.s002.zip › Supporting Information PCR data/OAT3/2022-11-14 OAT3 3成.tif]

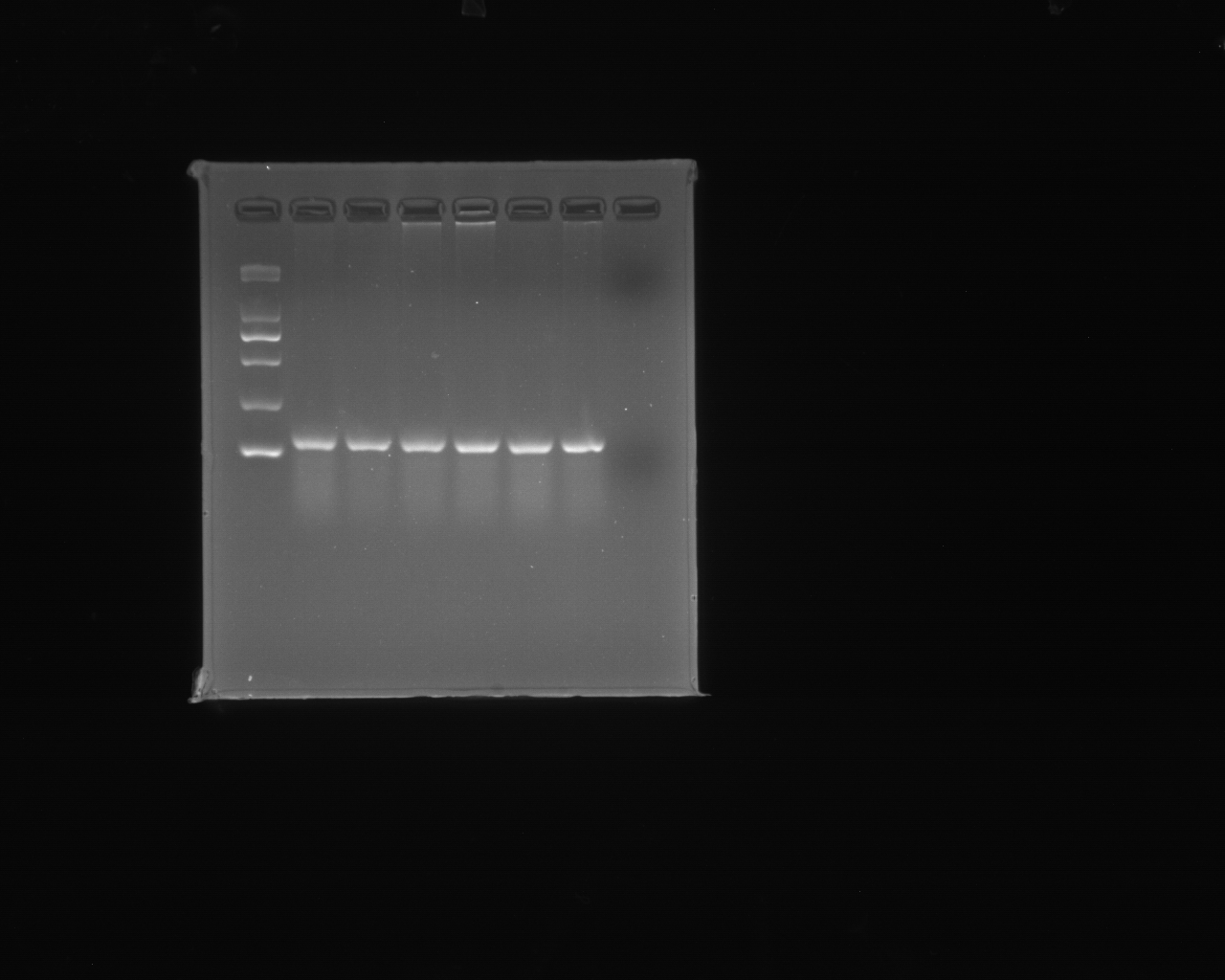

Supplement: S2 File — (ZIP) [file pone.0326317.s002.zip › Supporting Information PCR data/OAT3/2022-11-14 OAT3 成.tif]

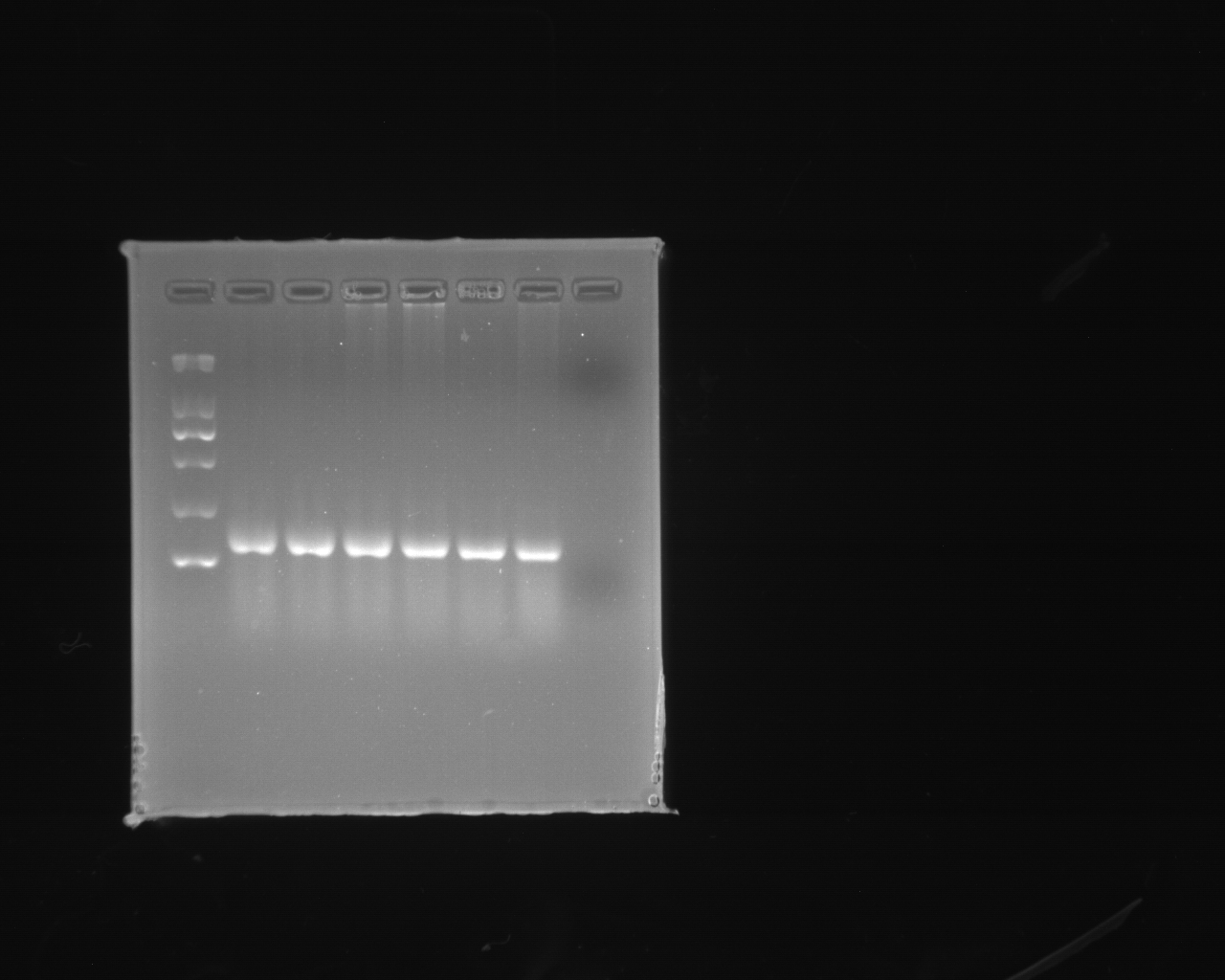

Supplement: S2 File — (ZIP) [file pone.0326317.s002.zip › Supporting Information PCR data/OAT3/2022-11-2 GAPDH2调 2.tif]

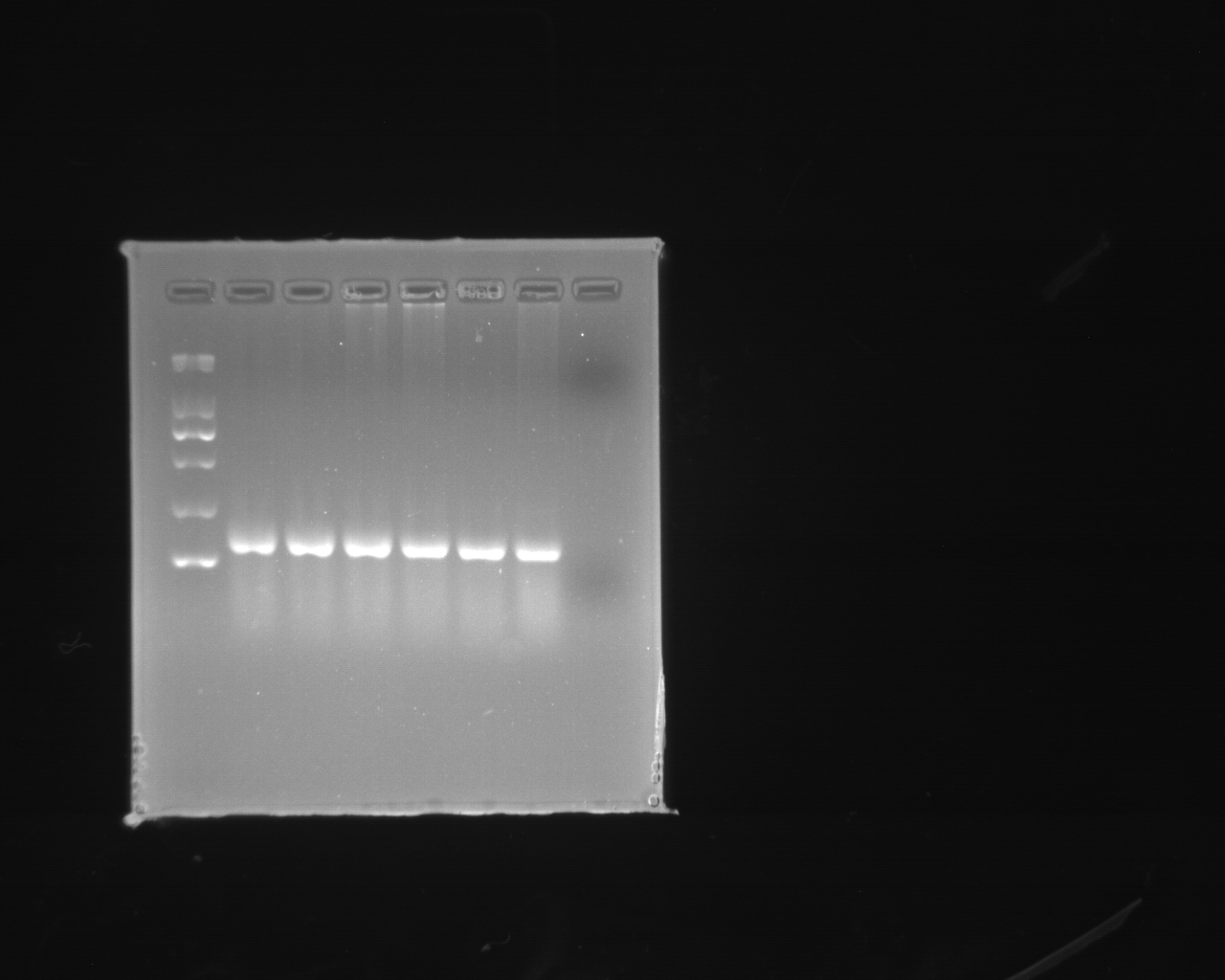

Supplement: S2 File — (ZIP) [file pone.0326317.s002.zip › Supporting Information PCR data/OAT3/2022-11-2 GAPDH2调 4.tif]

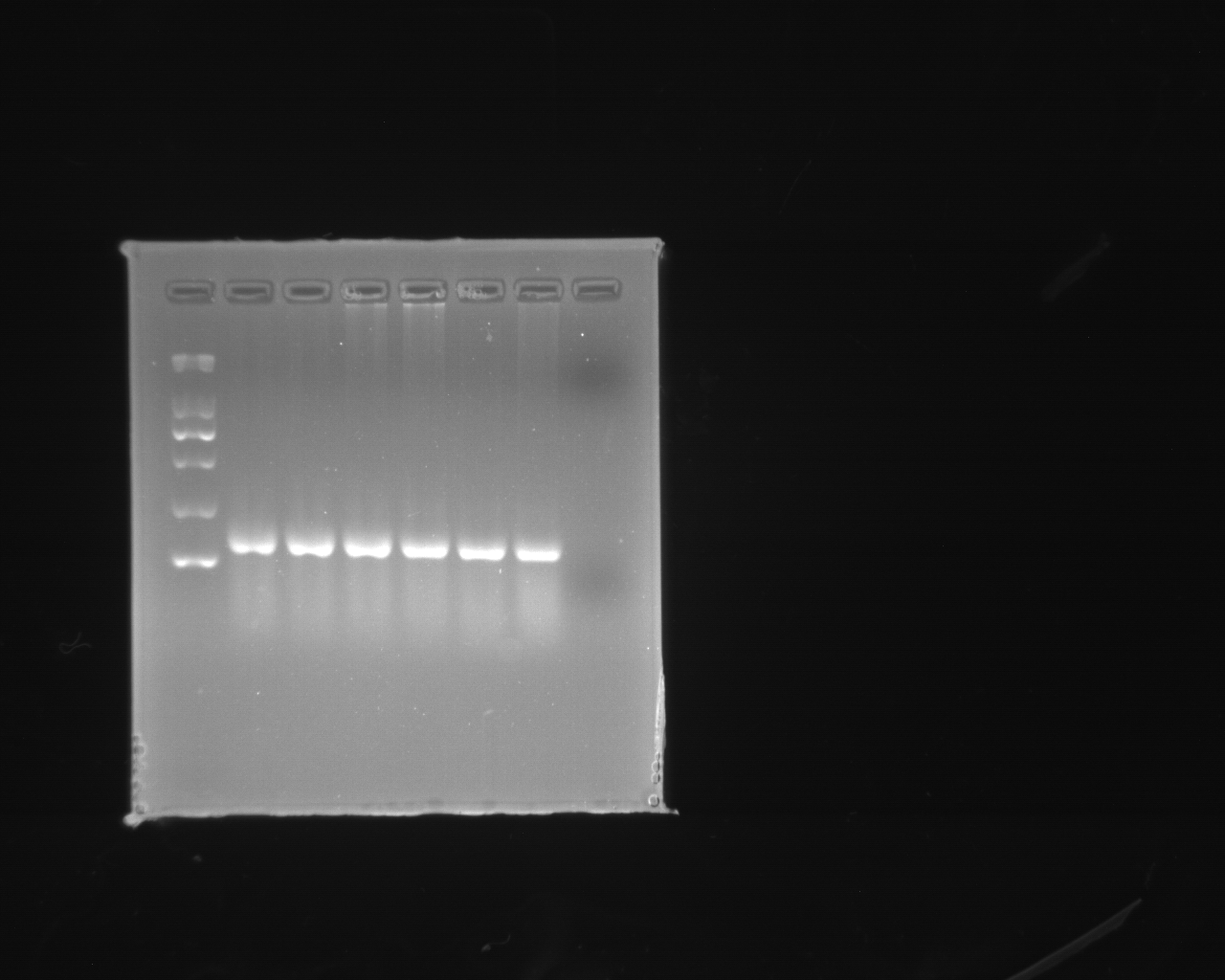

Supplement: S2 File — (ZIP) [file pone.0326317.s002.zip › Supporting Information PCR data/OAT3/2022-11-2 GAPDH2调 5.tif]

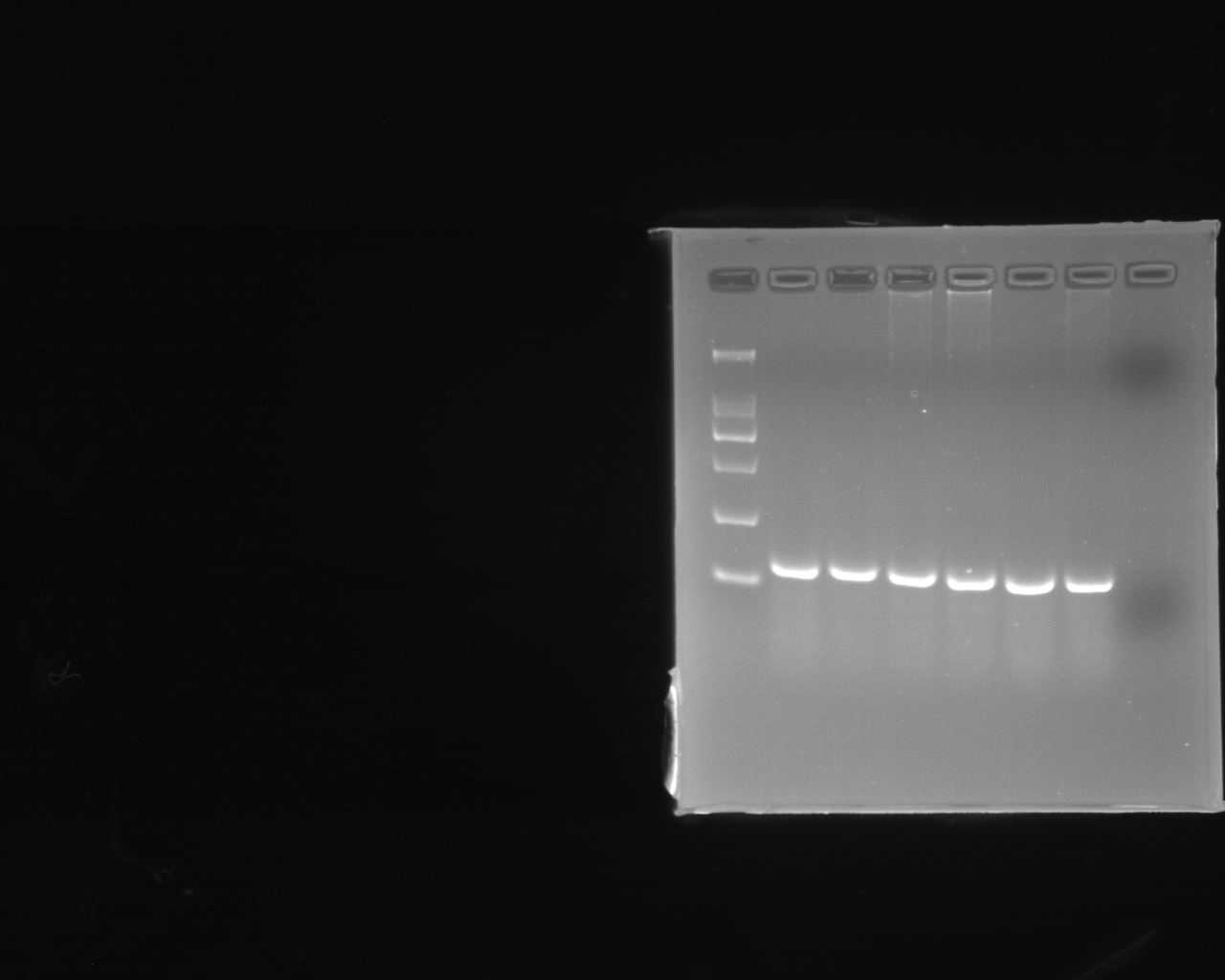

Supplement: S2 File — (ZIP) [file pone.0326317.s002.zip › Supporting Information PCR data/OAT3/2022-11-2 OAT3 调2.tif]

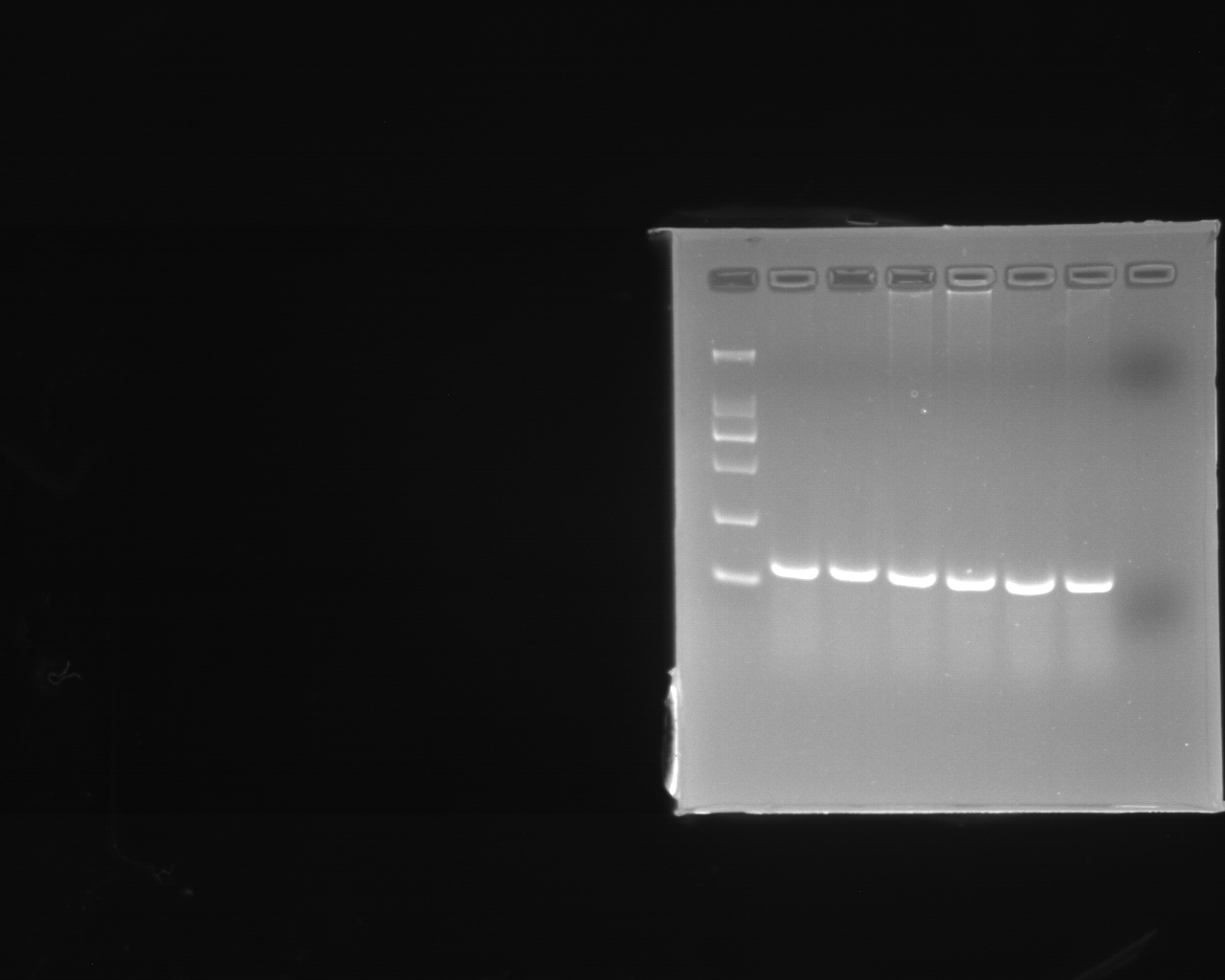

Supplement: S2 File — (ZIP) [file pone.0326317.s002.zip › Supporting Information PCR data/OAT3/2022-11-2 OAT3 调4.tif]

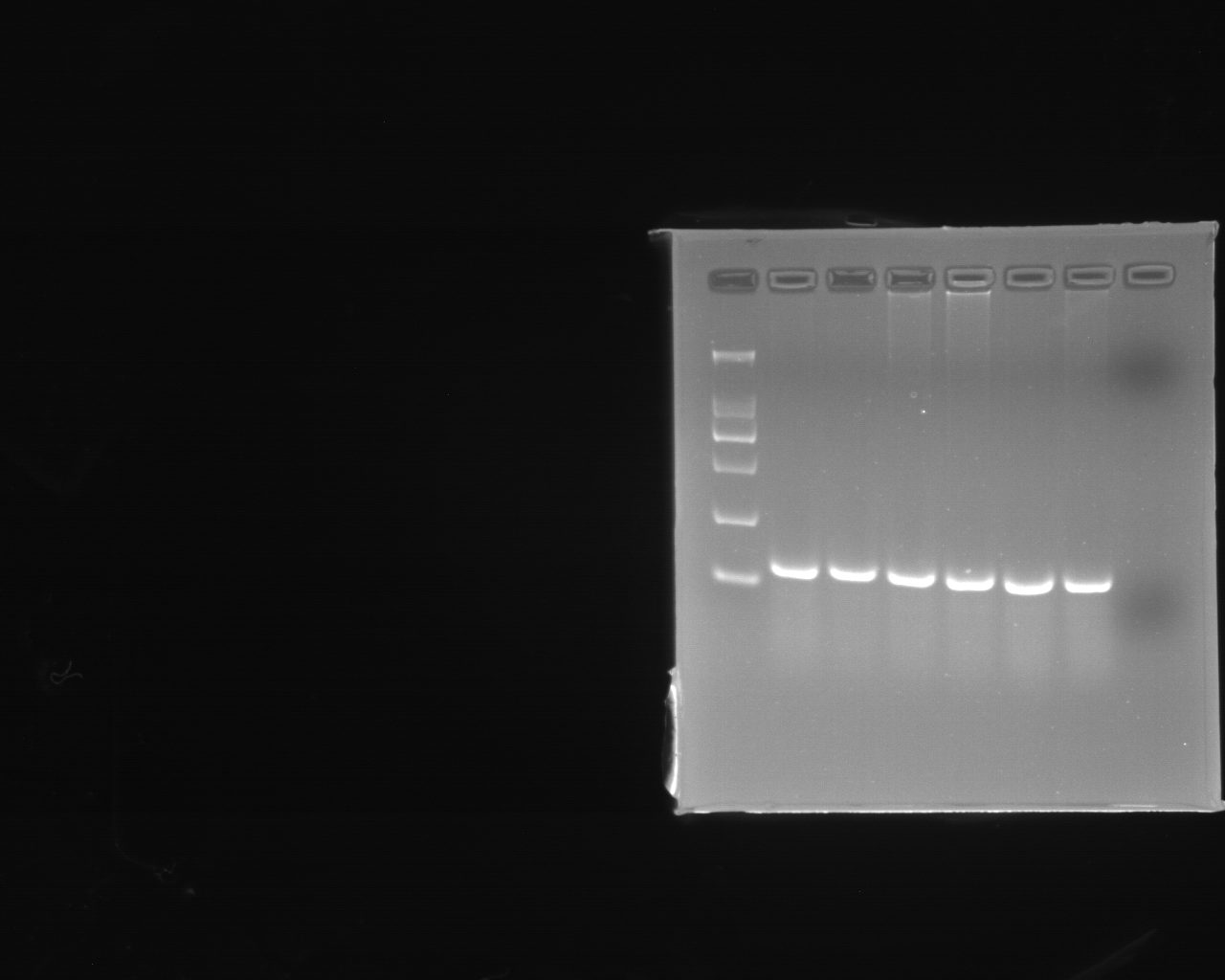

Supplement: S2 File — (ZIP) [file pone.0326317.s002.zip › Supporting Information PCR data/OAT3/2022-11-2 OAT3 调6.tif]

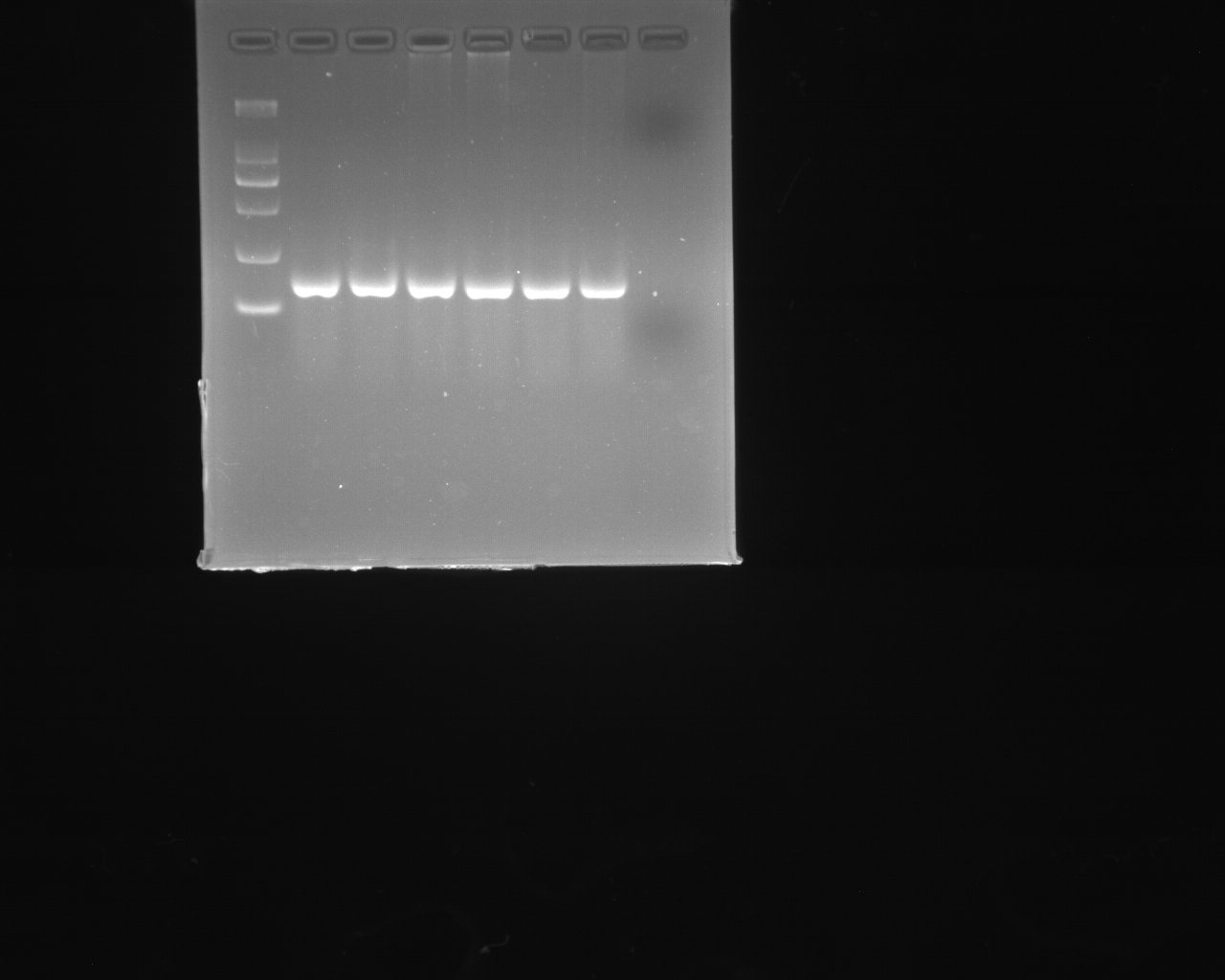

Supplement: S2 File — (ZIP) [file pone.0326317.s002.zip › Supporting Information PCR data/OAT3/2023.3.1/2022-11-4 GAPDH2 5U 1.tif]

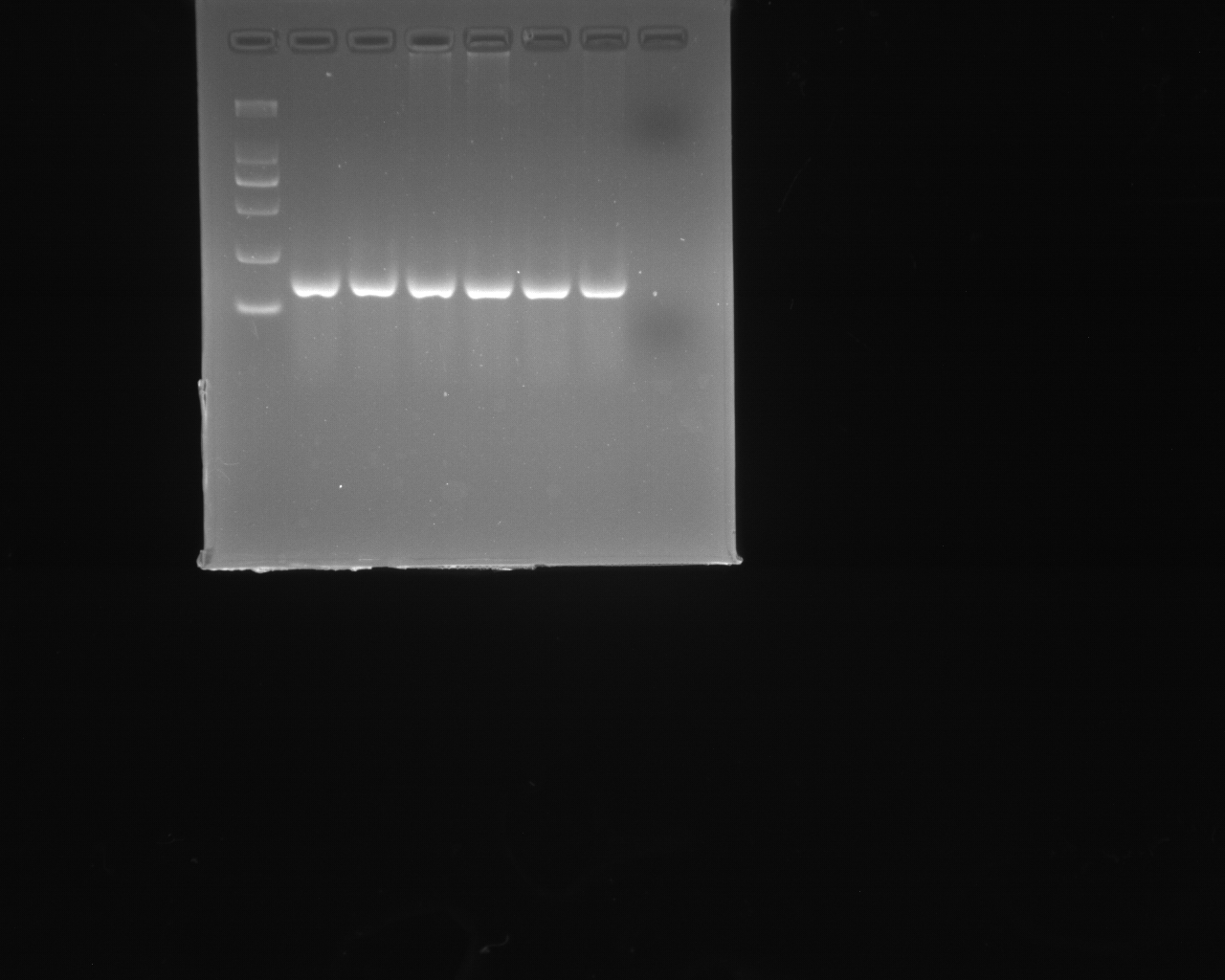

Supplement: S2 File — (ZIP) [file pone.0326317.s002.zip › Supporting Information PCR data/OAT3/2023.3.1/2022-11-4 GAPDH2 5U 2.tif]

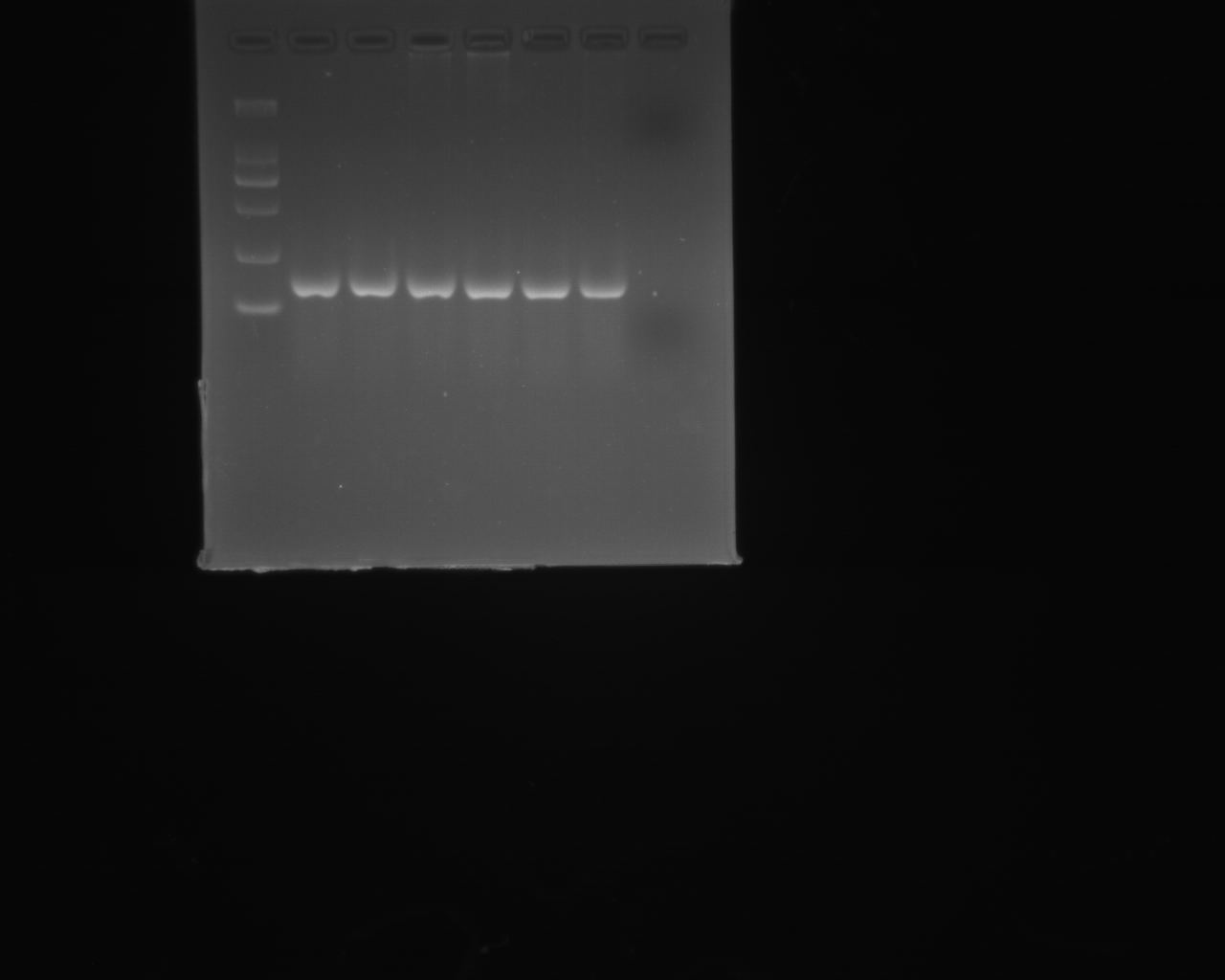

Supplement: S2 File — (ZIP) [file pone.0326317.s002.zip › Supporting Information PCR data/OAT3/2023.3.1/2022-11-4 GAPDH2 5U 3.tif]

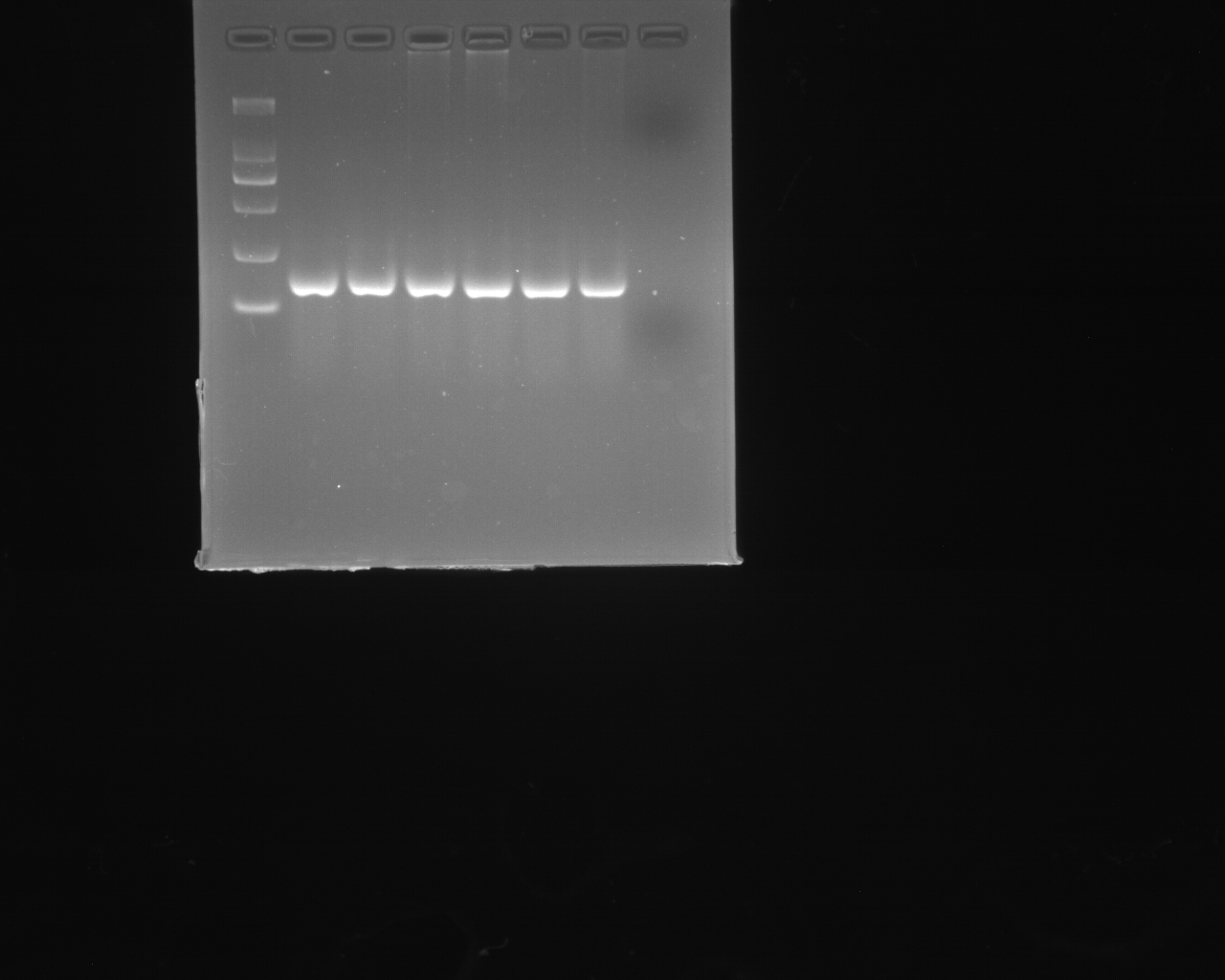

Supplement: S2 File — (ZIP) [file pone.0326317.s002.zip › Supporting Information PCR data/OAT3/2023.3.1/2022-11-4 GAPDH2 5U 6.tif]

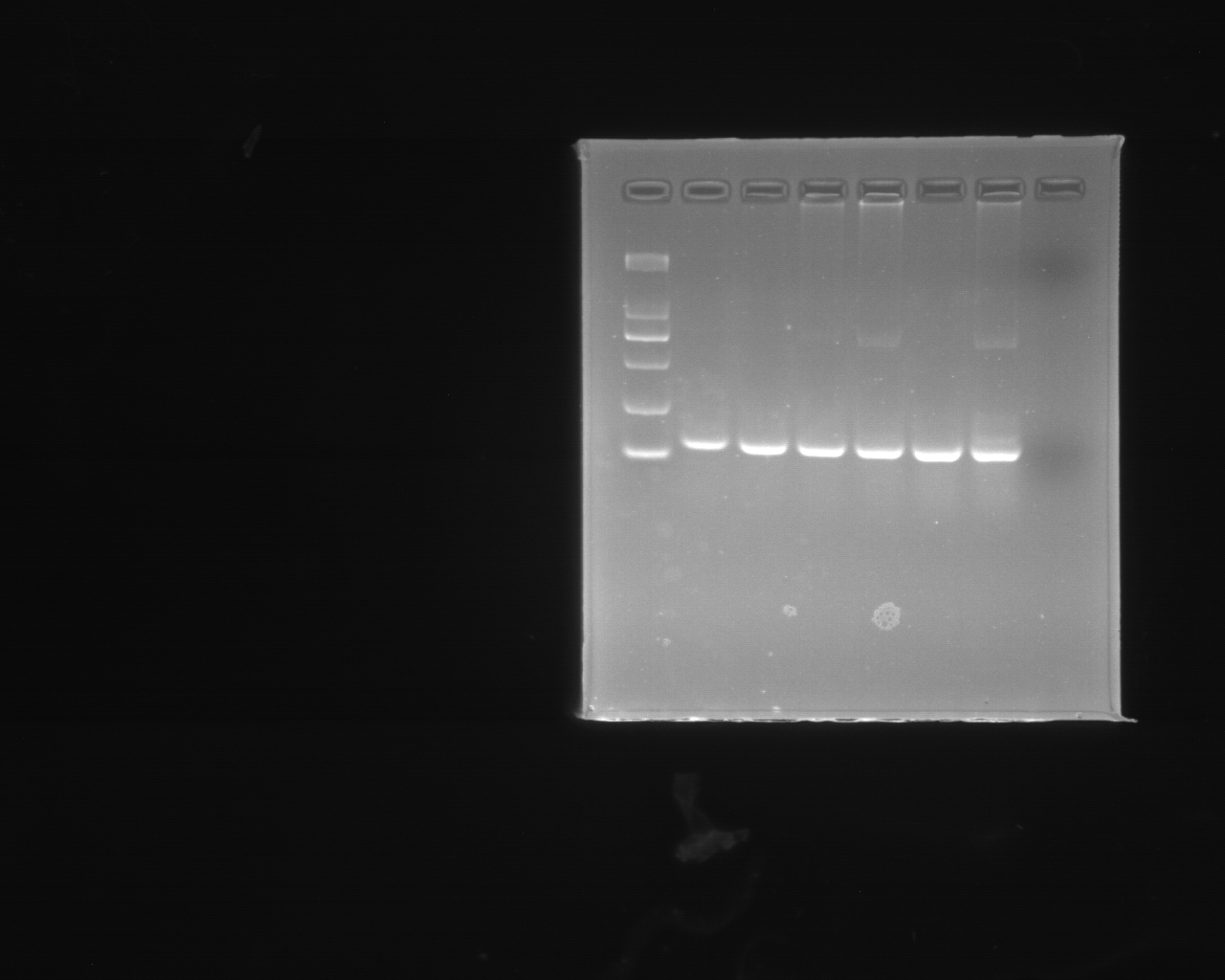

Supplement: S2 File — (ZIP) [file pone.0326317.s002.zip › Supporting Information PCR data/URAT1/2022-11-5 URAT1 2-3.tif]

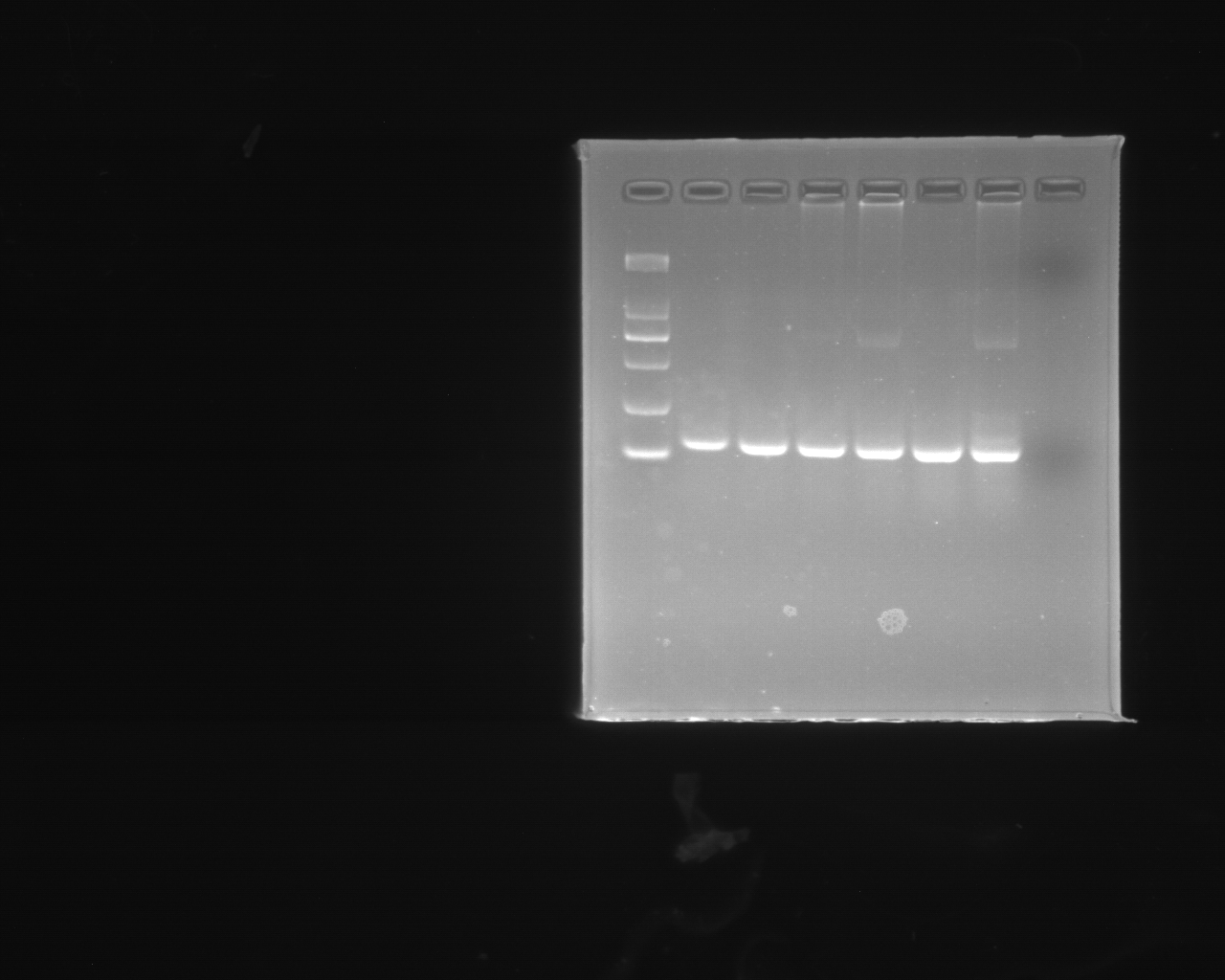

Supplement: S2 File — (ZIP) [file pone.0326317.s002.zip › Supporting Information PCR data/URAT1/2022-11-5 URAT1 2-4.tif]

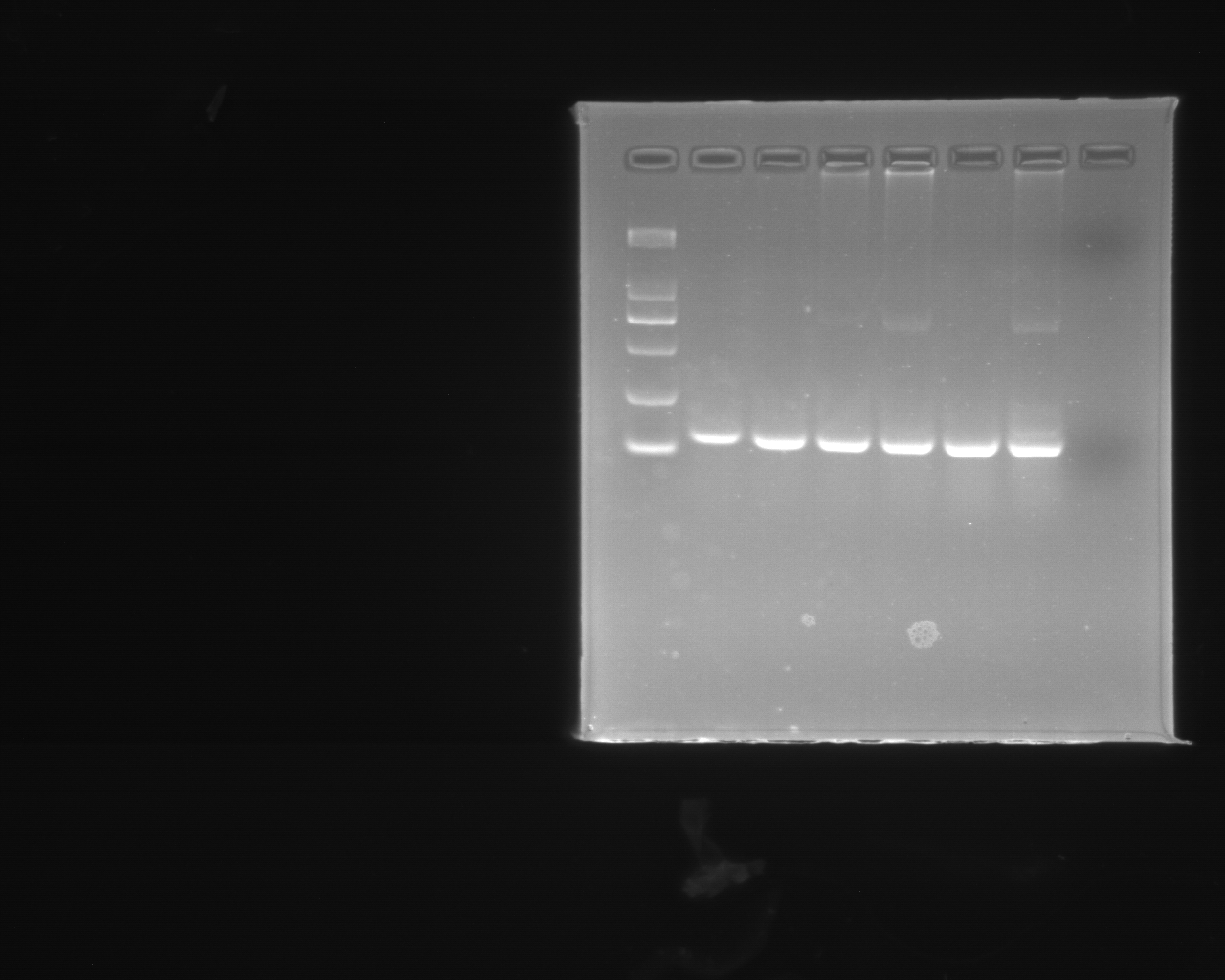

Supplement: S2 File — (ZIP) [file pone.0326317.s002.zip › Supporting Information PCR data/URAT1/2022-11-5 URAT1 2-6.tif]

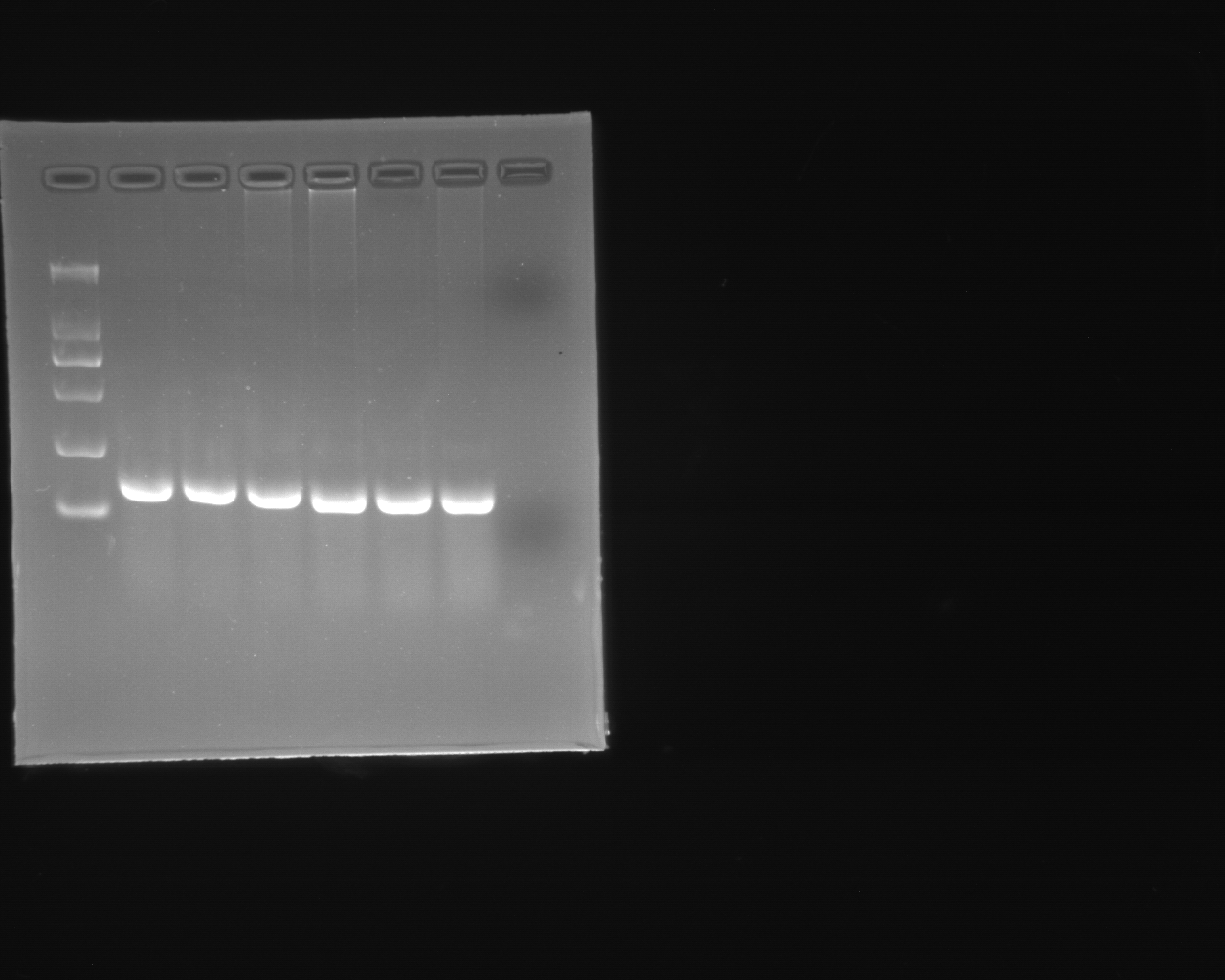

Supplement: S2 File — (ZIP) [file pone.0326317.s002.zip › Supporting Information PCR data/URAT1/2022-11-7 GAPDH 2-1.tif]

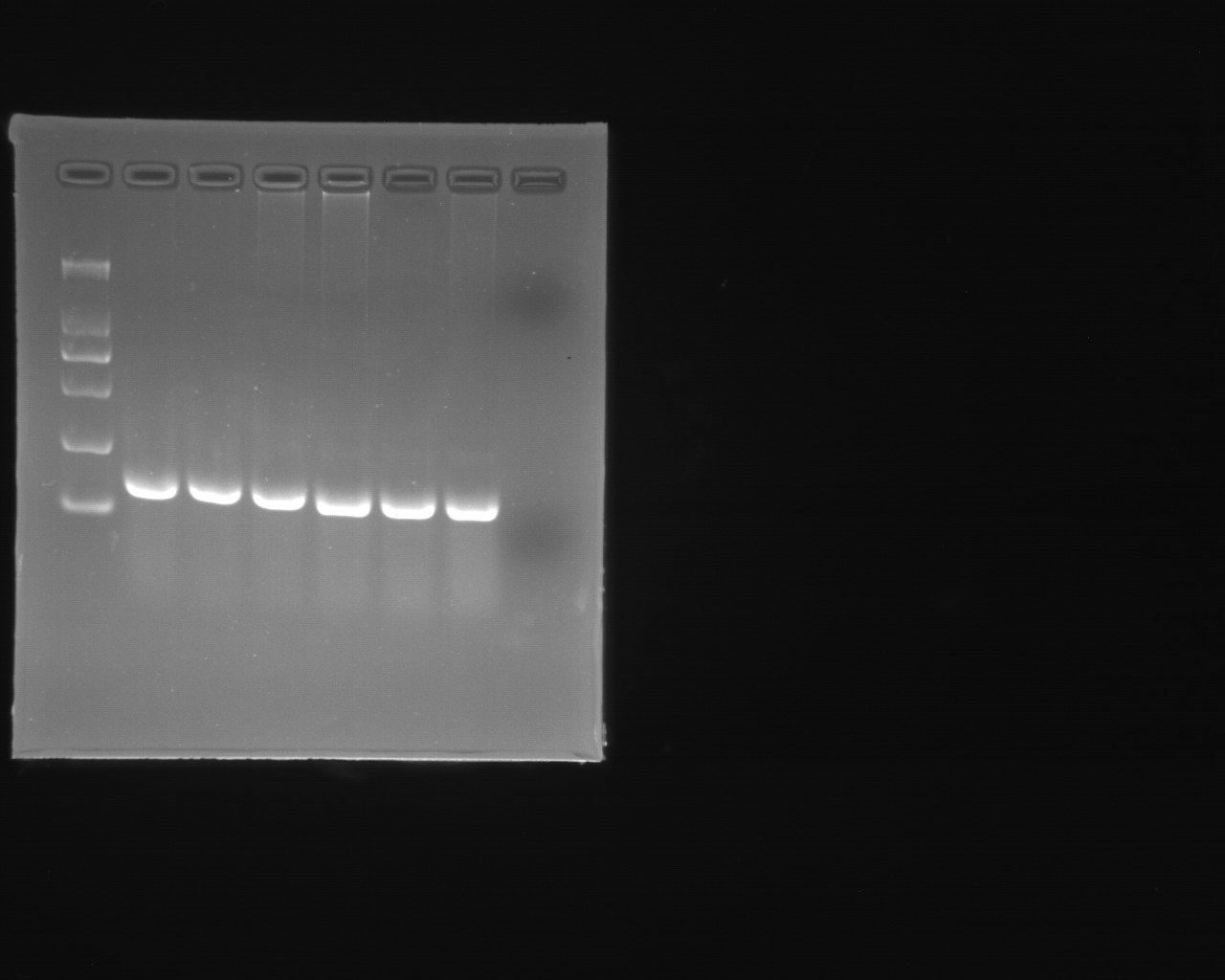

Supplement: S2 File — (ZIP) [file pone.0326317.s002.zip › Supporting Information PCR data/URAT1/2022-11-7 GAPDH 2-2.tif]

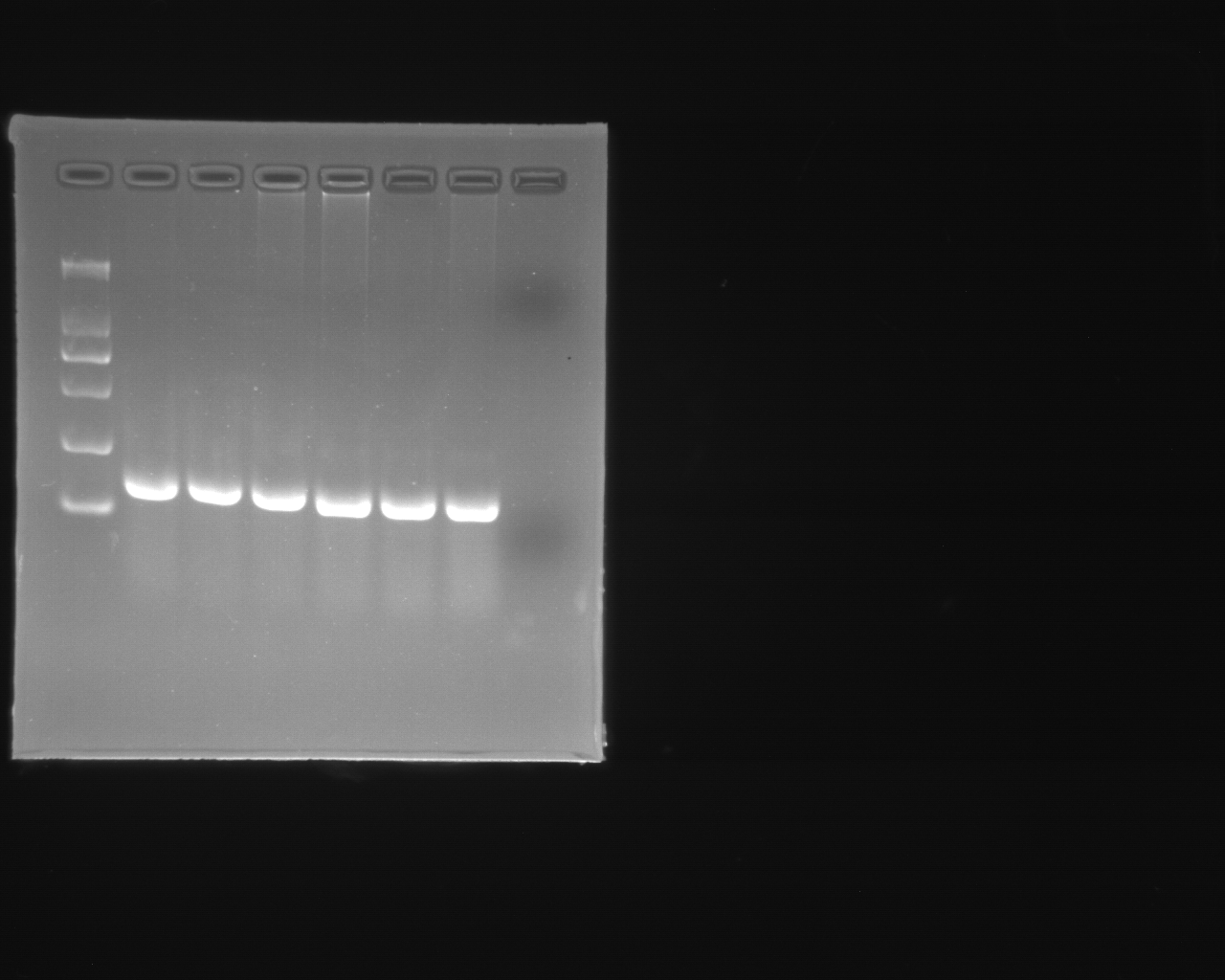

Supplement: S2 File — (ZIP) [file pone.0326317.s002.zip › Supporting Information PCR data/URAT1/2022-11-7 GAPDH 2-3.tif]

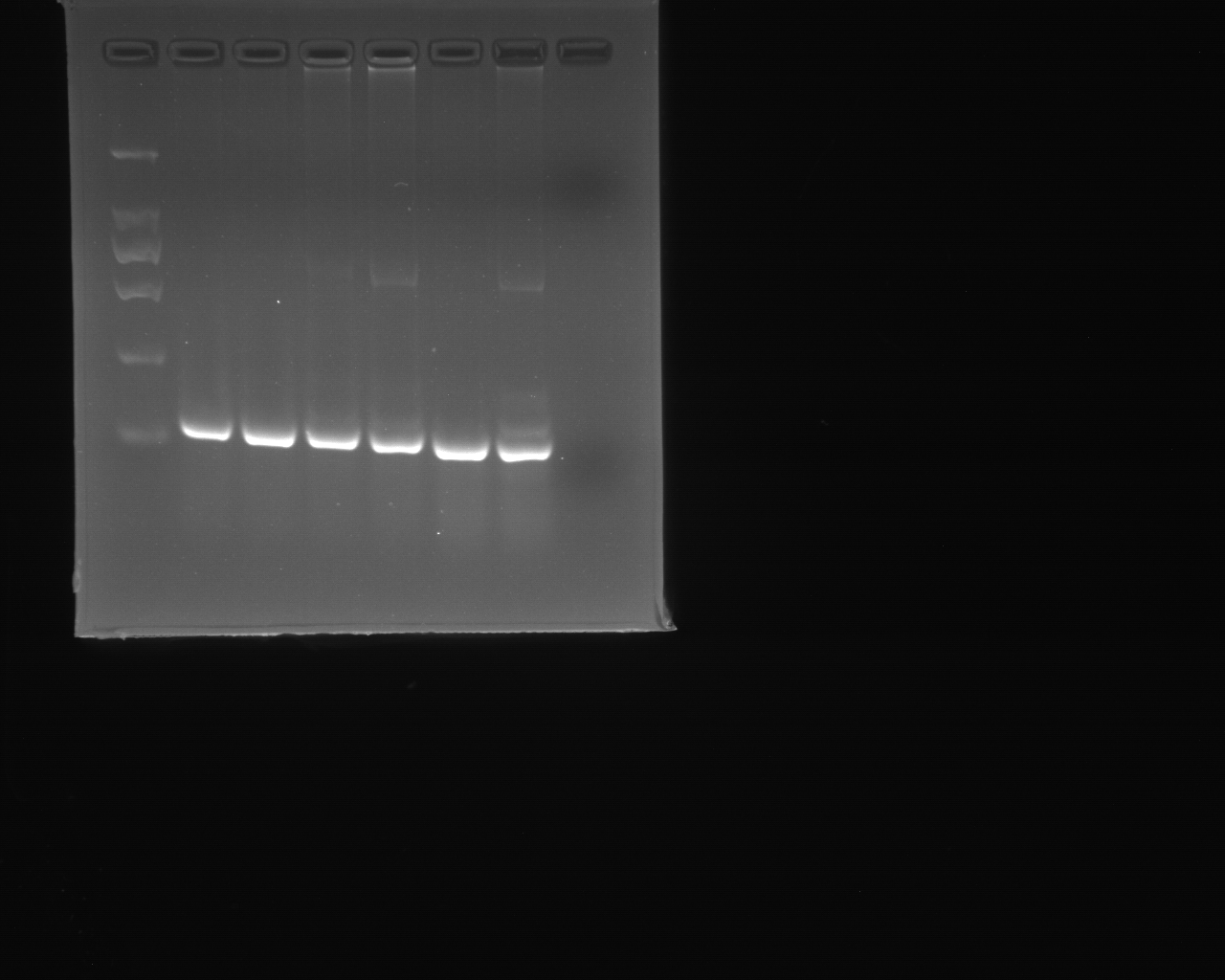

Supplement: S2 File — (ZIP) [file pone.0326317.s002.zip › Supporting Information PCR data/URAT1/2022-11-7 URAT1 2-11.tif]

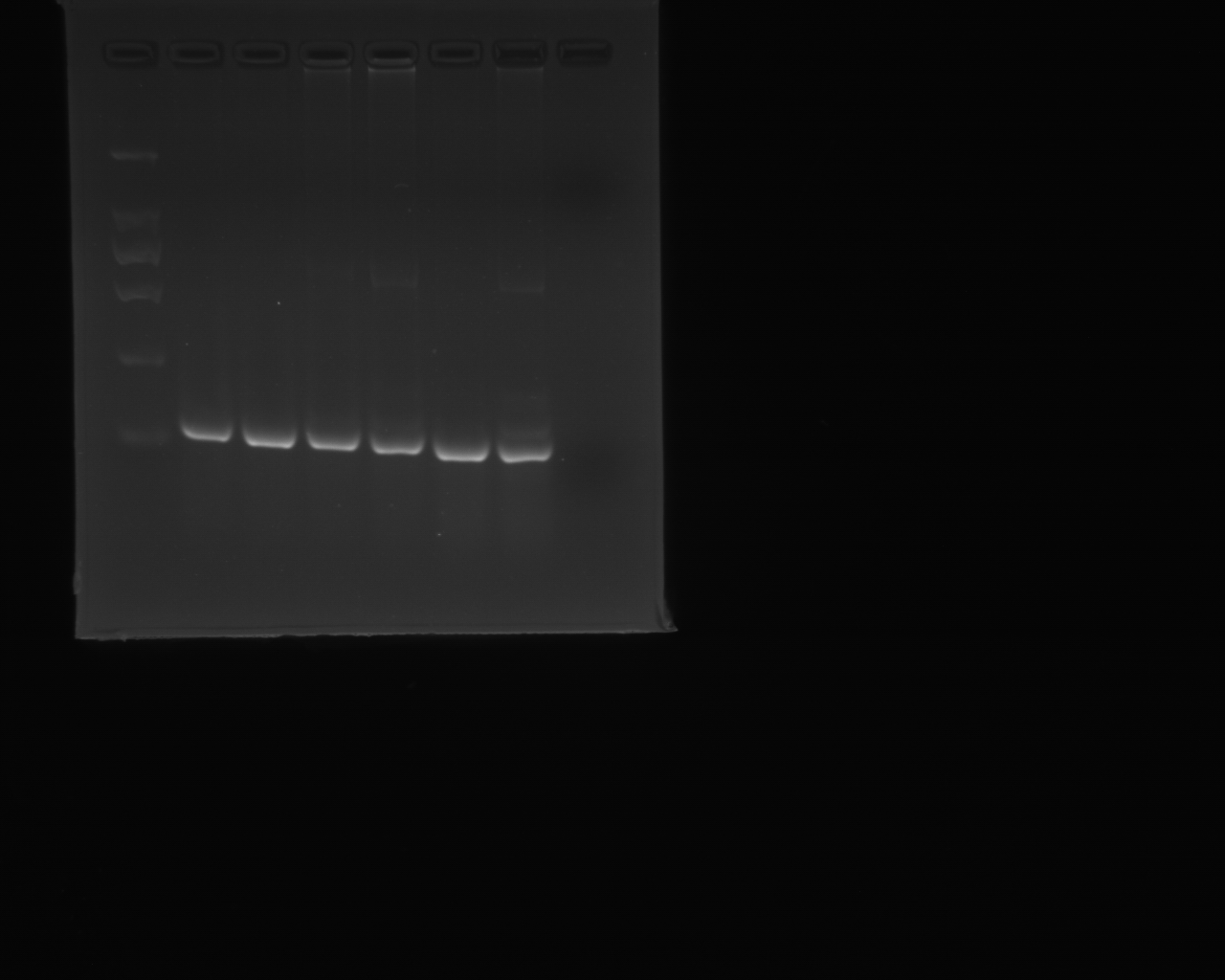

Supplement: S2 File — (ZIP) [file pone.0326317.s002.zip › Supporting Information PCR data/URAT1/2022-11-7 URAT1 2-12.tif]

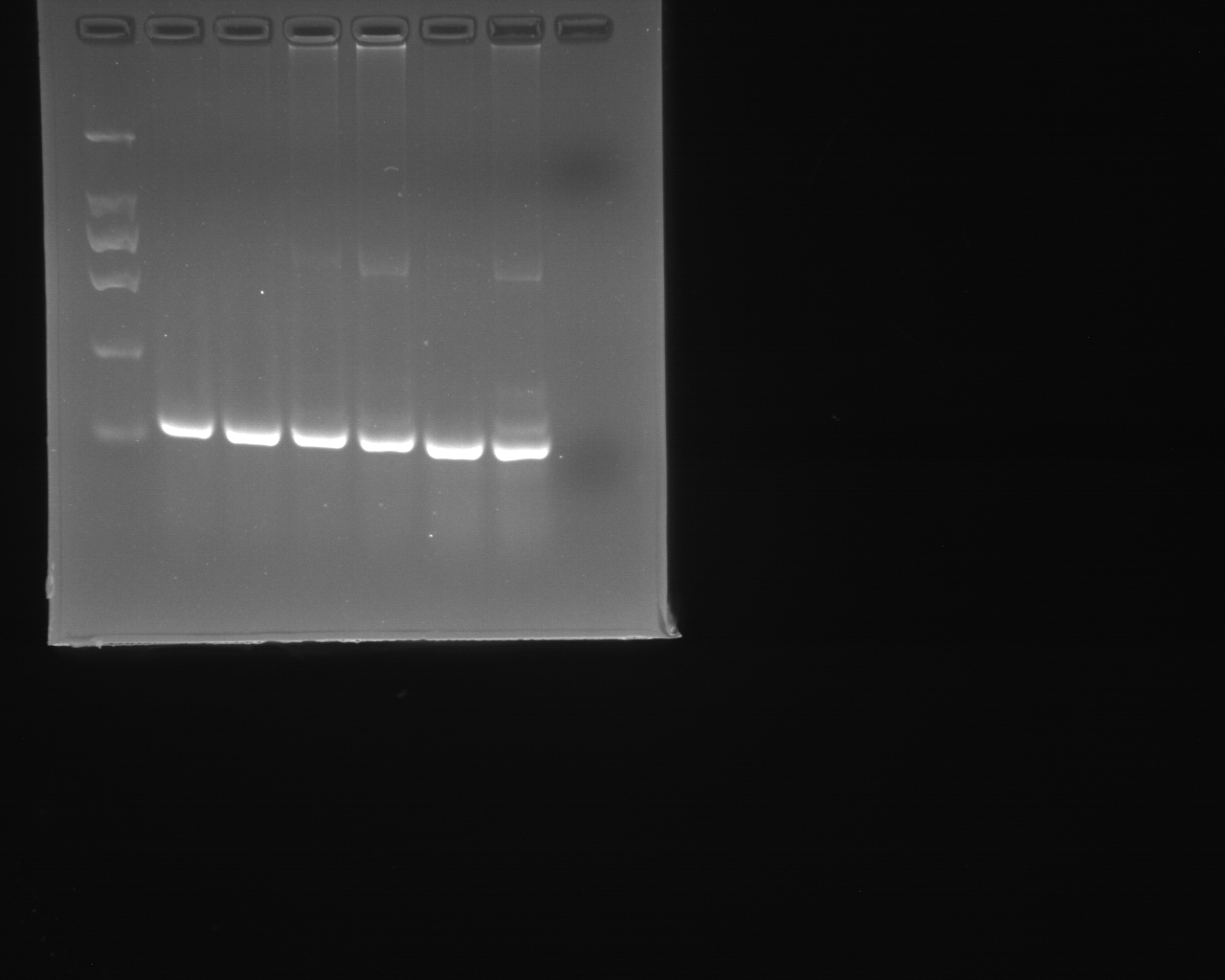

Supplement: S2 File — (ZIP) [file pone.0326317.s002.zip › Supporting Information PCR data/URAT1/2022-11-7 URAT1 2-13.tif]

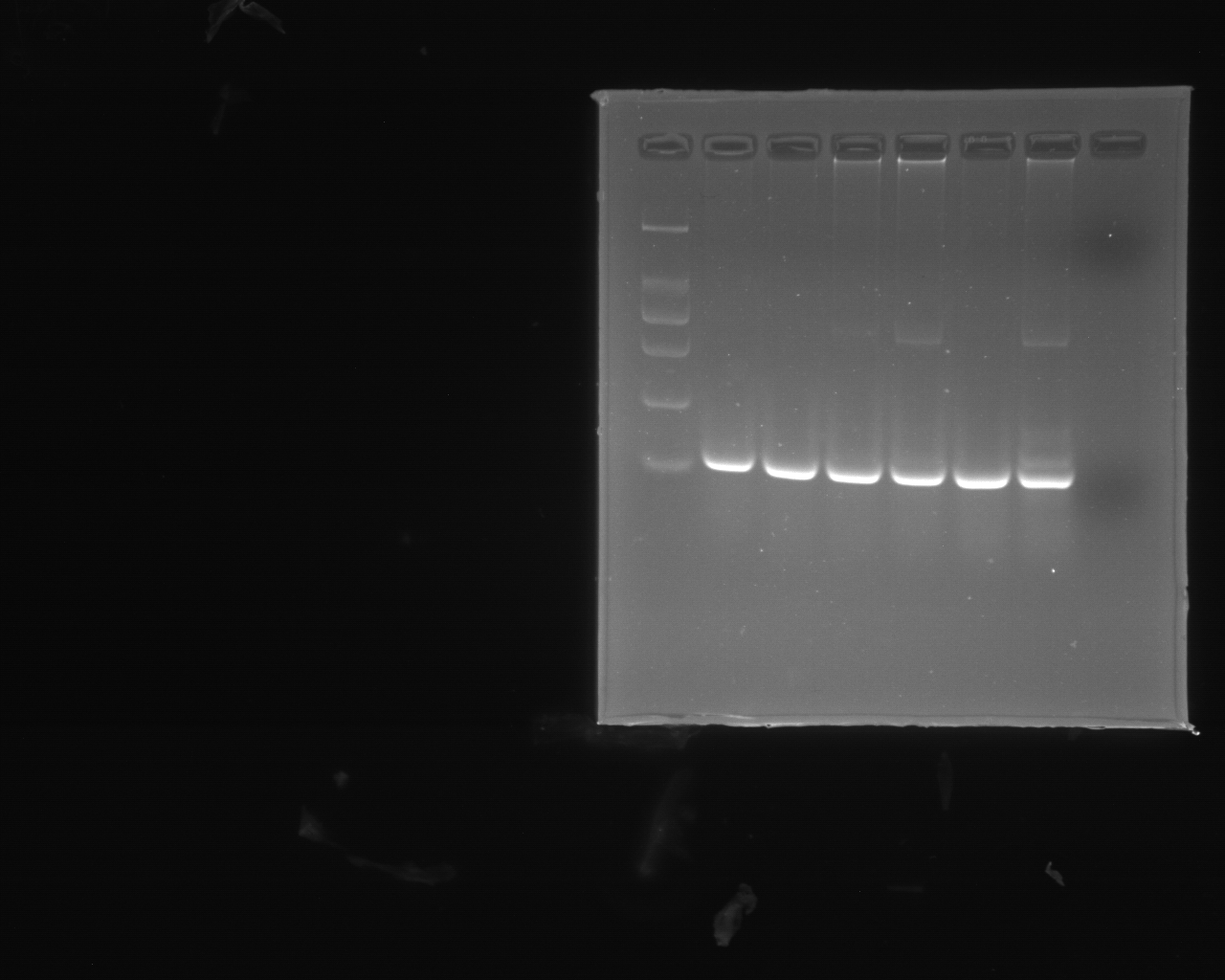

Supplement: S2 File — (ZIP) [file pone.0326317.s002.zip › Supporting Information PCR data/URAT1/2022-11-7 URAT1 3.tif]

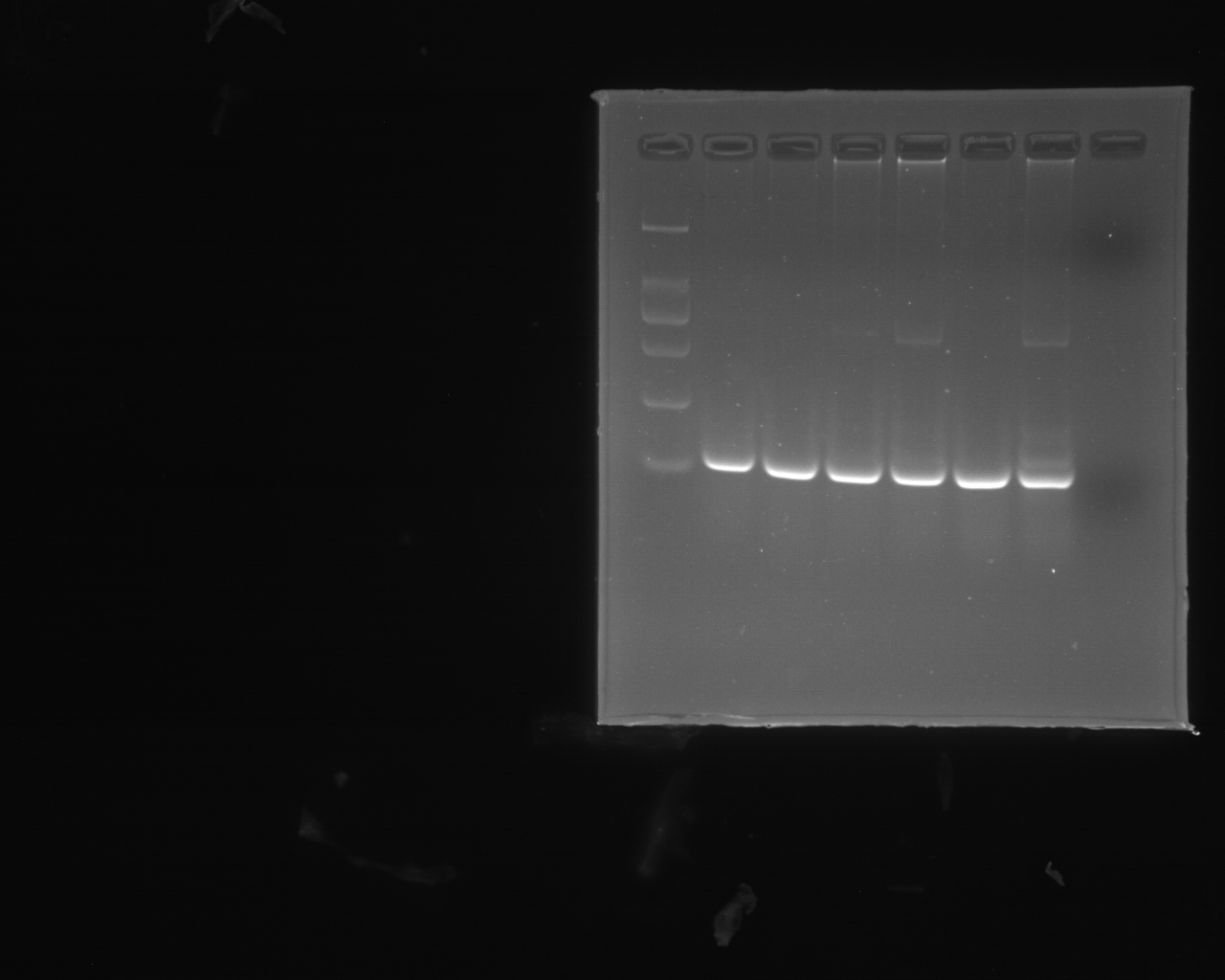

Supplement: S2 File — (ZIP) [file pone.0326317.s002.zip › Supporting Information PCR data/URAT1/2022-11-7 URAT1 4.tif]

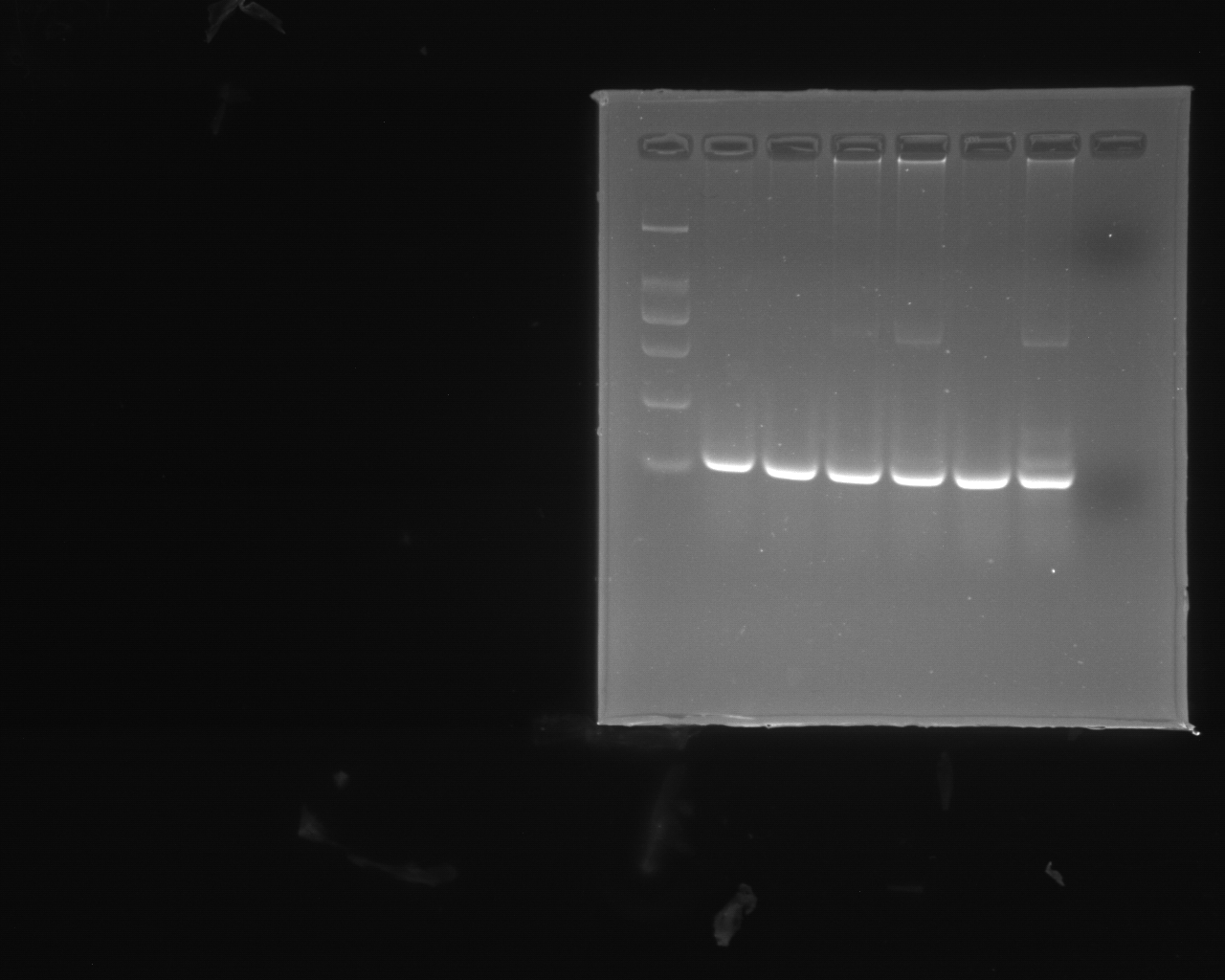

Supplement: S2 File — (ZIP) [file pone.0326317.s002.zip › Supporting Information PCR data/URAT1/2022-11-7 URAT1 6.tif]
