# Supplementary material for: Hederagenin’s uric acid-lowering effects in hyperuricemic mice: Mechanistic insights from molecular docking and in vivo analysis
Source: PLoS One. 2025 Jun 24;20(6):e0326317. doi: 10.1371/journal.pone.0326317 (PMC12186911; doi:10.1371/journal.pone.0326317)
Supplement: Attachment — Submitted filename: Reply to editorial office.docx [file pone.0326317.s004.docx]

**Reply to editorial office’s comments**

PONE-D-24-51322R1
Hederagenin's Uric Acid-Lowering Effects in Hyperuricemic Mice: Mechanistic Insights from Molecular Docking and In Vivo Analysis

Dear Dr. Bai,
We've checked your submission and before we can proceed, we need you to address the following issues:

1. Please note that funding information should not appear in any section or other areas of your manuscript. We will only publish funding information present in the Funding Statement section of the online submission form. Please remove any funding-related text from the manuscript.
**Response**: We have removed funding information in the revised manuscript.

2. Please note that your Data Availability Statement is currently missing the repository name and/or the DOI/accession number of each dataset OR a direct link to access each database. If your manuscript is accepted for publication, you will be asked to provide these details on a very short timeline. We therefore suggest that you provide this information now, though we will not hold up the peer review process if you are unable.
**Response**:We have provided blot/gel image data are in **Supporting Information/Blot/gel Image Data**.

3. Please include a copy of Table 1 which you refer to in your text on page 9.
**Response**: We have include a copy of Table 1 on page 9 as **Supporting Information Table 1**.

4. PLOS ONE now requires that authors provide the original uncropped and unadjusted images underlying all blot or gel results reported in a submission’s figures or Supporting Information files. This policy and the journal’s other requirements for blot/gel reporting and figure preparation are described in detail at https://journals.plos.org/plosone/s/figures#loc-blot-and-gel-reporting-requirements and https://journals.plos.org/plosone/s/figures#loc-preparing-figures-from-image-files. When you submit your revised manuscript, please ensure that your figures adhere fully to these guidelines and provide the original underlying images for all blot or gel data reported in your submission. See the following link for instructions on providing the original image data:

https://journals.plos.org/plosone/s/figures#loc-original-images-for-blots-and-gels.
**Response**:We have provided all original uncropped and unadjusted images underlying all blot or gel results reported in a submission’s figures.

(Please refer to **Supporting Information/Blot/gel Image Data**)

In your cover letter, please note whether your blot/gel image data are in Supporting Information or posted at a public data repository, provide the repository URL if relevant, and provide specific details as to which raw blot/gel images, if any, are not available. Email us at plosone@plos.org if you have any questions.
**Response**: We have noted that all blot/gel image data are in **Supporting Information/Blot/gel Image Data**.

5.Please provide additional information regarding the details on (1) methods of sacrifice (chemical/drug used), (2) methods of anesthesia and/or analgesia (chemical/drug used).

**Response**: We have provided additional information regarding the details on (1) methods of sacrifice (chemical/drug used), (2) methods of anesthesia and/or analgesia (chemical/drug used).

(Please refer to **2.3 Experimental Animals/3^rd^, 4^th^, 5^th^ and 6^th^ paragraphs**)
